# Asymptotic Analysis of Optimal Vaccination Policies

**DOI:** 10.1007/s11538-022-01114-3

**Published:** 2023-01-20

**Authors:** Matthew J. Penn, Christl A. Donnelly

**Affiliations:** 1 Department of Statistics, University of Oxford, St Giles’, Oxford, OX1 3LB UK; 2 Department of Infectious Disease Epidemiology, Imperial College London, South Kensington Campus, London, SW7 2AZ UK

**Keywords:** Vaccination, Epidemiology, Epidemics, SIR modelling

## Abstract

Targeted vaccination policies can have a significant impact on the number of infections and deaths in an epidemic. However, optimising such policies is complicated, and the resultant solution may be difficult to explain to policy-makers and to the public. The key novelty of this paper is a derivation of the leading-order optimal vaccination policy under multi-group susceptible–infected–recovered dynamics in two different cases. Firstly, it considers the case of a small vulnerable subgroup in a population and shows that (in the asymptotic limit) it is optimal to vaccinate this group first, regardless of the properties of the other groups. Then, it considers the case of a small vaccine supply and transforms the optimal vaccination problem into a simple knapsack problem by linearising the final size equations. Both of these cases are then explored further through numerical examples, which show that these solutions are also directly useful for realistic parameter values. Moreover, the findings of this paper give some general principles for optimal vaccination policies which will help policy-makers and the public to understand the reasoning behind optimal vaccination programs in more generic cases.

## Introduction

The trajectory of an epidemic can be dramatically changed by the implementation of a vaccination program, as has been shown in the case of COVID-19 (Bloom et al. 2021). These vaccination programs are most effective when they target specific groups in a population (Fitzpatrick and Galvani 2021), although the optimal targeting strategy is dependent on the properties of the disease and vaccine (Moore et al. 2021). Thus, it is important to have robust methods to determine the optimal strategy whenever a new epidemic emerges.

In recent years, the epidemiological literature has grown rapidly, and a wide range of models have been developed and analysed. These include branching-process models (Pakkanen et al. 2021); network-based models (Bedson et al. 2021); and machine-learning-based models (Muhammad et al. 2021), among many others (Brauer et al. 2019).

However, despite these innovations, compartmental models, where the population is split into a number of subgroups and disease transmission is modelled by a system of differential equations (Abou-Ismail 2020), remain a popular choice for epidemiologists and have been widely used for modelling the COVID-19 pandemic (Kong et al. 2022). As discussed in (Kong et al. 2022), a number of different compartment structures have been used, while many authors have also sought to model the effect of government interventions and quarantining procedures (Vardavas et al. 2021; de Camino-Beck 2020; Adhikari et al. 2020).

One such compartmental model that is widely used (Ram and Schaposnik 2021; Acemoglu et al. 2021; Kuniya 2019) is the multi-group SIR (susceptible–infected–recovered) model. This is an extension of the classical SIR model (Kermack and McKendrick 1927) and has been used to model a range of diseases such as measles (Sattenspiel and Dietz 1995), influenza (Brauer 2008) and COVID-19 (Ellison 2020). It provides a general framework with which to assess the effectiveness of different vaccination policies, while also remaining mathematically tractable, allowing theorems about its behaviour to be rigorously proved (Penn and Donnelly 2022). It splits a population up into a number of interconnected subgroups (such as age groups (Longini Jr et al. 1978) and captures the different transmission dynamics between each group. This construction highlights the dual benefit that vaccination can have—vaccines that are infection-reducing directly protect the individuals that are vaccinated while transmission-reducing vaccines can also indirectly protect unvaccinated individuals (Eichner et al. 2017).

This dual benefit can significantly complicate the optimal vaccination problem when there is a negative correlation between the infectiousness of a group and the vulnerability of its members to the disease. Examples of this occur when the population is divided by age for diseases such as COVID-19 (Miura et al. 2021) and seasonal influenza (Molinari et al. 2007). In such cases, the optimal strategy may not be obvious and could be highly dependent on uncertain parameters (Saadi et al. 2021), while the seemingly intuitive solution may be significantly sub-optimal (Delmas et al. 2021). Moreover, the complicated methods used to find the optimal solution, involving solving the adjoint equations derived via Pontryagin’s maximum principle (Boutayeb et al. 2021; Lee et al. 2012), mean that the optimal solution may be difficult to understand or qualitatively justify to policy-makers.

When attempting to understand a complicated problem such as finding the optimal vaccination policy, it is often helpful to look at cases with extreme parameter values via asymptotic analysis, which helps the problem to be analytically solvable (at least to leading order). This can help from general principles for optimal vaccination policies. These principles can then be used both to form heuristics for finding the true optimal policy in a more general setting and also to explain the resultant optimal solution, as it is often comprised of a mixture of policies resulting from these principles.

There have been a number of recent papers that have used asymptotic analysis to derive general principles. Gavish and Katriel (2022) discusses a model with high reproduction numbers and shows that in this case, it is often optimal to vaccinate the less infectious groups in a population. Moreover, Rao and Brandeau (2021), building on the work of Zaric and Brandeau (2001), linearises the model equations and derives a simple knapsack problem, although the solution to this problem is only optimal when considering the short-term evolution of the epidemic. Other special cases are investigated in Duijzer et al. (2018) (which looks at a population with disconnected subgroups) and Duijzer et al. (2016) (which examines the critical vaccination fraction for a population with separable mixing).

Two cases will be considered in this paper, which both provide novel contributions to the literature. Firstly, the case of a population with a small vulnerable subgroup will be analysed, and it will be shown that, in the asymptotic limit (as the size of this population group tends to zero and its vulnerability tends to infinity), any vaccination policy is eventually outperformed by one where this group is vaccinated first. Of course, the concept that vaccinating vulnerable groups is important has been raised in many previous papers, such as Moore et al. (2021) and Dushoff et al. (2007), but the mathematically rigorous asymptotics presented here provide new evidence for the importance of this principle.

The second case to be discussed is that of a small total vaccination supply. The key novel result that will be shown is that (to leading order) the optimal vaccination problem reduces to a linear knapsack problem, which can be easily solved. This knapsack problem differs from the one in Rao and Brandeau (2021) because, by linearising the final size equations rather than the model ODEs (ordinary differential equations), the optimal solutions and predictions of their behaviour are valid for the full evolution of the epidemic, rather than just in the short term. Again, the case of a small vaccine supply has been examined in many papers such as Shim (2021, 2011) and Medlock and Meyers (2009), but these papers have simply analysed the optimisation problem in the standard way, without deriving the explicit leading-order solution as is done in this paper.

In order to prove these results, it is necessary to build on previous literature. A number of results from Penn and Donnelly (2022) (found in Appendix D) are used in the course of the proof alongside some well-established results, such as the final size of an epidemic in SIR-type models (Anderson and May 1992). However, the theorems presented in the main text are completely novel, with their proofs requiring a significant extension of the current literature. In particular, the various propositions in the proofs (found in Appendices A–C) are, to the best of the authors’ knowledge, new to the literature. Some of these results, such as, for example, the proof that epidemic final size is continuously dependent on initial conditions and the vaccination policy found in Proposition 5 may also be helpful to those seeking to prove similar results.

The main analytic results will be further investigated through examples, and, in particular, the small supply case will be used to show that it is not always optimal to vaccinate the most infectious group, even when all groups are equally vulnerable. The UK population’s age structure will be used to relate these results to a realistic example, and optimal small-supply vaccination policies will be approximated for diseases with different age-dependent case fatality ratios.

The paper is structured as follows: Firstly, the multi-group SIR model will be introduced. Then, analytic results will be presented in the case of a small vulnerable subgroup, which will be explored through numerical examples. Finally, analytic results related to a small vaccination supply will be presented and again, examples will be used to illustrate the findings.

## Modelling

### Disease Transmission and Vaccination Model

The model used in this paper is identical to the model presented in Penn and Donnelly (2022), and this section is simply a summary of the modelling section in Penn and Donnelly (2022). The population is divided into *n* subgroups, and each subgroup *i* is further divided into six compartments:

1 $$\begin{aligned} S_i :=&\text {Number of people that are in group}\ i, \text {are susceptible, and are unvaccinated} \end{aligned}$$

2 $$\begin{aligned} I_i:=&\text {Number of people that are in group}\ i, \text {are currently infected, and}\nonumber \\&\text {were infected while unvaccinated }\end{aligned}$$

3 $$\begin{aligned} R_i:=&\text {Number of people that are in group}\ i, \text {are recovered, and}\nonumber \\&\text {were infected while unvaccinated} \end{aligned}$$

4 $$\begin{aligned} S^V_i :=&\text {Number of people that are in group}\ i, \text {are susceptible and are vaccinated} \end{aligned}$$

5 $$\begin{aligned} I^V_i :=&\text {Number of people that are in group}\ i, \text {are infected}\nonumber \\&\text {and were infected after being vaccinated}\end{aligned}$$

6 $$\begin{aligned} R^V_i :=&\text {Number of people that are in group}\ i, \text {are recovered and were infected} \nonumber \\&\text {after being vaccinated.} \end{aligned}$$

Using SIR principles, the model becomes

7 $$\begin{aligned} \frac{{\textrm{d}}S_i}{{\textrm{d}}t}&= -\sum _{j=1}^n(\beta ^1_{ij}I_j+ \beta ^2_{ij} I^V_j)S_i - \frac{U_i(t) S_i}{N_i-W_i(t)} \end{aligned}$$

8 $$\begin{aligned} \frac{{\textrm{d}}I_i}{{\textrm{d}}t}&= \sum _{j=1}^n(\beta ^1_{ij} I_j+ \beta ^2_{ij} I^V_j)S_i - \mu ^1_i I_i\end{aligned}$$

9 $$\begin{aligned} \frac{{\textrm{d}}R_i}{{\textrm{d}}t}&= \mu ^1_i I_i\end{aligned}$$

10 $$\begin{aligned} \frac{{\textrm{d}}S^V_i}{{\textrm{d}}t}&= -\sum _{j=1}^n(\beta ^3_{ij}I_j + \beta _{ij}^4 I^V_j)S^V_i + \frac{U_i(t) S_i}{N_i-W_i(t)}\end{aligned}$$

11 $$\begin{aligned} \frac{{\textrm{d}}I^V_i}{{\textrm{d}}t}&= \sum _{j=1}^n(\beta ^3_{ij}I_j + \beta _{ij}^4I^V_j)S^V_i -\mu ^2_i I^V_i \end{aligned}$$

12 $$\begin{aligned} \frac{{\textrm{d}}R^V_i}{{\textrm{d}}t}&= \mu ^2_i I^V_i \end{aligned}$$

where

13 $$\begin{aligned} W_i(t) := \int _0^t U_i(s){\textrm{d}}s, \end{aligned}$$

and

14 $$\begin{aligned} N_i = S_i(t) + I_i(t) + R_i(t) + S^V_i(t) + I^V_i(t) + R^V_i(t) \end{aligned}$$

is the size of group *i*. Moreover, the $\beta ^{\alpha }_{ij}$ terms represent transmission from group *j* to group *i* and the $\mu _i^{\alpha }$ terms give the infectious period of the relevant individuals in group *i*.

Here, $U_i(t){\textrm{d}}t$ gives the number of individuals in group *i* that are vaccinated in the small time interval $[t,t+{\textrm{d}}t]$, and hence, $W_i(t)$ is the number of individuals that have been vaccinated in group *i* in [0, *t*]. It is assumed that these vaccinations are assigned randomly to the unvaccinated members of group *i*, so that each vaccine is given to a susceptible member of group *i* with probability

15 $$\begin{aligned} \frac{\text {number of susceptible members}}{\text {number of unvaccinated members}} = \frac{S_i}{N_i - W_i(t)} \end{aligned}$$

Thus, the total rate of susceptibles being vaccinated is $\frac{U_i(t) S_i}{N_i-W_i(t)}$.

Note that there is a slight difference between this model and the one commonly found in the literature (in Hansen and Day 2011; Zaman et al. 2008; Kar and Batabyal 2011 among many others) which set the vaccination term equal to $S_iU_i(t)$ instead of $\frac{U_i(t) S_i}{N_i-W_i(t)}$. As discussed in Penn and Donnelly (2022), this corresponds to vaccines that are randomly distributed to the whole population, which can be seen by rewriting the vaccination term as:

16 $$\begin{aligned} S_iU_i(t){\textrm{d}}t = \frac{S_i}{N_i} \times N_iU_i(t){\textrm{d}}t \end{aligned}$$

The first term on the right-hand side is then the probability of a randomly chosen member of group *i* being susceptible, while the second term is the total number of vaccines assigned in a small time interval $[t,t+{\textrm{d}}t]$, noting that here the dimension of $U_i(t)$ is 1/time (compared to the model used in this paper where the dimension of $U_i(t)$ is population/time), and hence, it is necessary to scale by $N_i{\textrm{d}}t$ to convert $U_i(t)$ into a number of vaccines.

This is in contrast to the model in this paper which corresponds to vaccines that are randomly distributed only to the unvaccinated population. Penn and Donnelly (2022) provides justification for the use of this “unvaccinated-only model”, which is therefore the one that will be used in this paper. However, they are structurally very similar, and so it would be possible to apply the results in this paper to the more commonly found model.

To deal with the (removable) singularity that can occur when $W_i = N_i$, it is assumed that

17 $$\begin{aligned} W_i(t)\le N_i \quad \forall t \ge 0 \quad \text {and} \quad W_i(t) = N_i \Rightarrow \frac{U_i(t) S_i}{N_i-W_i(t)} = 0 \end{aligned}$$

To capture the benefits of vaccination, there are additional constraints put on the $\beta _{ij}^{\alpha }$ and $\mu _j^{\alpha }$ terms which are

18 $$\begin{aligned} \beta ^1_{ij} \ge \beta _{ij}^2, \beta _{ij}^3 \ge \beta _{ij}^4 \quad \text {and} \quad \mu _i^1 \le \mu _i^2. \end{aligned}$$

Finally, it will be assumed throughout the remainder of this paper that the population sizes are normalised so that

19 $$\begin{aligned} \sum _{i=1}^n N_i = 1 \end{aligned}$$

Further details are given in Penn and Donnelly (2022).

### Optimisation Problem

The optimal vaccination problem considered in this paper aims to find the vaccination policy, ${\varvec{U}}$, which minimises a weighted sum of the total number of infections in each group. Thus, the problem is:

20 $$\begin{aligned} \min \bigg \{\sum _{i=1}^np_i\bigg (R_i(\infty ) + \kappa _i R^V_i(\infty )\bigg ) :&\sum _{i=1}^nU_i(t) \le A(t),\quad \sum _{i=1}^n W_i(t) \le B(t),\nonumber \\&U_i(t)\ge 0, \quad W_i(t)\le N_i \quad \forall t \ge 0\bigg \}. \end{aligned}$$

Here, *A*(*t*) represents the maximal vaccination rate, *B*(*t*) represents the maximal vaccine supply and $R_i(\infty )$ and $R_i^V(\infty )$ are the limiting values of $R_i(t)$ and $R_i^V(t)$ as $t \rightarrow \infty$. The weights $p_i$ and $p_i\kappa _i$ could be interpreted in a number of ways, depending on the quantity of interest. For example, $p_i = \kappa _i = 1$ if one wanted to minimise infections, or $p_i$ and $p_i\kappa _i$ could be the case fatality ratio of unvaccinated and vaccinated members of group *i*, respectively, if one wanted to minimise deaths. However, it is important to note that $\kappa _i \le 1$ for each *i* as vaccinated members of the population should be no more vulnerable to the disease that their unvaccinated counterparts.

It is helpful to define $H({\varvec{U}})$ to be the objective function—that is

21 $$\begin{aligned} H({\varvec{U}}) = \sum _{i=1}^np_i\bigg (R_i(\infty ) + \kappa _i R^V_i(\infty )\bigg ), \end{aligned}$$

where $R_i$ and $R^V_i$ are found from solving the model equations with vaccination policy given by ${\varvec{U}}$.

It will be assumed throughout this paper that all “feasible” ${\varvec{U}}$ are sufficiently smooth for all the quoted theorems to hold. In general, this does not significantly restrict ${\varvec{U}}$—for example, the results in Penn and Donnelly (2022) simply require that each $U_i(t)$ is bounded and Lebesgue integrable, while Theorems 1 and 2 require only that ${\varvec{U}}$ has finite support. Moreover, it is assumed that *B*(*t*) is non-decreasing (as total supply should not decrease over time) and piecewise differentiable.

## Results

### A Small, Vulnerable Subgroup

Consider the case where one of the groups in the population (which, without loss of generality, will be assumed to be group 1) is very small and vulnerable. That is, the population $N_1$ satisfies

22 $$\begin{aligned} N_1(\epsilon ) =\epsilon<< 1 \end{aligned}$$

while the weights satisfy

23 $$\begin{aligned} p_1(\epsilon ) = p_1 \quad \text {and} \quad p_i(\epsilon ) = p^*_i\epsilon \quad \forall i \ne 1 \end{aligned}$$

for some constants $p_1$ and $p_i^*$. It will be assumed that all $\kappa _i$ are constant. In this setting, group 1 contains a very small proportion of the population, but each member of group 1 is much more vulnerable than the rest of the population.

Thus, this case is practically valid when there is a small subsection of the population that carries the majority of the vulnerability to a disease. As will be discussed further in Section 3.2.3, this has applicability to diseases such as COVID-19, where the majority of the deaths occur significantly older people, while it could also apply to diseases where there are rare conditions that cause a minority of people to be much more vulnerable.

It is mathematically convenient to rescale the parameters $p_i$ so that only $p_1$ depends on $\epsilon$. This can be done by multiplying all the $p_i$ terms by $\frac{1}{p_1\epsilon }$ so that

24 $$\begin{aligned} {\tilde{p}}_1(\epsilon ) = \frac{1}{\epsilon } \quad \text {and} \quad {\tilde{p}}_i(\epsilon ) = \frac{p^*_i}{p_1} := {\tilde{p}}_i \quad \forall i \ne 1. \end{aligned}$$

This leads to an equivalent optimisation problem in the sense that the optimal vaccination policy will be the same. This occurs because the only change to the objective function is a scalar multiplication of $\frac{1}{p_1\epsilon }$ to each of the terms. Note that while this multiplicative factor tends to infinity as $\epsilon$ tends to 0, this system is only analysed for nonzero values of $\epsilon$, and hence, this rescaling is valid.

#### Analytic Results

The first result presented in this section shows that, in the limit of a group with small size and large vulnerability (with the total cost of the whole group being infected, $N_1{\tilde{p}}_1$, remaining constant) any fixed vaccination policy where the vulnerable group is not vaccinated first will eventually (that is, for sufficiently small $\epsilon$) be outperformed by a similar policy where the vulnerable group is vaccinated first.

Group 1 will be given a population size $N_1 = \epsilon$ and an infection cost ${\tilde{p}}_1 = \frac{1}{\epsilon }$ (recall that the ${\tilde{p}}_i$ represent the rescaled values of $p_i$, and so it is acceptable that ${\tilde{p}}_1 > 1$ for small $\epsilon$). It will be assumed that the initial conditions in the group are proportional to $\epsilon$, so that there exists some $\sigma \in (0,1]$ such that the initial susceptible population is $\sigma \epsilon$ and the initial infected population is $(1-\sigma )\epsilon$.

Before stating the full theorem, it is helpful to explain the various constraints and variables that will be introduced. Define, for each value of $\epsilon \ge 0$, ${\varvec{U}}(t;\epsilon )$ to be the “fixed” vaccination policy where group 1 is not vaccinated first. Of course, the vaccination policy cannot be completely fixed, as the size, $\epsilon$, of group 1 is decreasing, and so it will simply be assumed that the vaccines given out to each group satisfies

25 $$\begin{aligned} |W_i(t;\epsilon ) - W_i(t;0) |< \epsilon \quad \forall t \ge 0 \quad \text {and} \quad \forall i \in \{1,\ldots ,n\} \end{aligned}$$

Note that all groups are allowed to have small changes in the number of vaccinations they receive—this allows, for example, for vaccinations that would have been given to group 1 being reassigned as group 1’s population shrinks.

Moreover, to reduce the lengths of the proofs, it will be assumed that ${\varvec{U}}$ has uniformly bounded finite support—that is, there is some constant $t_U$ such that for each $i \in \{1,\ldots ,n\}$,

26 $$\begin{aligned} t > t_U \Rightarrow U_i(t;\epsilon ) = 0 \quad \forall t,\epsilon \ge 0 \end{aligned}$$

In order for group 1 to not be vaccinated first in the limit as $\epsilon \rightarrow 0$, there must be some time $\tau$ at which some fixed proportion *w* of the other groups have been vaccinated, while at least some fixed proportion $(1-\alpha )$ of group 1 has not been vaccinated. That is,

27 $$\begin{aligned} W_1(\tau ;\epsilon ) < \alpha \epsilon \quad \text {and} \quad \sum _{i=1}^n W_i(\tau ;\epsilon ) > w. \end{aligned}$$

One can also define a vaccination policy $\tilde{{\varvec{U}}}(t;\epsilon )$ where group 1 is vaccinated first. This will be done by re-directing all vaccinations from the ${\varvec{U}}(t;\epsilon )$ policy to group 1 until it is fully vaccinated, and keeping the same vaccination policy after group 1 is fully vaccinated (ignoring any vaccines that ${\varvec{U}}(t;\epsilon )$ assigns to group 1 after this time).

To ensure convergence of the model at $\epsilon = 0$, given $\Pi (\epsilon )$ defined by

28 $$\begin{aligned} \Pi (\epsilon ) := \bigg \{ i : \exists t \ge 0 \quad \text {s.t.} \quad I_i(t;\epsilon ) > 0\bigg \}, \end{aligned}$$

it will be assumed that $\Pi (\epsilon ) = \{1,\ldots ,n\}$ for all $\epsilon > 0$ (as any groups which never suffer any infections can be ignored) and that $\Pi (0) = \{2,\ldots ,n\}$. While this second condition may not be strictly necessary for the theorem to hold, it is unrestrictive and ensures convergence—if this were not the case, then it would be possible that infection in some set of groups were seeded only by group 1. Thus, when $\epsilon = 0$, these groups would suffer no infections, while for any $\epsilon > 0$, they would have an epidemic of size independent (at leading order) of $\epsilon$.

The final condition on the model is that the people in group 1 can be infected by other groups and that vaccinated members of group 1 gain protection from this infection. That is, there is some $i \in \{1,\ldots ,n\}$ such that

29 $$\begin{aligned} \beta ^1_{1i} > \beta _{1i}^3 \ge 0. \end{aligned}$$

This is an important condition, as if people group 1 could only be infected by other members of group 1 the total number of infections in group 1 would decay as $\epsilon \rightarrow 0$, meaning that it would no longer necessarily be optimal to vaccinate group 1 first (as most people in group 1 would not catch the disease anyway for small $\epsilon$).

With these considerations, Theorem 1 can now be stated.

##### Theorem 1

Suppose that for all $\epsilon > 0$,

30 $$\begin{aligned} N_1(\epsilon ) = \epsilon , \quad S_1(0;\epsilon ) = \epsilon \sigma , \quad I_1(0;\epsilon ) = (1-\sigma )\epsilon \quad \text {and} \quad {\tilde{p}}_1(\epsilon ) = \frac{1}{\epsilon } \end{aligned}$$

for some $\sigma \in (0,1)$ and that all other parameter values and initial conditions are independent of $\epsilon$.

Consider any vaccination policy with uniformly bounded finite support given by ${\varvec{U}}(t;\epsilon )$ and suppose that there exists fixed $\alpha ,\tau , w > 0$ such that

31 $$\begin{aligned} W_1(\tau ;\epsilon ) < \alpha \epsilon \quad \text {and} \quad \sum _{i=1}^n W_i(\tau ;\epsilon )> w \quad \forall \epsilon >0. \end{aligned}$$

Define a new policy, $\tilde{{\varvec{U}}}(t;\epsilon )$, given by

32 $$\begin{aligned} {\tilde{U}}_1(t;\epsilon ) = \left\{ \begin{matrix} \sum \nolimits _{i=1}^n U_i(t) &{} \text {if } \sum \nolimits _{i=1}^n W_i(t;\epsilon ) \le \epsilon \\ 0 &{} \text {otherwise} \\ \end{matrix} \right. \end{aligned}$$

and, for $$i \ne 1$$,

33 $$\begin{aligned} {\tilde{U}}_i(t;\epsilon ) = \left\{ \begin{matrix}0 &{} \text {if } \sum \nolimits _{i=1}^n W_i(t;\epsilon ) \le \epsilon \\ U_i(t;\epsilon ) &{} \text {otherwise} \\ \end{matrix} \right. . \end{aligned}$$

Suppose that for each $i \in \{1,\ldots ,n\}$ and $t \ge 0$,

34 $$\begin{aligned} |W_i(t;0) -W_i(t;\epsilon ) |< \epsilon . \end{aligned}$$

Define

35 $$\begin{aligned} \Pi (\epsilon ) := \{ i : \exists t \ge 0 \quad \text {s.t.} \quad I_i(t;\epsilon ) > 0\} \end{aligned}$$

and suppose that $\Pi (\epsilon ) = \{1,\ldots ,n\}$ for any $\epsilon > 0$ and that $\Pi (0) = \{2,\ldots ,n\}$. Finally, suppose that there exists an $i \in \{2,\ldots ,n\}$ such that

36 $$\begin{aligned} \beta _{1i}^1>\beta _{1i}^3 \ge 0. \end{aligned}$$

Then, the policy $\tilde{{\varvec{U}}}$ is feasible and for sufficiently small $\epsilon$,

37 $$\begin{aligned} H({\varvec{U}}(t;\epsilon )) > H(\tilde{{\varvec{U}}}(t;\epsilon )). \end{aligned}$$

For the second theorem, it is helpful to note that, using the results in Penn and Donnelly (2022), if one defines

38 $$\begin{aligned} \chi (t) := \left\{ \begin{matrix} A(t) &{} \text {if} \quad \int _0^tA(s){\textrm{d}}s < B(t) \\ \min (A(t), B'(t)) &{} \text {if} \quad \int _0^tA(s){\textrm{d}}s \ge B(t) \end{matrix}\right. , \end{aligned}$$

then (assuming that there is an optimal solution, and under mild smoothness conditions on ${\varvec{U}}$, *A* and *B*) there must be an optimal solution satisfying

39 $$\begin{aligned} \sum _{i=1}^nW_i(t) =\max \bigg (\int _0^t \chi (s){\textrm{d}}s,1\bigg ). \end{aligned}$$

The following theorem then proves that the limiting optimal vaccination policy vaccinates the vulnerable group as quickly as possible. To reduce the length of the proof, it will be assumed that $\sigma = 1$, so that (in the small $\epsilon$ limit) all members of group 1 can be vaccinated before being infected.

##### Theorem 2

With the definitions of Theorem 1, suppose additionally that

40 $$\begin{aligned} \sum _{j=2}^n (\beta _{1j}^1 - \beta _{1j}^3)I_j(0;\epsilon ) > 0. \end{aligned}$$

That is, the initial difference between the infective force on vaccinated and unvaccinated members of the population is positive. Suppose further that

41 $$\begin{aligned} \sigma = 1. \end{aligned}$$

Suppose an optimal vaccination policy for each $\epsilon$ is given by $\overline{{\varvec{U}}}(t;\epsilon )$ and suppose that $\overline{{\varvec{U}}}(t;\epsilon )$ has uniformly bounded finite support. Then, there exists an $\eta$ depending only on $\alpha$, $\tau$, *w* and the model parameters such that, for any ${\varvec{U}}$ satisfying the condition (31) as defined in Theorem 1

42 $$\begin{aligned} \epsilon \in (0,\eta ) \Rightarrow H({\varvec{U}}) > H(\overline{{\varvec{U}}}). \end{aligned}$$

Moreover, there is a sequence of optimal vaccination policies, $\overline{{\varvec{U}}}(t;\epsilon )$, which satisfies

43 $$\begin{aligned} \lim _{\epsilon \rightarrow 0}\left( \frac{{\overline{W}}_1(t;\epsilon )}{\epsilon }\right) = 1 \quad \forall t \quad \text {s.t.}\quad \int _0^t \chi (s){\textrm{d}}s > 0. \end{aligned}$$

Note that the existence of an optimal vaccination policy has been assumed in the statement of this theorem. The authors believe that an optimal policy should exist, as Proposition 5 in the appendices can be used to show that $H({\varvec{U}})$ is continuous. However, more care would need to be taken with the smoothness assumptions on ${\varvec{U}}$ to create a rigorous proof of this.

Theorems 1 and 2 are proved in the appendices.

#### Examples

To illustrate these analytic results, consider a simple two-group example. Suppose that group 1 is small, vulnerable, and non-infectious, while group 2 is large, invulnerable and infectious. These groups could be interpreted as “old” and “young”, respectively, although there is no specific physical situation being modelled here.

Suppose the transmission matrices are given by

44 $$\begin{aligned} \beta ^{1}&= \begin{pmatrix} 1 &{} 2 \\ 2 &{} 4 \\ \end{pmatrix}, \quad \beta ^{2} = \chi \beta ^1 \quad \beta ^{3} = \rho \beta ^1 \quad \text {and} \quad \beta ^{4} = \chi \rho \beta ^1 \end{aligned}$$

for some parameters $\chi$ and $\rho$ which will be varied. This corresponds to the case of vaccination having (independently) an effectiveness $\chi$ at stopping people being infected and $\rho$ at stopping infected people transmitting the disease. Moreover, suppose that

45 $$\begin{aligned} \mu _i^{\alpha } = 1 \quad \forall i, \alpha \end{aligned}$$

and

46 $$\begin{aligned} N_1 = \epsilon , \quad {\tilde{p}}_1 = \frac{1}{\epsilon },\quad \kappa _1 = 1 \quad N_2 = 1, \quad {\tilde{p}}_2 = p^* \quad \text {and} \quad \kappa _2 = 1, \end{aligned}$$

for some parameter $p^*$ that will be varied. Finally, suppose that the initial conditions are

47 $$\begin{aligned} S_1(0;\epsilon ) = \epsilon , \quad I_1(0;\epsilon ), = 0 \quad S_2(0;\epsilon ) = 1-I^* \quad \text {and} \quad I_2(0;\epsilon ) = I^*, \end{aligned}$$

for some parameter $I^*$ that will be varied and that the vaccination constraints are given by:

48 $$\begin{aligned} A(t) = 1\quad \text {and} \quad B(t) = \max (t,1). \end{aligned}$$

Consider therefore a vaccination policy where group 2, the infectious group, is vaccinated first (and hence, as $B(\infty ) = N_2$, it is the only group that is vaccinated). That is,

49 $$\begin{aligned} U_1(t;\epsilon ) = 0 \quad \text {and} \quad U_2(t;\epsilon ) =\left\{ \begin{matrix} 1 &{} \text {if } t \le 1\\ 0 &{} \text {otherwise} \\ \end{matrix}\right. . \end{aligned}$$

Hence, with $\tilde{{\varvec{U}}}$ defined as in Theorem 1, one has

50 $$\begin{aligned} {\tilde{U}}_1(t;\epsilon ) = \left\{ \begin{matrix} 1 &{} \text {if } t \le \min (1,\epsilon )\\ 0 &{} \text {otherwise} \\ \end{matrix}\right. \quad \text {and} \quad {\tilde{U}}_2(t;\epsilon ) = \left\{ \begin{matrix} 1 &{} \text {if } t \in (\epsilon ,1]\\ 0 &{} \text {otherwise} \\ \end{matrix}\right. . \end{aligned}$$

Figure  shows a comparison of the objective values $H({\varvec{U}}(t;\epsilon ))$ and $H(\tilde{{\varvec{U}}}(t;\epsilon ))$ for different values of $\epsilon$. As expected, when $\epsilon = 1$, vaccinating the more infectious group first is optimal (as they have the same vulnerability in this case), while for $\epsilon$ smaller than around 0.1, it becomes more effective to vaccinate the vulnerable group first, illustrating the results of Theorem 1.

**Fig. 1:**
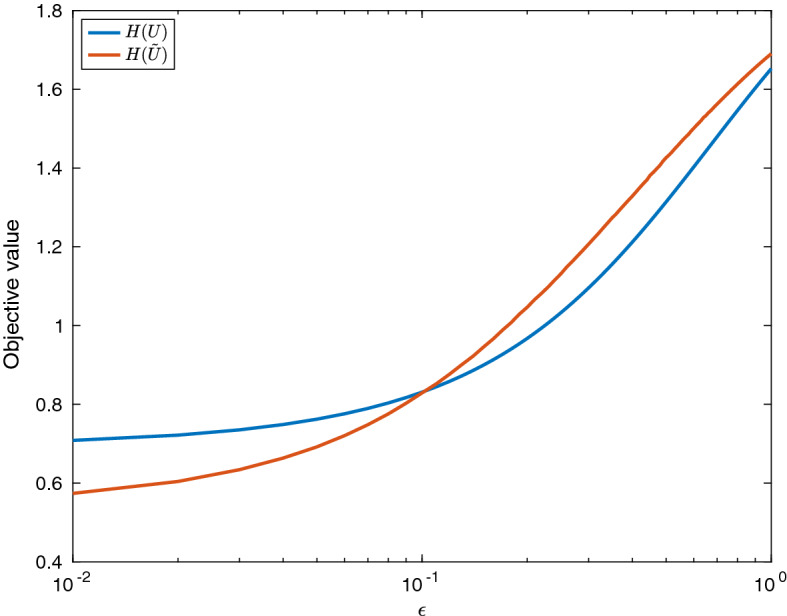
(Color Figure Online) A comparison of the two vaccination policies, ${\varvec{U}}(t;\epsilon )$ (where the infectious group is vaccinated first) and $\tilde{{\varvec{U}}}(t;\epsilon )$ (where the vulnerable group is vaccinated first) for different values of $\epsilon$. Note that here, $I^* = 0.01$, $\chi = \rho = 0.5$ and $p^* = 1$

It is useful to consider the approximate smallness of $\epsilon$ required in Theorem 1. That is, how small $\epsilon$ needs to be in order for $\tilde{{\varvec{U}}}(t;\epsilon )$ to be the better vaccination policy. To explore this, define, for each value of $I^*$ and $p^*$,

51 $$\begin{aligned} \epsilon ^*(I^*,p^*) := \inf \bigg (\left\{ \epsilon : H(\tilde{{\varvec{U}}}(t;\epsilon )) > H({\varvec{U}}(t;\epsilon ))\right\} \cup \{1\}\bigg ). \end{aligned}$$

That is, $\epsilon ^*(I^*,p^*)$ is the smallest value of $\epsilon$ such that vaccinating group 2 first is better that the $\tilde{{\varvec{U}}}$ policy, with a cut-off value at 1 (as it is possible that for some parameter values, the $\tilde{{\varvec{U}}}$ policy is always better).

Figure  shows the behaviour of $\epsilon ^*(I^*,p^*)$. As expected, $\epsilon ^*$ is decreasing in $I^*$—this is because when there are fewer initial infectives, there is more time to vaccinate the infectious group before the epidemic has a chance to grow, reducing the peak of the epidemic. Moreover, $\epsilon ^*$ is decreasing in $p^*$, as higher values of $p^*$ mean that the number of infections in group 2 is valued higher.

**Fig. 2:**
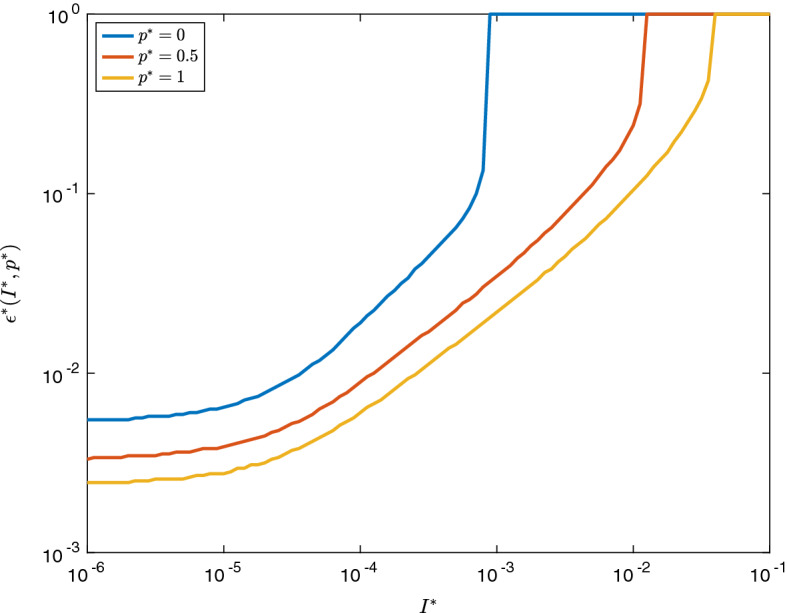
(Color Figure Online) A plot of $\epsilon ^*(I^*,p^*)$, the highest value of $\epsilon$ for which ${\varvec{U}}$, is a better vaccination policy that $\tilde{{\varvec{U}}}$. Note that $\epsilon ^*$ is capped at 1, so that a value of 1 indicates that there were no values found of $\epsilon ^*$ such that ${\varvec{U}}$ was the better policy. Note that here, $\chi = \rho = 0.5$

Moreover, Fig. 2 suggests that, for each fixed $p^*$, $\epsilon ^*$ is uniformly bounded below for all $I^*$. Indeed, this is expected as when $I^*$ is very small, there are negligible infections within the interval $t \in [0,1]$ and so the vaccination policies ${\varvec{U}}$ and $\tilde{{\varvec{U}}}$ are in effect being carried out in a completely uninfected population. As the $R_0$ (that is, the initial growth rate of the disease) number of a fully vaccinated population (in this case) is greater than 1, $I(t;\epsilon )$ will reach an *O*(1) value regardless of the vaccination policy. Thus, while decreasing $I^*$ will increase the time to reach this *O*(1) value, it will not significantly change the final infections in the epidemic, and hence, $\epsilon ^*$ should converge to a fixed value for small $I^*$.

When the fully vaccinated population has an $R_0$ lower than 1, the difference between ${\varvec{U}}$ and $\tilde{{\varvec{U}}}$ is more distinct. Indeed, provided $I^*$ is small enough for vaccination to be completed before many infections have occurred, one would expect $O(I^*)$ infections in group 2 in either of the two vaccination policies (for sufficiently small $\epsilon$), as in both policies, the size of the infected compartment will be decreasing after the vaccination has been completed. However, in the ${\varvec{U}}$ case, one would expect $O(I^* \epsilon )$ infections in total in group 1 (as there is an $O(I^*)$ infection force on a group of size $O(\epsilon )$ for *O*(1) time), while in the $\varvec{{\tilde{U}}}$ case, one would expect $O(I^*\epsilon ^2)$ infections in total in group 1, as the population of this group is only of size $O(\epsilon )$ for $O(\epsilon )$ time. This behaviour is illustrated in Fig. , which shows that $\epsilon ^*$ converges to significantly higher values than in Fig. 2—indeed, in the case that $p^* = 0$, it appears that ${\varvec{U}}$ is never optimal for any $\epsilon \le 1$.

**Fig. 3:**
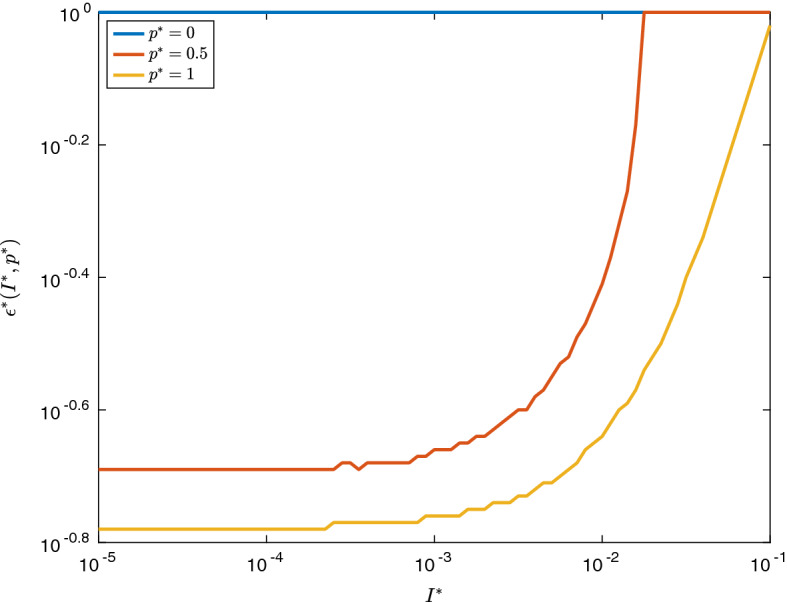
(Color Figure Online) A plot of $\epsilon ^*(I^*,p^*)$, the highest value of $\epsilon$ for which ${\varvec{U}}$ is a better vaccination policy that $\tilde{{\varvec{U}}}$, in the case of complete vaccination effectiveness (so $\chi = \rho = 0$). Note that because the values of the objective function are $O(I^*)$, there is some numerical instability which has caused some non-smoothness of the plot

### A Small Vaccination Supply

In this section, the case of a small, immediately available vaccine supply will be considered. In this case, it will be possible to analytically derive the optimal vaccination policy (in the limit of small supply).

This case may be particularly relevant if there was an outbreak of a disease where a vaccine already existed (so that some vaccinations are available immediately), but where supplies were limited, and scaling production would take a significant amount of time. An example of this can be found in the recent monkeypox outbreak (Mahase 2022) where the UK initially purchased 20 000 smallpox vaccines. This small figure—not even enough to vaccinate 0.1% of the UK population (UN 2019)—would certainly fall within the small vaccination supply case.

Moreover, one can use the results in this section regardless of the time at which vaccinations become available (that is, they are not only relevant at the start of an epidemic). This would be of practical use whenever vaccine production is slow, or when the disease is sufficiently mild (or vaccine production is sufficiently expensive) that a large-scale vaccination program is not deemed economically feasible.

#### Analytic Results

To state the analytic result from this section, it is helpful to define

52 $$\begin{aligned} \beta '_{ij} = \left\{ \begin{matrix} \beta ^1_{ij} &{} \text {if } i,j\le n\\ \beta ^2_{i(n-j)} &{} \text {if } i \le n< j \le 2n\\ \beta ^3_{(n-i)j} &{} \text {if } j \le n< i\le 2n \\ \beta ^4_{(n-i)(n-j)} &{} \text {if } n < i,j \le 2n\\ \end{matrix}\right. ,. \end{aligned}$$

This large transmission matrix captures the dynamics of all 2*n* susceptible and infectious groups in the model (both vaccinated and unvaccinated). Indeed, after vaccination has been completed, there is no movement from $S_i$ to $S^V_i$ so $\beta '$ allows for the model to be considered as a 2n-group SIR model without vaccination. Thus, in particular, one can derive a simple final size relation for the total number of infections in the epidemic. Similarly, define

53 $$\begin{aligned} \mu '_i = \left\{ \begin{matrix} \mu _i^1 &{} \text {if } i \le n\\ \mu _{(i-n)}^2 &{} \text {if } n < i \le 2n \end{matrix}\right. \end{aligned}$$

and

54 $$\begin{aligned} p'_i = \left\{ \begin{matrix} p_i &{} \text {if } i \le n\\ \kappa _{(i-n)} p_{(i-n)} &{} \text {if } n<i\le 2n\end{matrix}\right. . \end{aligned}$$

In this case of small supply, it is possible to effectively differentiate the final size of the epidemic with respect to the vaccination policy and use the resultant linear approximation to form a simple knapsack problem for the optimal vaccination policy. This will involve writing the objective in the form:

55 $$\begin{aligned} H({\varvec{U}}(t;\epsilon )) = H({\varvec{0}}) + {\varvec{y}}^T{\varvec{W}}(\tau (\epsilon );\epsilon ) + o(\epsilon ) \end{aligned}$$

where ${\varvec{W}}$ is the final vaccination amounts in each group. To define the gradient, ${\varvec{y}}$, it is necessary to use the inverse of a matrix ${\varvec{Q}}$ given by

56 $$\begin{aligned} Q_{ij} = \frac{1}{1-e^{-\sum \nolimits _{j=1}^{2n}\frac{\beta '_{ij}}{\mu '_j}R_j(\infty ;0)}}\bigg [\delta _{ij} +\frac{ S_i(0;0)e^{-\sum \nolimits _{j=1}^{2n}\frac{\beta '_{ij}}{\mu '_j}R_j(\infty ;0)}\beta '_{ij}}{\mu '_j}\bigg ], \end{aligned}$$

where as before, the variables $f_i(t;\eta )$ indicate the value of the relevant model variable at time *t*, given that the parameter $\epsilon$ is equal to $\eta$, and $\delta _{ij}$ is the Kronecker delta. Then, ${\varvec{y}}$ is defined by:

57 $$\begin{aligned} {\varvec{x}} = {\varvec{Q}}^{-T}{\varvec{p}}' \quad \text {and} \quad y_i = \frac{S_i(0;0)}{N_i}(x_{i+n} - x_i) \quad \forall i \in \{1,\ldots ,n\} . \end{aligned}$$

These definitions allow for the theorem to be stated.

##### Theorem 3

Suppose that, for all $\epsilon > 0$

58 $$\begin{aligned} B(t;\epsilon ) = \epsilon \quad \forall t \ge 0. \end{aligned}$$

and that all other parameter values and initial conditions are independent of $\epsilon$. Suppose that *A*(*t*) is a continuous function with

59 $$\begin{aligned} A(0) > 0 \end{aligned}$$

and that the matrix *M* is invertible. For sufficiently small $\epsilon$, define

60 $$\begin{aligned} \tau (\epsilon ) := \inf \bigg \{t : \int _0^t A(s){\textrm{d}}s = \epsilon \bigg \}. \end{aligned}$$

Suppose that ${\varvec{U}}$ satisfies the condition

61 $$\begin{aligned} \sum _{i=1}^nU_i(s) = \min \bigg (\int _0^t\chi (s){\textrm{d}}s,1\bigg ), \end{aligned}$$

where $\chi$ is defined in (B169). Then, for sufficiently small $\epsilon$, the objective function is given by:

62 $$\begin{aligned} H({\varvec{U}}(t;\epsilon )) = H({\varvec{0}}) + {\varvec{y}}^T{\varvec{W}}(\tau (\epsilon );\epsilon ) + o(\epsilon ). \end{aligned}$$

Moreover, if there is a unique element of ${\varvec{y}}$ equal to the minimum of ${\varvec{y}}$ then the optimal vaccination policy (to leading order in $\epsilon$) is uniquely given by:

63 $$\begin{aligned} U_i(t;\epsilon ) = \left\{ \begin{matrix} A(t) &{}\text {if } i = \min \{ y_i : i \in \{1,\ldots ,n\} \} &{} \text {and } \int _0^t A(s){\textrm{d}}s < \epsilon \\ 0 &{} \text {otherwise} \\ \end{matrix}\right. . \end{aligned}$$

The second part of the theorem assumes a unique minimal element of ${\varvec{y}}$. This is not guaranteed to happen, and if there were multiple groups with equal values of ${\varvec{y}}$, this would mean that the effectiveness of vaccinating these groups would be equal to $O(\epsilon )$. However, any sets of parameters satisfying this condition would be unstable to small perturbations (as a trivial example, consider perturbing the initial susceptible populations $S_i(0,0)$ of the groups with a minimal values of $y_i$). Thus, in any practical scenario, the probability that the best estimates of the parameters give multiple minimal values of $y_i$ is very small.

Theorem 3 is proved in the appendices.

#### Vaccinating a Homogeneous Population

To illustrate the effectiveness of this approximation, consider first an example of a homogeneous population (so $n=1$). Consider the case where $\beta ^1 = \beta$, $\beta ^2 = \beta ^3 = 0.5\beta$ and $\beta ^4 = 0.25 \beta$ for some parameter $\beta$ that will be varied. Suppose moreover that

64 $$\begin{aligned} N_1= \mu ^1_1 = \mu ^2_1 = p_1 = \kappa _1 = A(t) = 1, \quad S_1(0) = 1-10^{-4} \quad \text {and}\quad I_1(0) = 10^{-4}.\nonumber \\ \end{aligned}$$

Finally, suppose $B(t) = \epsilon$ where $\epsilon$ will be varied.

Figure  shows a comparison of the predicted and actual change in number of deaths, $\rho _1$ for two values of $\epsilon$. It illustrates that, even when $\epsilon = 0.1$, a relatively large value, ${\varvec{y}}$ gives a good approximation of the true value (found by simulation). Moreover, when $\epsilon = 0.01$, the two lines are almost indistinguishable. This is useful validation for the approximation, as the correction term was simply proved to be $o(\epsilon )$ rather than, for example, $O(\epsilon ^2)$, and so it is encouraging that the predictions are so close.

**Fig. 4:**
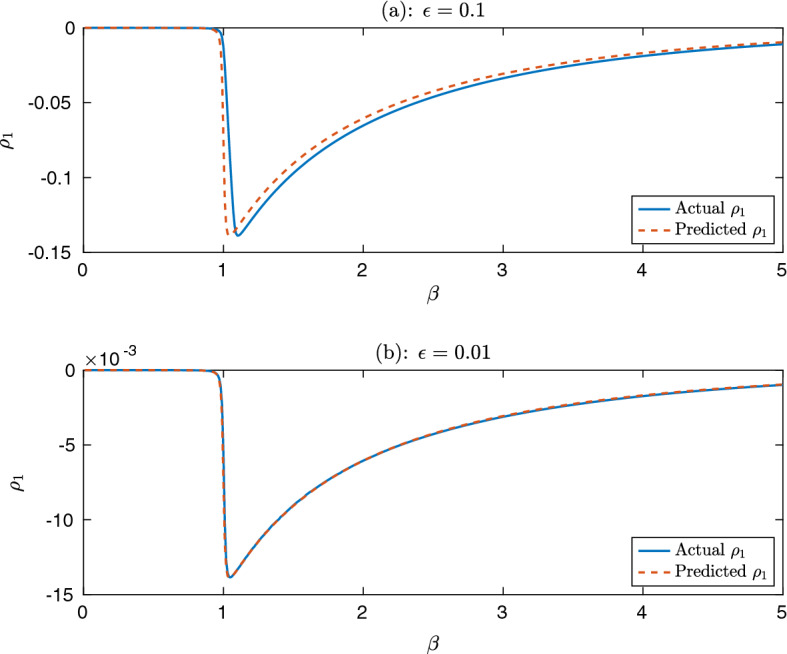
(Color Figure Online) A comparison of the predicted and actual values of the change in deaths, $\rho _1$, in the case of a homogeneous population for two different values of vaccination supply, $\epsilon$ and for different values of infectivity, $\beta$. Note the different scales on the two y axes

An interesting property of Fig. 4 is that the value of $\beta$ for which vaccination is most effective appears to be very close to $S(0)\beta = 1$ (as $S(0) \sim 1$). Note that here, as $\mu =1$, this is equal to the initial reproduction number of the disease. This has the perhaps surprising consequence that if one has a set of disconnected, equally vulnerable subgroups, a small vaccination supply should be assigned to a group with initial reproduction number close to 1, rather than giving it to the group with the highest value of $\beta$ (that is, the most group with the most infectious individuals). This result is in line with the findings of Gavish and Katriel (2022), which showed that vaccinating less infectious groups can be more effective, and is an important consideration for vaccination policy planning.

#### Application to Age-Structured Populations

Consider assigning a small quantity of vaccinations to an age-structured population, using the example of the UK. The disease model has been estimated using the inter-age-group contact matrices $\varvec{\Lambda }$ from Prem et al. (2017), alongside population estimates ${\varvec{N}}$ from UN (2019). As in Prem et al. (2017), this gives a transmission matrix of

65 $$\begin{aligned} \beta ^1_{ij} = \beta \frac{\Lambda _{ij}}{N_j} \end{aligned}$$

for some scalar parameter $\beta$. As in the previous section, it will be assumed that

66 $$\begin{aligned} \mu _i^{\alpha } = 1 \quad \forall i , \alpha \end{aligned}$$

and

67 $$\begin{aligned} \beta ^2 = 0.5\beta ^1, \quad \beta ^3 = 0.5 \beta ^1\quad \text {and} \quad \beta ^4 = 0.25 \beta ^1. \end{aligned}$$

It will also be assumed that the initial infected population is small, so that, for each *i*

68 $$\begin{aligned} S_i(0;\epsilon ) = (1-10^{-4})N_i \quad \text {and} \quad I_i(0;\epsilon ) = 10^{-4}N_i. \end{aligned}$$

In the following examples, $\beta$ will be chosen so that the disease-free next-generation matrix of a completely unvaccinated population, given by

69 $$\begin{aligned} R_{ij} = \frac{N_i\beta ^1_{ij}}{\mu ^1_j} = \beta ^1_{ij} \end{aligned}$$

has a spectral radius (that is, largest eigenvalue) equal to 4. This sets the $R_0$ number in the overall population to be 4. To illustrate the population structure, Fig.  shows a heatmap of the matrix $R_{ij}$. This highlights the strongly assortative nature of the contacts (that is, members of a subgroup are most likely to be contacts with members of their own subgroup), while also showing that contacts are lower for older age groups.

**Fig. 5:**
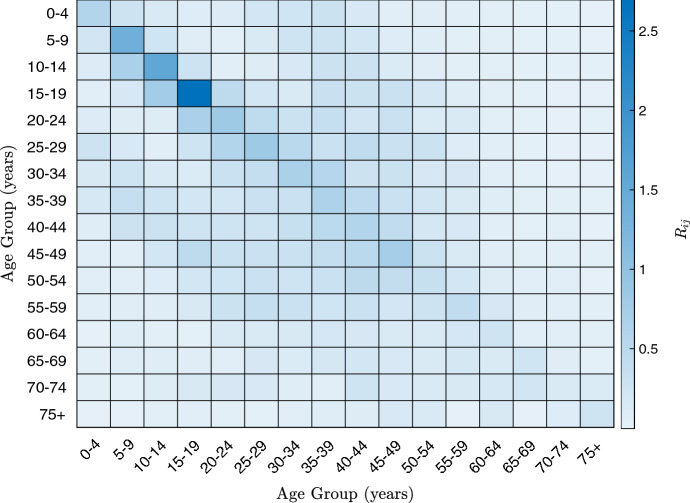
(Color Figure Online) A heatmap of the next-generation matrix for the age-structured UK population

Now, two different age-dependent case-fatality ratios will be considered—uniform case-fatality and approximate COVID-19 case fatality, taken from Dyer (2021). In both cases, it will be assumed that vaccination reduces the case fatality ratio by 90% (following the results of Dyer (2021) for the COVID-19 vaccines) so that $\kappa _i = 0.1$ for all *i*. However, it is worth emphasising that this model is simply based on real-world data and does not seek to accurately model the COVID-19 pandemic.

Figure  shows the effectiveness of vaccinating each age group in the two different cases, as a proportion of the optimal effectiveness. Note that here the proportion of effectiveness of assigning vaccine to group *i* is given by $\frac{y_i}{\min _j(y_j)}$, as each $y_j$ is non-positive. It highlights that the significantly higher mortality rates for COVID-19 for the older age groups mean that vaccinating them is much more effective than vaccinating the other age groups. This is an example of Theorems 1 and 2, as the oldest age group makes up a relatively small percentage (around 9%) of the population, but, if one scales *p* such that it has median value 1, the $p_iN_i$ value for the oldest age group is approximately 20, and so is definitely *O*(1) rather than $O(\epsilon )$.

**Fig. 6:**
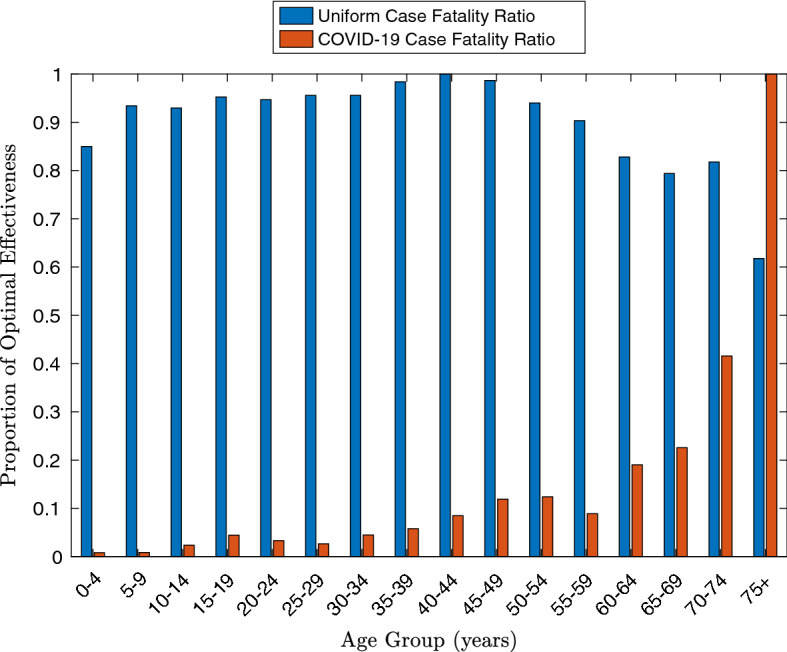
(Color Figure Online) The effectiveness of assigning a small quantity of vaccines to each age group as a proportion of the optimal effectiveness

A perhaps surprising exception to the general correlation between effectiveness and mortality is the relatively low effectiveness of vaccinating the 55–59-year-old age group, which is lower than the 45–49-year-old and 50–54-year-old groups. This illustrates the non-intuitive nature that optimal vaccination policies can take, and the importance of investigating their behaviour fully. The main reason for this low effectiveness is that, while the 55–59-year-old age group is more vulnerable to COVID-19 than the younger groups, according to Prem et al. (2017), they have much less contact with the 75+-year-old age group, and thus, vaccinating this group provides significantly less secondary protection to most vulnerable members of the population. The authors speculate that this could be due to a significant number of the parents of the 55–59-year-old age group having died (particularly in comparison with the younger groups), reducing their links with the 75+-year-old age group. Moreover, those in the 55–59-year-old age group may also not be old enough to have many 75+-year olds in their social circles (in comparison with members of older groups). However, further investigation would be needed to justify this claim.

In the case of uniform mortality, the vaccination policy becomes even less intuitive, as Fig. 6 shows that the optimal age group to vaccinate is the 40–44-year olds. Indeed, from Fig. 5, it may seem that the 15–19-year-old group would be the best group to vaccinate, as they have the highest overall transmission—that is, the maximum value of

70 $$\begin{aligned} \hbox { Total infectious force of group}\ j := \sum _{i=1}^{16}R_{ij}. \end{aligned}$$

However, if instead, one considers

71 $$\begin{aligned} \hbox { Total external infectious force of group}\ j := \sum _{i=1,i \ne j}^{16}R_{ij}, \end{aligned}$$

then it is the 35–39 and the 40–44 age groups which have the highest values. This can be considered in conjunction with the results of the previous subsection, which showed that vaccinating groups with $R_0$ numbers close to 1 is optimal for disconnected populations. Indeed, the “secondary effect” of vaccinations (that is, the number of people who are not vaccinated, but are protected from the disease because of vaccines given to others) can be higher for groups with lower internal infectious force, particularly when their external infectious force is higher.

Finally, it is useful to again explore the range of values for $\epsilon$ for which ${{\textbf {y}}}$ gives a good approximation of the true number of infections. As the minimum (scaled so that the total population size is 1) value of $N_i$ is 0.0498 in this case, $\epsilon$ will be tested at 0.0498. The results of this are shown in Fig. , which again illustrates the effectiveness of this approximation. Indeed, the largest error across either case is of order $10^{-4}$, which in turn is of order $\epsilon ^2{\varvec{y}}$. This suggests that the $o(\epsilon )$ correction term in Theorem 3 is significantly smaller than $\epsilon$, which increases the usefulness of this approximation. However, further investigation is needed to determine whether this correction is of $O(\epsilon ^2 {\varvec{y}})$ for all parameter values.

**Fig. 7:**
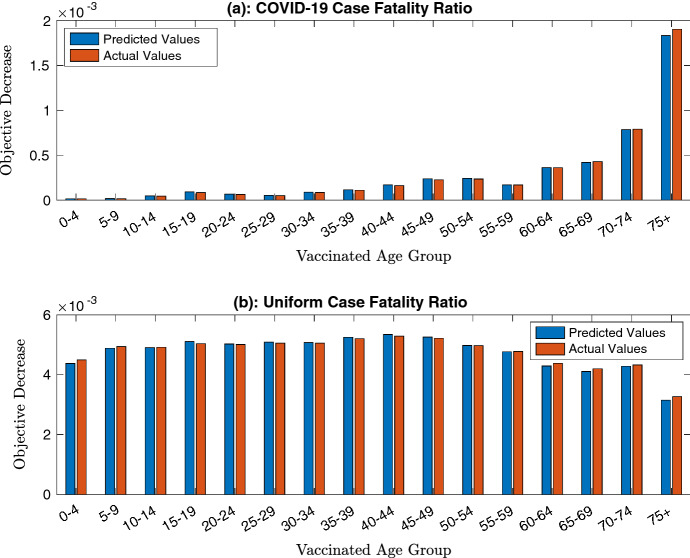
(Color Figure Online) A comparison of the predicted and simulated change in the objective function when vaccinating each individual group at $\epsilon = 0.0498$. Both the cases of a COVID-19 case fatality ratio (in **a**) and a uniform case fatality ratio (in **b**) are presented

## Discussion

This paper has shown two general principles for optimal vaccination policies by looking at the asymptotic behaviour of the optimal policy in the case of extreme parameters. Firstly, it has shown that small, vulnerable groups should in general be vaccinated first, regardless of the overall timetable of vaccination. This is an important result as it requires very little data on the population—merely the case fatality ratios and populations of the different subgroups—and in particular needs no forecasting of future transmission trends or vaccine supply.

The analytically derived results (in the limiting case) also show that the effect of vaccinating this small group far outweighs the effect of vaccinating any of the other groups. Indeed, if the size of the vulnerable group is $O(\epsilon )$ and the case fatality ratio of the other groups is $O(\epsilon )$, then Theorem 1 shows that vaccinating the vulnerable group will lead to an $O(\epsilon )$ decrease in the number of fatalities, while vaccinating the same number of people from another group will only decrease this by $O(\epsilon ^2)$. As discussed in Sect. 3.2.3, this result is of practical importance for diseases such as COVID-19, where the majority of the fatalities would be from certain age groups within the population. In particular, it provides strong evidence for the importance of sharing vaccines on a global scale, as this is the only way to ensure that vaccinations can be given to all people who are most vulnerable to the disease.

However, this result should be used with caution, as it certainly does not imply that a population should always be vaccinated in order of decreasing vulnerability to the disease. The optimal vaccination policy is, in general, a balance between directly protecting the vulnerable by vaccinating them and by indirectly protecting them by vaccinating those groups with the highest infectiousness. This is shown in Fig. 6 by the fact that, when a COVID-19 case fatality ratio is used, the relative effectiveness of vaccinating each age group does not decrease everywhere with age. The results of Theorems 1 and 2 simply provide a principle that in the asymptotic limit, the optimal strategy is to vaccinate small, vulnerable groups first. In the absence of data on vaccination effectiveness (which is crucial in determining whether indirectly protecting the more vulnerable population may be better), this provides a mathematically sound justification for beginning with the most vulnerable members of a population while gathering data to determine the rest of the vaccination policy.

The second principle derived in this paper was a linear approximation to the change in number of fatalities from a disease, which allows for the estimation of the optimal vaccination policy in the case of a small total supply. Again, this principle is flexible, applying for any set of parameters and provides a computationally cheap way of the approximating the optimal solution, even for large numbers of groups, as it merely requires the solution of a linear system involving the same number of variables as the number of groups.

A useful feature of this approximation is that it appears to have high accuracy even for reasonably large values of the total supply, such as when 10% of the population can be vaccinated. Figures 4 and 7 show that there is very little deviation between the predicted and actual values of the objective function and so suggest that this is a flexible and widely applicable method of approximation, even when the population contains a large number of subgroups. However, it would be helpful to strengthen the results of Theorem 3 to get a stronger bound on the error for small $\epsilon$ to ensure that this similarity holds for all models.

The results of the examples presented in Sect. 4 are also informative for vaccination policy. As shown in Fig.  4, in a completely homogeneous population, vaccination has the most effect when the reproduction number ($\frac{\beta }{\mu }$ in this case) is slightly bigger than 1, with a steep decline in effectiveness for reproduction numbers below 1 and a more gradual decline for large reproduction numbers. This result allows one to consider the “vaccination leverage” of a population—that is, the effectiveness that a small quantity of vaccination can have—and shows that, even in the case of homogeneous case fatality ratios, vaccinating in order of infectiousness may be far from optimal, as it is much more difficult to reduce infections in a highly infectious population.

Indeed, a similar idea was shown to apply when the UK age structure was considered. In the case of uniform case fatality, the optimal group to assign a small amount of vaccinations was the 40–44 age group which, as shown in Fig. 5, is not the most infectious group. This perhaps counter-intuitive result highlights the importance of mathematically justifying the principles one uses to decide on optimal vaccination policies, as “common-sense” arguments may in fact give false conclusions. Communicating such principles to governments and policy-makers will be crucial in future pandemics, particularly ones with more homogeneous case fatality ratios where the optimal policy is not as intuitive as for diseases like COVID-19.

An important limitation of Theorem 3 is that the optimal policies for small vaccination supplies do not necessarily generalise to give the beginning of the optimal vaccination policy in the case of a much larger vaccination supply. Indeed, it is possible to have bifurcations in the optimal vaccination policy as the supply increases—for example, it can become possible to completely avoid an epidemic by vaccinating a large quantity of an infectious group. Thus, while the linear approximation can be a useful starting point when attempting to estimate the optimal strategy, it is important to consider alternatives when a large proportion of the population can be vaccinated.

The results of this paper are only applicable if the trajectory of the disease in question can be well-approximated by multi-group SIR dynamics. In particular, this requires there to be reasonably high levels of the disease in a population [otherwise stochastic dynamics change the epidemic behaviour (Ball and Neal 2002)], and for population subgroups to be sufficiently large (again to prevent stochasticity dominating). Moreover, the model assumptions would not hold if individuals could be re-infected, or if the effect of vaccination was not eternal (though if the timescale of the epidemic was sufficiently shorter than the timescale of immunity decay, then the model would still provide a good approximation).

A final barrier to using the results in this paper is that estimation error in the model parameters could lead to the optimal solutions being incorrectly calculated. Estimating the $\beta _{ij}^{\alpha }$ parameters is particularly complicated, especially in a multi-group setting where it is difficult to establish the chain of transmission between different groups. Because of this, building models based on contact rates between groups [estimated using surveys (Prem et al. 2017)] or proxies such as commuting patterns (Keeling and White 2011) may be the best method, at least to provide priors on the parameters. Theorems 1 and 2 are significantly less susceptible to errors in parameters, as they do not require any of the $\beta _{ij}^{\alpha }$ or $\mu _j^{\alpha }$ parameters to be known, although the level of “smallness” of $\epsilon$ would vary depending on the disease in question. Theorem 3 is significantly more susceptible to error, as all the model parameters are needed. However, while there may be bifurcations in the optimal strategy, the optimal value of the objective function should depend continuously on the parameters (a fact which could be proved by extending the results of Proposition 5), limiting the effect of small estimation errors.

Despite this, the authors expect that similar results to those presented in Theorem 3 will hold for a very wide class of deterministic models. Essentially, the only necessary characteristic of the model that is required by Theorem 3 is that the objective function, $H({\varvec{U}})$, is a continuously differentiable function of the vaccination policy ${\varvec{U}}$ in some neighbourhood of ${\varvec{0}}$. Indeed, ${\varvec{y}}$ in Theorem 3 can be replaced by $\nabla {\varvec{H}}({\varvec{0}})$ in a general setting. Certainly, it should be conceptually simple (though perhaps algebraically complicated) to generalise this result to other compartmental models such as SEIR (Susceptible–Exposed–Infected–Recovered) and even those modelling vector-transmitted diseases.

The authors also expect that Theorem 1 will hold for general models where the effect of vaccination is eternal. The essential points in the proof of Theorem 1 are that vaccinating the small group does not affect the overall vaccination program (to leading order) and that it does have an O(1) effect on the objective function. Both of these should still hold in a wide range of models, although it may be difficult to define the meaning of “very small group” and “very vulnerable group”—particularly in more complicated settings such as individual-based models.

This work could be extended by deriving more principles for extreme parameter values and investigating whether they generalise to realistic model parameters. By combining the existing results in this paper and others such as Gavish and Katriel (2022) with potential new ones, one could create an algorithm that creates good heuristics of optimal vaccination policies that could be used as starting points for accurately approximating the optimal policy for a general parameter set. This could have significant implications for the design of vaccination policies, as it would enable the optimisation problem to be estimated for very complex models, as the time taken to converge to an optimal solution would significantly decrease given good initial heuristics.

## Conclusion

The results of this paper are summarised below:

- If a sufficiently vulnerable, sufficiently small population exists in a multi-group SIR model, it is optimal to vaccinate this group first.
- For small overall vaccination supplies, the optimal vaccination problem can be well approximated by a simple knapsack problem.
- This linearisation appears to be a good approximation even for relatively large vaccination supplies (such as 10% of the population).
- This linearisation shows that, in the case of uniform case fatality, it is not necessarily optimal to vaccinate the most infectious group.

## Data Availability

The data and code used in this study are available from https://github.com/mpenn114/AsymptoticVaccination.

## Appendix A: Proof of Theorem 1

Note that, throughout this section, the tilde will be removed from the rescaled $p_i$ terms to reduce notation (and hence all $p_i$ terms used here will be assumed to be rescaled).

### Theorem 1

Suppose that for all $\epsilon > 0$

A1 $$\begin{aligned} N_1(\epsilon ) = \epsilon , \quad S_1(0;\epsilon ) = \epsilon \sigma , \quad I_1(0;\epsilon ) = (1-\sigma )\epsilon \quad \text {and} \quad p_1(\epsilon ) = \frac{1}{\epsilon } \end{aligned}$$

for some $\sigma \in (0,1)$. Suppose that all other parameter values and initial conditions are independent of $\epsilon$.

Consider any vaccination policy given by ${\varvec{U}}(t;\epsilon )$ and suppose that there exists fixed $\alpha ,\tau , w > 0$ such that

A2 $$\begin{aligned} W_1(\tau ;\epsilon ) < \alpha \epsilon \quad \text {and} \quad \sum _{i=1}^n W_i(\tau ;\epsilon )> w \quad \forall \epsilon >0. \end{aligned}$$

Define a new policy $\tilde{{\varvec{U}}}(t;\epsilon )$

A3 $$\begin{aligned} {\tilde{U}}_1(t;\epsilon ) = \left\{ \begin{matrix} \sum \nolimits _{i=1}^n U_i(t) &{} \text {if } \sum \nolimits _{i=1}^n W_i(t;\epsilon ) \le \epsilon \\ 0 &{} \text {otherwise} \\ \end{matrix} \right. \end{aligned}$$

and, for $$i \ne 1$$

A4 $$\begin{aligned} {\tilde{U}}_i(t;\epsilon ) = \left\{ \begin{matrix}0 &{} \text {if } \sum \nolimits _{i=1}^n W_i(t;\epsilon ) \le \epsilon \\ U_i(t;\epsilon ) &{} \text {otherwise} \\ \end{matrix} \right. . \end{aligned}$$

Suppose that for each $i \in \{1,\ldots ,n\}$ and $t \ge 0$,

A5 $$\begin{aligned} |W_i(t;0) -W_i(t;\epsilon )|\le \epsilon . \end{aligned}$$

Define

A6 $$\begin{aligned} \Pi (\epsilon ) := \{ i : \exists t \ge 0 \quad \text {s.t.} \quad I_i(t;\epsilon ) > 0\} \end{aligned}$$

and suppose that $\Pi (\epsilon ) = \{1,\ldots ,n\}$ for any $\epsilon > 0$ and that $\Pi (0) = \{2,\ldots ,n\}$. Finally, suppose that there exists a $i \in \{2,\ldots ,n\}$ such that

A7 $$\begin{aligned} \beta _{1i}^1>\beta _{1i}^3 \ge 0. \end{aligned}$$

Then, the policy $\tilde{{\varvec{U}}}$ is feasible and for sufficiently small $\epsilon$,

A8 $$\begin{aligned} H({\varvec{U}}(t;\epsilon )) > H(\tilde{{\varvec{U}}}(t;\epsilon )). \end{aligned}$$

### Proof

It is first important to prove that the $\tilde{{\varvec{U}}}$ is feasible. Firstly,

A9 $$\begin{aligned} \sum _{i=1}^n {\tilde{U}}_i(t;\epsilon ) \le \sum _{i=1}^nU_i(t;\epsilon ) \end{aligned}$$

which, as ${\varvec{U}}$ is feasible, means that the supply and rate constraints are satisfied. Moreover, as each $U_i(t;\epsilon ) \ge 0$,

A10 $$\begin{aligned} {\tilde{U}}_i(t;\epsilon ) \ge 0 \quad \forall i \in \{1,\ldots ,n\}. \end{aligned}$$

Also, for $$i \ne 1$$,

A11 $$\begin{aligned} {\tilde{U}}_i(t;\epsilon ) \le U_i(t;\epsilon ) \Rightarrow {\tilde{W}}_i(t;\epsilon ) \le W_i(t;\epsilon ) \le N_i. \end{aligned}$$

Finally, define

A12 $$\begin{aligned} t^* := \sup \left\{ t : \sum _{i=1}^nW_i(t;\epsilon ) \le \epsilon \right\} \in \Re \cup \{\infty \} \end{aligned}$$

and then

A13 $$\begin{aligned} U_1(t;\epsilon ) \le \int _0^{t^*} \sum _{i=1}^nU_i(s;\epsilon ){\textrm{d}}s \le \epsilon = N_1 \end{aligned}$$

as required. $$\square $$

Define $S_i(t;\epsilon )$ to be the number of susceptibles given the parameters $N_1(\epsilon )$, $S_1(0;\epsilon )$ and $I_1(0;\epsilon )$ and the vaccination policy ${\varvec{U}}(t;\epsilon )$, and define ${\tilde{S}}_i(t;\epsilon )$ to be the number of susceptibles given the parameters $N_1(\epsilon )$, $S_1(0;\epsilon )$ and $I_1(0;\epsilon )$ and the vaccination policy $\tilde{{\varvec{U}}}(t;\epsilon )$. Use similar definitions for the other variables in the model.

### Proposition 4

#### Proposition 4

For each $t \ge 0$ and $i \in \{1,\ldots ,n\}$,

A14 $$\begin{aligned} |{\tilde{W}}_i(t;\epsilon ) - {\tilde{W}}_i(0;\epsilon )|\le 2\epsilon . \end{aligned}$$

#### Proof

Firstly, note that

A15 $$\begin{aligned} {\tilde{W}}_1(t;\epsilon ) \le \epsilon \end{aligned}$$

so

A16 $$\begin{aligned} |{\tilde{W}}_1(t;\epsilon ) - {\tilde{W}}_1(0;\epsilon )|\le \epsilon . \end{aligned}$$

Now, suppose that $i \ne 1$. Then, for each $\epsilon , t \ge 0$, with $t^*$ defined as in (A12),

A17 $$\begin{aligned} |W_i(t;\epsilon ) - {\tilde{W}}_i(t;\epsilon )|&= \bigg |\int _{0}^{t}U_i(s){\textrm{d}}s - \int _{t^*}^{\max (t,t^*)}U_i(s){\textrm{d}}s\bigg |. \end{aligned}$$

If $$t < t^*$$, then

A18 $$\begin{aligned} |W_i(t;\epsilon ) - {\tilde{W}}_i(t;\epsilon )|\le \bigg |\int _0^{t}U_i(s){\textrm{d}}s\bigg |\le \bigg |\int _0^{t^*}U_i(s){\textrm{d}}s\bigg |\le \epsilon \end{aligned}$$

while if $t \ge t^*$, then

A19 $$\begin{aligned} |W_i(t;\epsilon ) - {\tilde{W}}_i(t;\epsilon )|=\bigg |\int _0^{t^*}U_i(s){\textrm{d}}s\bigg |\le \epsilon . \end{aligned}$$

Thus, noting

A20 $$\begin{aligned} W_i(t;0) = {\tilde{W}}_i(t;0) \end{aligned}$$

and using (34),

A21 $$\begin{aligned} |{\tilde{W}}_i(t;\epsilon ) - {\tilde{W}}_i(0;\epsilon )|\le |{\tilde{W}}_i(t;\epsilon )-W_i(t;\epsilon )|+ |W_i(t;\epsilon ) - W_i(t;0)|\le \epsilon + \epsilon = 2 \epsilon \nonumber \\ \end{aligned}$$

as required. $$\square $$

### Proposition 5

Next, it is helpful to consider the continuous dependence of the final size of the epidemic on the initial conditions and the vaccination policy. A weaker result is proved in Penn and Donnelly (2022) (and is referenced in this proof as Lemma 14). However, that result only holds for finite times, and extending it to hold for the final sizes requires a significant amount of extra work.

#### Proposition 5

Suppose that the $U_i$ have uniformly bounded support for each $\epsilon > 0$. Moreover, for each of the model variables, $f_i$, suppose that

A22 $$\begin{aligned} |f_i(0;\epsilon ) - f_i(0;0)|< K\epsilon \end{aligned}$$

for some constant *K* and that

A23 $$\begin{aligned} |W_i(t;\epsilon ) - W_i(t;0)|< K'\epsilon \end{aligned}$$

for some constant $K'$. Finally, suppose all parameters are independent of $\epsilon$ with the exception that $N_1(\epsilon ) = \epsilon$. Then, for each $\delta > 0$, there exists some $\Delta > 0$ such that

A24 $$\begin{aligned} \epsilon \in [0,\Delta ] \Rightarrow |f_i(\infty ;\epsilon ) - f_i(\infty ;0)|< \delta \quad \forall f \in \{I_i(t;\epsilon ),I_i^V(t;\epsilon ),R_i(t;\epsilon ),R^V_i(t;\epsilon )\}.\nonumber \\ \end{aligned}$$

Note that this holds both in the case of Theorem 1 (where $N_1 \rightarrow 0$, $\Pi (\epsilon ) = \{1,\ldots ,n\}$ for $\epsilon > 0$ and $\Pi (0) = \{2,\ldots ,n\}$) or, in the case where each $N_i$ is independent of $\epsilon$ (by adding a disconnected group of size $\epsilon$).

#### Proof

Choose any $\delta > 0$. Now, it is possible to write the system for ${\varvec{I}}$ and ${\varvec{I}}^V$ in the form

A25 $$\begin{aligned} \frac{{\textrm{d}}{\varvec{J}}(t;\epsilon )}{{\textrm{d}}t} = {\varvec{M}}(t;\epsilon ){\varvec{J}}(t;\epsilon ), \end{aligned}$$

where ${\varvec{M}}$ depends on the values of ${\varvec{S}}(t;\epsilon )$, ${\varvec{S}}^V(t;\epsilon )$, $\beta ^{\alpha }_{ij}$ and $\mu ^{\alpha }_i$ and

A26 $$\begin{aligned} {\varvec{J}} = \begin{pmatrix} {\varvec{I}}\\ {\varvec{I}}^V \end{pmatrix}. \end{aligned}$$

Hence, in particular, by using Proposition 4 and Lemma 14 for any fixed $t \ge 0$,

A27 $$\begin{aligned} \lim _{\epsilon \rightarrow 0}(M(t;\epsilon )) = M(t;0). \end{aligned}$$

Moreover, if the support of each $U_i(t;\epsilon )$ is bounded by $t_U$ (which exists by assumption), then for $t > t_U$, each $S_i(t;\epsilon )$ and $S^V_i(t;\epsilon )$ is non-increasing in *t* and so ${\varvec{M}}(t;\epsilon )$ is non-increasing. As it is bounded below, it therefore must converge to some matrix ${\varvec{M}}(\infty ;\epsilon )$, and, for $t > t_U$,

A28 $$\begin{aligned} \frac{{\textrm{d}}{\varvec{J}}(t;\epsilon )}{{\textrm{d}}t} \ge {\varvec{M}}(\infty ;\epsilon ) {\varvec{J}}(t;\epsilon ). \end{aligned}$$

Hence, by Lemma 9,

A29 $$\begin{aligned} {\varvec{J}}(t_U+t';\epsilon ) \ge e^{t'{\varvec{M}}(\infty ;\epsilon )} {\varvec{J}}(t_U;\epsilon ). \end{aligned}$$

Moreover, by Lemma 11,

A30 $$\begin{aligned} \lim _{t' \rightarrow \infty }\left( {\varvec{J}}(t_U+t';\epsilon )\right) = 0 \end{aligned}$$

and hence (by non-negativity)

A31 $$\begin{aligned} \lim _{t' \rightarrow \infty } \left( e^{t'{\varvec{Q}}(\infty ;\epsilon )}{\varvec{J}}(t_U;\epsilon )\right) = 0. \end{aligned}$$

Now, define

A32 $$\begin{aligned} \max _{i,\alpha }(\mu _i^{\alpha }) := \mu _{\max } \end{aligned}$$

and then define

A33 $$\begin{aligned} \varvec{{\mathcal {M}}}(\infty ;0) := {\varvec{M}}(\infty ;0) + \mu _{\max } \varvec{{\mathcal {I}}}_{2n}, \end{aligned}$$

where $\varvec{{\mathcal {I}}}_{2n}$ is the 2*n* by 2*n* identity matrix. Thus, in particular, $\varvec{{\mathcal {M}}}(\infty ;0)$ is non-negative and so

A34 $$\begin{aligned} e^{{\varvec{M}}(\infty ;0)} =e^{-\mu _{\max }}e^{\varvec{{\mathcal {M}}}(\infty ;0)} \end{aligned}$$

is non-negative as the exponential of a non-negative matrix is non-negative (as it is a weighted sum of powers of that matrix with positive weights). Thus, by Perron–Frobenius theory, summarised in Berman and Plemmons (1994), there exists a real non-negative eigenvalue $\lambda (\infty ;0)$ (called the Perron eigenvalue) of $e^{M(\infty ;0)}$ such that any other eigenvalues $\rho (\infty ;0)$ satisfy

A35 $$\begin{aligned} |\rho (\infty ;0)|\le |\lambda (\infty ;0)|\end{aligned}$$

so, in particular

A36 $$\begin{aligned} \Re (\rho (\infty ;0)) \le \Re (\lambda (\infty ;0)). \end{aligned}$$

$$\square $$

#### Claim

$$0<|\lambda (\infty ;0)|< 1$$

#### Proof

Note that $\lambda (\infty ;0) > 0$, as

A37 $$\begin{aligned} \text {trace}\bigg ( e^{{\varvec{M}}(\infty ;0)}\bigg ) \ge \text {trace}\bigg (e^{-\mu _{\max }}\varvec{{\mathcal {I}}}_{2n}\bigg ) > 0 \end{aligned}$$

and thus, $$\lambda (\infty ;0)\ne 0$$.

From Berman and Plemmons (1994), there is a non-negative eigenvector, ${\varvec{v}}$, with eigenvalue $\lambda (\infty ;0)$. Now, ${\varvec{v}}$ must be an eigenvector of ${\varvec{M}}(\infty ;0)$ (as eigenvectors of a matrix and its exponential are the same). Thus, there is some $\lambda ^*(\infty ;0)$ such that

A38 $$\begin{aligned} {\varvec{M}}(\infty ;0){\varvec{v}} = \lambda ^*(\infty ;0) {\varvec{v}}. \end{aligned}$$

In particular, writing ${\varvec{v}} = (v_1,\ldots ,v_{2n})^T$

A39 $$\begin{aligned} \lambda ^*(\infty ;0) v_1= ({\varvec{M}}(\infty ;0){\varvec{v}})_1 = -\mu _1^1 v_1 \end{aligned}$$

and thus, either $\lambda ^*(\infty ;0)= -\mu _i^1 < 0$ or $v_1 = 0$. Suppose first that $\lambda ^*(\infty ;0)= -\mu _1^1$. Then, this means that (as the eigenvalues of $e^{{\varvec{M}}(\infty ;0)}$ are the exponentials of the eigenvalues of ${\varvec{M}}(\infty ;0)$),

A40 $$\begin{aligned} |\lambda (\infty ;0)|= |e^{-\mu _1^1}|< 1. \end{aligned}$$

Similarly, $v_{n+1} \ne 0$ implies that

A41 $$\begin{aligned} |\lambda (\infty ;0)|= |e^{-\mu _1^2}|< 1. \end{aligned}$$

Thus, suppose for the remainder of the proof of this claim that $v_1 = v_{n+1} = 0$. Now, for $i \le n$, the entries on the ith row of ${\varvec{M}}(\infty ;0)$ are given by:

A42 $$\begin{aligned} M(\infty ;0)_{ij} = \left\{ \begin{matrix} S_i(\infty ;0) \beta ^1_{ij} - \delta _{ij}\mu _i^1 &{} \text {if} j \le n\\ \\ S_{i-n}(\infty ;0) \beta _{i(j-n)}^3 &{}\text {if} j > n \\ \end{matrix}\right. \end{aligned}$$

and for $i > n$, they are given by

A43 $$\begin{aligned} M(\infty ;0)_{ij} = \left\{ \begin{matrix} S^V_i(\infty ;0) \beta ^2_{ij} &{} \text {if} j \le n\\ \\ S_{i-n}^V(\infty ;0) \beta _{i(j-n)}^4 - \delta _{ij}\mu _i^2 &{}\text {if} j > n \\ \end{matrix}\right. , \end{aligned}$$

where $\delta _{ij}$ is the Kronecker delta.

Now, as $\Pi (0) = \{2,\ldots ,n\}$, by Lemma 13, it is necessary that

A44 $$\begin{aligned} J_i(t;0)> 0 \quad \forall t> 0 \quad \text {and} \quad i \in \{2,\ldots ,n\}. \end{aligned}$$

Moreover, if $I^V_i(t;0) = 0$ for some $t > 0$, then, by Lemma 15, as $\Pi (0) = \{2,\ldots ,n\}$, it is necessary that

A45 $$\begin{aligned} S^V_i(t;0)\beta _{ji}^3 = S^V_i(t;0)\beta _{ji}^4 = 0 \quad \forall j \in \{2,\ldots ,n\} \end{aligned}$$

and so, if $t \ge t_U$, then this implies

A46 $$\begin{aligned} S^V_i(\infty ;0)\beta _{ji}^3 = S^V_i(\infty ;0)\beta _{ji}^4 = 0 \quad \forall j \in \{2,\ldots ,n\}. \end{aligned}$$

Thus, in this case, for $j \notin \{1,n+1\}$

A47 $$\begin{aligned} M(\infty ;0)_{i j} = -\mu _{(i-n)}^2\delta _{ij}. \end{aligned}$$

Therefore, suppose ${\varvec{J}}_i(t_U;0) = 0$ for some $i \notin \{1,n+1\}$ (and so, necessarily, $i \in \{n+2,\ldots ,2n\}$). Then,

A48 $$\begin{aligned} ( {\varvec{M}}(\infty ;0){\varvec{v}})_i&= \sum _{j=1}^{2n}M(\infty ;0)_{ij}v_j \end{aligned}$$

A49 $$\begin{aligned}&= M\infty ;0)_{i1}v_1 + M(\infty ;0)_{i(n+1)}v_{(n+1)}+ M(\infty ;0)_{ii}v_i\end{aligned}$$

A50 $$\begin{aligned}&= -\mu _{i}^2 v_i \end{aligned}$$

and so

A51 $$\begin{aligned} |\lambda (\infty ;0)|= |e^{-\mu _i^2}|< 1. \end{aligned}$$

Consequently, this holds if any ${\varvec{J}}_i(t_U;0) = 0$. Conversely, suppose that ${\varvec{J}}_i(t_U;0)\ne 0$ for all $i \notin \{1,(n+1)\}$. Then, there exists some $\alpha > 0$ and some non-negative vector ${\varvec{w}}$ such that

A52 $$\begin{aligned} {\varvec{J}}(t_U;0) = \alpha {\varvec{v}} + {\varvec{w}}. \end{aligned}$$

Therefore, for any positive integer *n*,

A53 $$\begin{aligned} e^{n {\varvec{M}}(\infty ;0)}{\varvec{J}}(t_U;0) = e^{n {\varvec{M}}(\infty ;0)}(\alpha {\varvec{v}} + {\varvec{w}}) = \lambda (\infty ;0)^n\alpha {\varvec{v}} + e^{n {\varvec{M}}(\infty ;0) }{\varvec{w}} \ge \lambda (\infty ;0)^n\alpha {\varvec{v}}.\nonumber \\ \end{aligned}$$

Now, ${\varvec{v}}$ is an eigenvector so it has a nonzero component, which means that

A54 $$\begin{aligned} \bigg ( \lim _{n \rightarrow \infty }( e^{n M(\infty ;0)}{\varvec{J}}(t_U;0)) = {\varvec{0}}\bigg ) \Rightarrow \bigg (\lim _{n \rightarrow \infty }( \lambda (\infty ;0)^n\alpha {\varvec{v}}) = {\varvec{0}}\bigg ) \Rightarrow \bigg (|\lambda (\infty ;0)|< 1\bigg )\nonumber \\ \end{aligned}$$

and so $|\lambda (\infty ;0)|< 1$ holds in all cases, which finishes the proof of this claim.

#### Claim

There exists some constant *X* independent of $\delta$ such that $\int _T^{\infty }J_i(s;\epsilon ){\textrm{d}}s \le X\delta$

#### Proof

Now, the exponentials of the eigenvalues of ${\varvec{M}}(\infty ;0)$ are the eigenvalues of $e^{{\varvec{M}}(\infty ;0)}$ which means that, if $\eta (\infty ;0)$ is an eigenvalue of ${\varvec{M}}(\infty ;0)$ then there exists some $\kappa > 0$ such that

A55 $$\begin{aligned} |e^{\eta (\infty ;0)}|\le |\lambda (\infty ;0)|< e^{-4\kappa }< 1 \Rightarrow |e^{\Re (\eta (\infty ;0))}|< e^{-4\kappa } \Rightarrow \Re (\eta ) < -4\kappa \qquad \end{aligned}$$

and so all eigenvalues of ${\varvec{M}}(\infty ;0)$ have strictly negative real part. Thus, by continuous dependence of eigenvalues on the matrix, as ${\varvec{M}}(t;0)$ converges to ${\varvec{M}}(\infty ;0)$ as $t \rightarrow \infty$, there exists some $T > t_U$ such that

A56 $$\begin{aligned} \Re (\eta (t;0)) < -2\kappa \quad \forall t > T \end{aligned}$$

where $\eta (t;0)$ is an eigenvalue of *M*(*t*; 0). Now, fix $\delta > 0$. From Lemma 11, by choosing *T* to be sufficiently large, one can assume that

A57 $$\begin{aligned} J_i(T;0) <\delta \quad \forall i \in \{1,\ldots ,2n\}. \end{aligned}$$

Moreover, there exists some $\Delta$ (which is dependent on *T*) such that

A58 $$\begin{aligned} \Re (\eta (T;\epsilon )) < -\kappa \quad \forall \epsilon \in [0,\Delta ]. \end{aligned}$$

Now, similarly, by choosing $\Delta$ to be sufficiently small, one can assume that by Lemma 14

A59 $$\begin{aligned} |J_i(t;\epsilon ) - J_i(t;0)|< \delta \quad \forall t< T \Rightarrow |J_i(T;\epsilon )|< 2\delta \quad \forall i \in \{1,\ldots ,2n\}\quad \text {and} \quad \forall \epsilon \in [0,\Delta ]\nonumber \\ \end{aligned}$$

and , for all $t < T$,

A60 $$\begin{aligned} |R_i(T;\epsilon ) - R_i(T;0)|, |R^V_i(T;\epsilon ) - R^V_i(T;0)|< \delta \quad \forall i \in \{1,\ldots ,2n\}, \quad \text {and} \quad \forall \epsilon \in [0,\Delta ].\nonumber \\ \end{aligned}$$

Now, for any $t > 0$,

A61 $$\begin{aligned} {\varvec{M}}(t+T;\epsilon ) \le {\varvec{M}}(T;\epsilon ). \end{aligned}$$

Thus, as the solution to the system

A62 $$\begin{aligned} \frac{{\textrm{d}}{\varvec{z}}}{{\textrm{d}}t} = {\varvec{M}}(T;\epsilon ){\varvec{z}}, \quad {\varvec{z}}(0) = {\varvec{J}}(T;\epsilon ) \end{aligned}$$

is

A63 $$\begin{aligned} {\varvec{z}}(t) = e^{{\varvec{M}}(T;\epsilon )t}{\varvec{J}}(T;0); \end{aligned}$$

one has, by Lemma 9,

A64 $$\begin{aligned} {\varvec{J}}(t+T;\epsilon ) \le e^{{\varvec{M}}(T;\epsilon )t}{\varvec{J}}(T;0). \end{aligned}$$

Now, noting that ${\varvec{M}}(T;\epsilon )$ is invertible as all its eigenvalues have strictly negative real part, for any $t > 0$

A65 $$\begin{aligned} \int _T^{t+T}{\varvec{J}}(s;\epsilon )ds&\le \int _0^{t}e^{{\varvec{M}}(T;\epsilon )s}{\varvec{J}}(T;\epsilon )ds \end{aligned}$$

A66 $$\begin{aligned}&= {\varvec{M}}(T;\epsilon )^{-1} (e^{M(T;\epsilon )t}{\varvec{J}}(T;\epsilon ) - {\varvec{J}}(T;\epsilon )) \end{aligned}$$

and so, taking *t* to $\infty$ and noting that all eigenvalues of $e^{M(T;\epsilon )}$ have real part less than 1 shows that

A67 $$\begin{aligned} \int _T^{\infty }{\varvec{J}}(s;\epsilon ){\textrm{d}}s \le -{\varvec{M}}(T;\epsilon )^{-1}{\varvec{J}}(T;\epsilon ). \end{aligned}$$

Now, each element of ${\varvec{M}}(t;\epsilon )$ is uniformly bounded (for any bounded range of $\epsilon$ and all $t \ge 0$) as the parameters and variables are uniformly bounded. Thus, by expressing the inverse in terms of determinants of sub-matrices of ${\varvec{M}}(t;\epsilon )$ (each of which must be uniformly bounded as ${\varvec{M}}(t;\epsilon )$ is uniformly bounded) by Cramer’s rule (Blyth and Robertson 2002), one can see that there exists a constant $M^*$ such that for each *i* and *j*,

A68 $$\begin{aligned} \text {det}({\varvec{M}}(t;\epsilon )) \ne 0 \Rightarrow |{\varvec{M}}(t;\epsilon )^{-1}_{ij}|\le \bigg |\frac{M^*}{\text {det}(M(t;\epsilon ))}\bigg |. \end{aligned}$$

Note that

A69 $$\begin{aligned} |\text {det}({\varvec{M}}(T;\epsilon ))|= \left|\prod _{\lambda \text { eigenvalue of}\ {\varvec{M}}(T;\epsilon )}(\lambda )\right|\ge \kappa ^n \end{aligned}$$

because all eigenvalues of $M(T;\epsilon )$ have real part at most $-\kappa$ and hence modulus at least $\kappa$. Thus, there exists some constant *X* (independent of $\delta$) such that for each *i* and *j*,

A70 $$\begin{aligned} \bigg |{\varvec{M}}(T;\epsilon )^{-1}_{ij}\bigg |\le \frac{X}{4n}. \end{aligned}$$

Thus, by the conditions on ${\varvec{J}}(T;\epsilon )$,

A71 $$\begin{aligned} \int _T^{\infty }J_i(s;\epsilon )ds \le X\delta \end{aligned}$$

which completes the proof of this claim. $\square$

As all the parameters and variables are uniformly bounded for all $\epsilon$, there exists a constant *Y* (independent of $\delta$) such that

A72 $$\begin{aligned} \bigg |\frac{{\textrm{d}}J_i}{{\textrm{d}}t}\bigg |\le Y \quad \forall i \in \{1,\ldots ,2n\}. \end{aligned}$$

Now, suppose there exists some $J_i(t;\epsilon ) > \delta ^{\frac{1}{3}}$ for $t > T$ and $\epsilon \in [0,\eta _1]$. Then, by non-negativity of $J_i$

A73 $$\begin{aligned} \int _T^{\infty }J_i(s;\epsilon ){\textrm{d}}s \ge \int _{t }^{t+\delta ^{\frac{1}{2}}} J_i(s;\epsilon ) {\textrm{d}}s \ge \int _0^{\delta ^{\frac{1}{2}}} \delta ^{\frac{1}{3}} - Ys {\textrm{d}}s = \delta ^{\frac{5}{6}} -\frac{Y}{2}\delta . \end{aligned}$$

Thus, taking $\delta$ sufficiently small such that

A74 $$\begin{aligned} \delta ^{\frac{5}{6}} -\frac{Y}{2}\delta > X\delta \end{aligned}$$

gives a contradiction. This means that, for each $i \in \{1,\ldots ,2n\}$

A75 $$\begin{aligned} J_i(t;\epsilon ) \le \delta ^{\frac{1}{3}} \quad \forall t \ge T \quad \text {and} \quad \forall \epsilon \in [0,\Delta ] \end{aligned}$$

and hence, combining this with (A59) (and assuming $\delta < 1$ so $\delta < \delta ^{\frac{1}{3}}$) shows that

A76 $$\begin{aligned} |J_i(t;\epsilon ) - J_i(t;0)|\le \delta ^{\frac{1}{3}} \quad \forall t \quad \text {and} \quad \forall i \in \{1,\ldots ,2n\}. \end{aligned}$$

Moreover, by (A71), for any $t>0$

A77 $$\begin{aligned} |R_i(T+t;\epsilon ) - R_i(T+t;0)|&\le |R_i(T;\epsilon ) - R_i(T;0)|+ |R_i(T;0) - R_i(T+t;0)|\end{aligned}$$

A78 $$\begin{aligned}&\le \delta + |R_i(T+t;\epsilon ) - R_i(T;\epsilon )|+ |R_i(T+t;0)\nonumber \\ {}&\quad - R_i(T;0)|\end{aligned}$$

A79 $$\begin{aligned}&\le \delta +2X\mu _i^1(\epsilon )\delta + 2X\mu _i^1(0)\delta \end{aligned}$$

A80 $$\begin{aligned}&\le X^* \delta \end{aligned}$$

for some constant $X^*$, alongside an identical result for $R^V_i$. Combining this with (A60) (and redefining $\delta \rightarrow \delta ^3$), the result of the proposition is proved.

### Theorem 1

Note that Proposition 5 also holds for the vaccination policies $\tilde{{\varvec{U}}}(t;\epsilon )$, using Proposition 4. Thus, one can define a function $\delta (\epsilon )$ such that for all sufficiently small $\epsilon$

A81 $$\begin{aligned} |f_i(t;\epsilon ) - f_i(t;0)|,|{\tilde{f}}_i(t;\epsilon ) - f_i(t;0)|\le \delta (\epsilon ) \quad \forall f \in \{I,I^V,R,R^V\} \end{aligned}$$

and

A82 $$\begin{aligned} \delta (\epsilon ) = o(1) \quad \text {as } \epsilon \rightarrow 0. \end{aligned}$$

Then, using, for example

A83 $$\begin{aligned} |R_i(\infty ;\epsilon ) - {\tilde{R}}_i(\infty ;\epsilon )|\le |R_i(\infty ;\epsilon ) - {\tilde{R}}_i(\infty ;0)|+ |R_i(\infty ;\epsilon ) - {\tilde{R}}_i(\infty ;0)|\qquad \end{aligned}$$

(as $R(\infty ;0) = {\tilde{R}}(\infty ;0)$) shows that

A84 $$\begin{aligned} |R_i(\infty ;\epsilon ) - {\tilde{R}}_i(\infty ;\epsilon )|, |R^V_i(\infty ;\epsilon ) - {\tilde{R}}^V_i(\infty ;\epsilon )|< 2\delta (\epsilon ) \quad \forall \epsilon \in [0,\eta ] \qquad \end{aligned}$$

which means

A85 $$\begin{aligned} \bigg |\sum _{j=2}^n p_j\left( R_j(\infty ;\epsilon ) + \kappa _jR^V_j(\infty ;\epsilon )\right) - \sum _{j=2}^np_j\left( {\tilde{R}}_j(\infty ;\epsilon ) +\kappa _j{\tilde{R}}^V_j(\infty ;\epsilon )\right) \bigg |= O(\delta ).\nonumber \\ \end{aligned}$$

Thus, the aim of the remainder of the proof is to show that the leading-order changes to $R_1(\infty ;\epsilon )$ are of exactly $O(\epsilon )$, and so $p_1R_1(\infty ;\epsilon )$ changes by an *O*(1) amount, meaning these changes to the objective function will eventually dominate the other changes given in (A85). This can be done by taking advantage of the fact that the quantities $f_1(t;\epsilon )$ are small, and so a linearised version of the equations for group 1 can be used.

Before beginning this process, it is helpful to note the following. From (A56) in the proof of Proposition 5, there exists some $T^* > t_U$ independent of $\delta$ and $\epsilon$ such that

A86 $$\begin{aligned} \lambda (T^*;0)< e^{-2 \kappa } < 1 \end{aligned}$$

where $\lambda (T^*;0)$ is the (necessarily real and non-negative) Perron eigenvalue of $e^{M(T^*;0)}$ (and is the exponential of the $\eta (\infty ;0)$ referenced in (A56)). Moreover, by the continuity of eigenvalues on the entries of the matrix, there exists some $\Delta > 0$ such that the analogously defined $\lambda (T^*;\epsilon )$ also satisfies

A87 $$\begin{aligned} \lambda (T^*;\epsilon )< e^{-\kappa } < 1 \quad \forall \epsilon \in [0,\Delta ]. \end{aligned}$$

Now, note that, for $t \ge T^* > t_U$, the matrix $M(t;\epsilon )$ and hence the matrix $e^{M(t;\epsilon )}$ is non-increasing. Thus, as $e^{M(t;\epsilon )}$ is non-negative (as proved in Proposition 5), it is necessary from Perron–Frobenius theory (Berman and Plemmons 1994) that its Perron eigenvalue, $\lambda (t;\epsilon )$ satisfies

A88 $$\begin{aligned} \lambda (t;\epsilon ) \le \lambda (T^*;\epsilon )< e^{-\kappa } < 1. \end{aligned}$$

Then, following the method used to derive (A67), one has, for any $t \ge T^*$

A89 $$\begin{aligned} \int _t^{\infty }I_1(t;\epsilon ){\textrm{d}}t \le ({\varvec{M}}(t;\epsilon )^{-1}{\varvec{J}}(t;\epsilon ))_1 \quad \forall \epsilon \in [0,\Delta ]. \end{aligned}$$

This is exactly the same equation as (A67), except that here, $T^*$ is independent of $\delta$ (as no conditions on ${\varvec{J}}(T;0)$ are assumed). Now, note that

A90 $$\begin{aligned} M(t;0)_{1j} = -\mu _1^1\delta _{1j} \quad \text {and} \quad M(t;0)_{(n+1)j} = -\mu _1^2 \delta _{(n+1),j} \end{aligned}$$

where here $\delta _{ij}$ is the Kronecker delta. This means that, for any vector ${\varvec{y}}$, the equation

A91 $$\begin{aligned} {\varvec{M}}(t;0){\varvec{x}} = {\varvec{y}} \end{aligned}$$

must satisfy

A92 $$\begin{aligned} x_1 = \frac{-y_1}{\mu _1^1} \quad x_{n+1} = -\frac{y_{n+1}}{\mu _1^2} \quad \text {and} \quad {\varvec{x}} = {\varvec{M}}^{-1}{\varvec{y}}. \end{aligned}$$

Thus, in particular

A93 $$\begin{aligned} {\varvec{M}}^{-1}_{1j}(t;0) = \frac{-1}{\mu _1^1}\delta _{1j} \quad \text {and} \quad {\varvec{M}}^{-1}_{(n+1)j}(t;0) = \frac{-1}{\mu _1^2}\delta _{(n+1)j}, \end{aligned}$$

where here $\delta _{ij}$ denotes the Kronecker delta. Now, note that, as the inverse of a matrix is a rational function of its entries,

A94 $$\begin{aligned} {\varvec{M}}^{-1}(t;0) = {\varvec{M}}^{-1}(t;\epsilon ) + O(\epsilon ) \end{aligned}$$

and hence

A95 $$\begin{aligned} {\varvec{M}}^{-1}_{1j}(t;0) = \frac{-1}{\mu _1^1}\delta _{1j} + O(\epsilon ). \end{aligned}$$

Moreover, defining

A96 $$\begin{aligned} \mu _{\min } := \min \{\mu ^1_i,\mu _i^2\} , \end{aligned}$$

there must exist a $T(\epsilon ) \in \bigg (T^*, T^* + \frac{2n}{\delta ^{\frac{1}{3}}\mu _{\min }}\bigg )$ such that for each *i*,

A97 $$\begin{aligned} I_i(T(\epsilon );\epsilon ) < \delta ^{\frac{1}{3}} N_i(\epsilon ). \end{aligned}$$

Otherwise,

A98 $$\begin{aligned} \sum _{i=1}^n\frac{{\textrm{d}}}{{\textrm{d}}t}\left( \frac{R_i(t;\epsilon )}{\mu _i^1 N_i(\epsilon )} + \frac{R^V_i(t;\epsilon )}{\mu _i^2N_i(\epsilon )}\right)&\ge \sum _{i=1}^n\left( \frac{\mu _i^1 I_i(t;\epsilon )}{\mu _i^1 N_i(\epsilon )} + 0\right) \ge \delta ^{\frac{1}{3}} \nonumber \\ {}&\quad \forall t \in \bigg (T^*, T^* + \frac{2n}{\delta ^{\frac{1}{3}}\mu _{\min }}\bigg ) \end{aligned}$$

and integrating this between $T^*$ and $T^* + \frac{2n}{\delta ^{\frac{1}{3}}\mu _{\min }}$ gives

A99 $$\begin{aligned} \sum _{i=1}^n\left[ \frac{R_i\bigg (T^* + \frac{2n}{\delta ^{\frac{1}{3}}\mu _{\min }};\epsilon \bigg )}{\mu _i^1 N_i} + \frac{R^V_i\bigg (T^* + \frac{2n}{\delta ^{\frac{1}{3}}\mu _{\min }};\epsilon \bigg )}{\mu _i^2 N_i(\epsilon )}\right] \ge \frac{2n\delta ^{\frac{1}{3}}}{\delta ^{\frac{1}{3}}\mu _{\min }} > \frac{n}{\mu _{\min }}. \quad \end{aligned}$$

Thus, as $\frac{\mu _{\min }}{\mu _i^{\alpha }} \le 1$ for each *i* and $\alpha$,

A100 $$\begin{aligned} \sum _{i=1}^n\left[ \frac{R_i\bigg (T^* + \frac{2n}{\delta ^{\frac{1}{3}}(\mu _{\min } + 1)};0\bigg ) +R^V_i\bigg (T^* + \frac{2n}{\delta ^{\frac{1}{3}}(\mu _{\min } + 1)};0\bigg ) }{N_i(\epsilon )}\right] > n \end{aligned}$$

which means, for some *i*

A101 $$\begin{aligned} \frac{R_i\bigg (T^* + \frac{2n}{\delta ^{\frac{1}{3}}(\mu _{\min } + 1)};0\bigg )+R^V_i\bigg (T^* + \frac{2n}{\delta ^{\frac{1}{3}}(\mu _{\min } + 1)};0\bigg )}{N_i(\epsilon )} > 1 , \end{aligned}$$

which is a contradiction as the total population size in group *i* cannot exceed $N_i(\epsilon )$ by definition of $N_i(\epsilon )$. Thus, for each $\epsilon \in [0,\Delta ]$,

A102 $$\begin{aligned} \int _{T(\epsilon )}^{\infty } I_1(t;\epsilon ) {\textrm{d}}t&\le ({\varvec{M}}(T;\epsilon )^{-1}{\varvec{J}}(T(\epsilon );\epsilon ))_1 \end{aligned}$$

A103 $$\begin{aligned}&=\begin{pmatrix} O(1)&O(\epsilon )&...&O(\epsilon ) \end{pmatrix}\begin{pmatrix} O(\epsilon \delta ^{\frac{1}{3}}) \\ O(\delta ^{\frac{1}{3}}) \\ .\\ .\\ .\\ O(\delta ^{\frac{1}{3}})\end{pmatrix}\end{aligned}$$

A104 $$\begin{aligned}&=O(\epsilon \delta ^{\frac{1}{3}}) \end{aligned}$$

while similarly

A105 $$\begin{aligned} \int _{T(\epsilon )}^{\infty } I^V_1(t;\epsilon ) {\textrm{d}}t = O(\epsilon \delta ^{\frac{1}{3}}). \end{aligned}$$

Moreover,

A106 $$\begin{aligned} \int _0^{T(\epsilon )}\delta \epsilon {\textrm{d}}t = O(\epsilon \delta ^{\frac{2}{3}}). \end{aligned}$$

These results allow for the linearisation to be carried out. To reduce notation, define

A107 $$\begin{aligned} T := T(\epsilon ). \end{aligned}$$

Now, to begin the linearisation, define

A108 $$\begin{aligned} X(t) = \sum _{j=1}^n\bigg [ \beta ^1_{1j}I_j(t;0) + \beta _{1j}^2 I_j^V(t;0)\bigg ], \end{aligned}$$

which is the leading-order infective force on group 1. By Proposition 5,

A109 $$\begin{aligned} X(t) = \sum _{j=1}^n\bigg [ \beta ^1_{1j}I_j(t;\epsilon ) + \beta _{1j}^2 I_j^V(t;\epsilon )\bigg ] + O(\delta ). \end{aligned}$$

Then, as $$S_1(t;\epsilon ) \le \epsilon $$,

A110 $$\begin{aligned} \frac{{\textrm{d}}I_1}{{\textrm{d}}t}(t;\epsilon ) + \mu _1^1 I_1(t) = S_1(t;\epsilon )X(t) + O(\delta \epsilon ). \end{aligned}$$

Now, note that

A111 $$\begin{aligned} R_1(\infty ;\epsilon )&= \mu _1^1\int _0^{\infty }I_1(t;\epsilon ){\textrm{d}}t \end{aligned}$$

A112 $$\begin{aligned}&= \mu ^1_1\int _0^{T}I_1(t;\epsilon ){\textrm{d}}t + \mu _1^1\int _{T}^{\infty } I_1(t;\epsilon ){\textrm{d}}t\end{aligned}$$

A113 $$\begin{aligned}&= \int _0^{T} \bigg (S_1(t;\epsilon )X(t) - \frac{{\textrm{d}}I_1}{{\textrm{d}}t}(t;\epsilon ) + O(\epsilon \delta )\bigg ){\textrm{d}}t +O(\delta ^{\frac{1}{3}} \epsilon )\end{aligned}$$

A114 $$\begin{aligned}&=I_1(0;\epsilon ) -I_1(T)+ \int _0^{T} S_1(t;\epsilon )X(t){\textrm{d}}t+O(\delta ^{\frac{1}{3}} \epsilon )\end{aligned}$$

A115 $$\begin{aligned}&= I_1(0;\epsilon )+ \int _0^{T} S_1(t;\epsilon )X(t){\textrm{d}}t+O(\delta ^{\frac{1}{3}}\epsilon ). \end{aligned}$$

Now, the equations for $I^V$ are of the same form, but with $S^V$ in place of *S* and a different leading-order infection function *Y*(*t*) given by

A116 $$\begin{aligned} Y(t) = \sum _{j=1}^n\bigg [\beta _{1j}^3I_j(t;0) + \beta _{ij}^4 I^V_j(t;0)\bigg ] . \end{aligned}$$

Thus, an analogous derivation (noting that $I^V(0;\epsilon ) = 0$) shows that

A117 $$\begin{aligned} R^V_1(\infty ;0) =\int _0^{T}Y(t)S^V_1(t;\epsilon ){\textrm{d}}t + O(\epsilon \delta ^{\frac{1}{3}}) \end{aligned}$$

while analogous results hold for ${\tilde{R}}_1$ and ${\tilde{R}}^V_1$ (with $\tilde{S_1}$ and ${\tilde{S}}^V_1$ in place of $S_1$ and $S^V_1$). Now, note that

A118 $$\begin{aligned} S_1(t;\epsilon )&= S_1(t;\epsilon )\left( \frac{N_1(\epsilon ) - W_1(t;\epsilon )}{N_1(\epsilon )}\right) \exp \left[ -\sum _{j=1}^n\left( \frac{\beta ^1_{1j}R_j(t;\epsilon )}{\mu ^1_j} + \frac{\beta ^2_{1j}R^V_j(t;\epsilon )}{\mu ^2_j}\right) \right] \end{aligned}$$

A119 $$\begin{aligned}&= \sigma (N_1(\epsilon ) - W_1(t;\epsilon ))\exp \left[ -\sum _{j=1}^n\left( \frac{\beta ^1_{1j}R_j(t;\epsilon )}{\mu ^1_j} + \frac{\beta ^2_{1j}R^V_j(t;\epsilon )}{\mu ^2_j}\right) \right] . \end{aligned}$$

Define

A120 $$\begin{aligned} P(t) := \exp \left[ -\sum _{j=1}^n\left( \frac{\beta ^1_{1j}R_j(t;0)}{\mu ^1_j} + \frac{\beta ^2_{1j}R^V_j(t;0)}{\mu ^2_j}\right) \right] \end{aligned}$$

and then, note that by Proposition 5

A121 $$\begin{aligned} P(t) = \exp \left[ -\sum _{j=1}^n\left( \frac{\beta ^1_{1j}R_j(t;\epsilon )}{\mu ^1_j} + \frac{\beta ^2_{1j}R^V_j(t;\epsilon )}{\mu ^2_j}\right) \right] + O(\delta ) \end{aligned}$$

which means (as $(N_1(\epsilon ) - W_1(t;\epsilon )) \le \epsilon$ and $\sigma < 1$)

A122 $$\begin{aligned} S_1(t;\epsilon ) = \sigma (N_1 - W_1(t;\epsilon ))P(t) +O(\delta \epsilon ) \end{aligned}$$

with an identical result for ${\tilde{S}}$. It is helpful to note for later that, as $W_1(t;\epsilon ) \le {\tilde{W}}(t;\epsilon )$, this means that

A123 $$\begin{aligned} S_1(t;\epsilon ) \ge {\tilde{S}}_1(t;\epsilon ) + O(\delta \epsilon ). \end{aligned}$$

Now, this means

A124 $$\begin{aligned} \int _0^{T}X(t)S_1(t;\epsilon ){\textrm{d}}t =\int _0^TX(t)\sigma (N_1 - W_1(t;\epsilon ))P(t){\textrm{d}}t + O(\epsilon \delta ^{\frac{2}{3}}) \end{aligned}$$

and so

A125 $$\begin{aligned} R_1(\infty ;\epsilon )= I_1(0;\epsilon ) + \int _0^{T}X(t)\sigma (N_1 - W_1(t;\epsilon ))P(t){\textrm{d}}t+ O(\epsilon \delta ^{\frac{1}{3}}). \end{aligned}$$

Now, note that

A126 $$\begin{aligned} \int _0^{T}X(t)\sigma (N_1 - W_1(t;\epsilon ))P(t){\textrm{d}}t&= \left( \int _0^{\tau } + \int _{\tau }^{T}\right) \bigg (X(t)\sigma (N_1 - W_1(t;\epsilon ))P(t){\textrm{d}}t \bigg )\nonumber \\ \end{aligned}$$

and that, as $W_1(t;\epsilon ) \le {\tilde{W}}_1(t;\epsilon )$,

A127 $$\begin{aligned} \int _{\tau }^{T}X(t)\sigma (N_1 - W_1(t;\epsilon ))P(t){\textrm{d}}t \ge \int _{\tau }^{T}X(t)\sigma (N_1 - {\tilde{W}}_1(t;\epsilon ))P(t){\textrm{d}}t.\qquad \end{aligned}$$

Now, define $z(\epsilon )$ to be

A128 $$\begin{aligned} z(\epsilon ) = \inf \left\{ t : \sum _{i=1}^n W_i(t) = \epsilon \right\} . \end{aligned}$$

Note that, for $\epsilon < w$, *z* exists and is bounded above by $\tau$ as

A129 $$\begin{aligned} \sum _{i=1}^n W_i(\tau ) = w. \end{aligned}$$

Now, define a fixed value

A130 $$\begin{aligned} z_0 := z\left( \frac{w}{2}\right) \end{aligned}$$

so that, by continuity of *W*, $z_0 < \tau$ (and is independent of $\epsilon$). Suppose that $\epsilon < \frac{w}{2}$ (which will be assumed for the rest of the proof). Note that

A131 $$\begin{aligned} \int _{0}^{z_0}X(t)\sigma (N_1 - W_1(t;\epsilon ))P(t){\textrm{d}}t \ge \int _{0}^{z_0}X(t)\sigma (N_1 - {\tilde{W}}_1(t;\epsilon ))P(t){\textrm{d}}t \end{aligned}$$

and that

A132 $$\begin{aligned} \int _{z_0}^{\tau }X(t)\sigma (N_1 - {\tilde{W}}_1(t;\epsilon ))P(t){\textrm{d}}t = 0 \end{aligned}$$

as ${\tilde{W}}_1(t;\epsilon ) = N_1$ for all $t > z(\epsilon )$. Moreover, by (A2)

A133 $$\begin{aligned} \int _{z_0}^{\tau }X(t)\sigma (N_1 - W_1(t;\epsilon ))P(t){\textrm{d}}t \ge (1-\alpha ) \epsilon \sigma \int _{z_0}^{\tau }X(t) P(t){\textrm{d}}t . \end{aligned}$$

Now, note that *P*(*t*) is strictly positive for $t > 0$ as it is an exponential, while, as $\beta _{1j} > 0$ for some $j\ne 1$,

A134 $$\begin{aligned} X(t) \ge \beta _{ij}I_j(t;0) > 0 \quad \text {as } j \in \Pi (0) . \end{aligned}$$

Thus,

A135 $$\begin{aligned} (1-\alpha ) \int _{z_0}^{\tau }X(t) P(t){\textrm{d}}t > 0 \end{aligned}$$

and this is independent of $\epsilon$. This means that

A136 $$\begin{aligned}{} & {} R_1(\infty ;\epsilon ) - {\tilde{R}}(\infty ;\epsilon ) \ge \epsilon (1-\alpha ) \int _{z}^{\tau }X(t) P(t){\textrm{d}}t + O(\epsilon \delta ^{\frac{1}{3}}) \nonumber \\{} & {} \quad = \epsilon (1-\alpha ) \int _{z}^{\tau }X(t) P(t){\textrm{d}}t + o(\epsilon ) \end{aligned}$$

and so the leading-order change in $R_1(\infty ;\epsilon )$ is indeed of order exactly $\epsilon$.

Now, it is important to check the leading-order change in $R^V_1(\infty ;\epsilon )$. Note that, as $S_1(t;\epsilon )$ and $S^V_1(\epsilon )$ are at most $\epsilon$,

A137 $$\begin{aligned} \frac{{\textrm{d}}}{{\textrm{d}}t}\left( S_1(t;\epsilon ) + S^V_1(t;\epsilon )\right)&= -X(t)S_1(t;\epsilon ) - Y(t)S_1^V(t;\epsilon ) +O(\epsilon \delta ). \end{aligned}$$

Using (A122), this can be written as:

A138 $$\begin{aligned} \frac{{\textrm{d}}}{{\textrm{d}}t}\left( S_1(t;\epsilon ) + S^V_1(t;\epsilon )\right) + Y(t)(S_1(t;\epsilon ) + S^V_1(t;\epsilon ))&= (Y(t) - X(t))S_1(t;\epsilon ) + O(\epsilon \delta ). \end{aligned}$$

This equation can be integrated by defining

A139 $$\begin{aligned} {\mathcal {Y}}(t) := \int _0^t Y(s){\textrm{d}}s \end{aligned}$$

so that

A140 $$\begin{aligned} \frac{{\textrm{d}}}{{\textrm{d}}t}\left( e^{{\mathcal {Y}}(t)} (S_1(t;\epsilon ) + S^V_1(t;\epsilon )\right) = e^{{\mathcal {Y}}(t)} (Y(t) - X(t))S_1(t;\epsilon ) + O(\epsilon \delta ).\qquad \end{aligned}$$

Thus, for any $t \le T$

A141 $$\begin{aligned} S_1(t;\epsilon ) + S_1^V(t;\epsilon ) =&e^{-{\mathcal {Y}}(t)}(S_1(0;\epsilon ) + S_1^V(0;\epsilon ))\nonumber \\&+ \int _0^te^{{\mathcal {Y}}(s) - {\mathcal {Y}}(t)} (Y(s) - X(s))S_1(s;\epsilon ){\textrm{d}}s + O(\epsilon \delta ^{\frac{2}{3}}) \end{aligned}$$

which means that

A142 $$\begin{aligned}&S_1(t;\epsilon ) + S_1^V(t;\epsilon ) - {\tilde{S}}_1(t;\epsilon ) - {\tilde{S}}^V_1(t;\epsilon ) \nonumber \\&=\int _0^te^{{\mathcal {Y}}(s) - {\mathcal {Y}}(t)} (Y(s) - X(s))\bigg (S_1(s;\epsilon ) - {\tilde{S}}_1(s;\epsilon )\bigg ){\textrm{d}}s + O(\epsilon \delta ^{\frac{2}{3}}) \end{aligned}$$

Thus,

A143 $$\begin{aligned}&\int _0^tY(s)\bigg [S_1(s;\epsilon ) + S_1^V(s;\epsilon ) - {\tilde{S}}_1(s;\epsilon ) - {\tilde{S}}^V_1(s;\epsilon )\bigg ]{\textrm{d}}s \end{aligned}$$

A144 $$\begin{aligned}&\quad =\int _0^t\int _0^sY(s)e^{{\mathcal {Y}}(k) - {\mathcal {Y}}(s)}(Y(k) - X(k)) \bigg (S_1(k;\epsilon ) - {\tilde{S}}_1(k;\epsilon )\bigg ){\textrm{d}}k{\textrm{d}}s +O(\epsilon \delta ^{\frac{1}{3}})\end{aligned}$$

A145 $$\begin{aligned}&\quad = \int _0^t\int _{k}^t\bigg [Y(s)e^{-{\mathcal {Y}}(s)}\bigg ] e^{{\mathcal {Y}}(k)}(Y(k) - X(k)) \bigg (S_1(k;\epsilon ) - {\tilde{S}}_1(k;\epsilon )\bigg ){\textrm{d}}s{\textrm{d}}k +O(\epsilon \delta ^{\frac{1}{3}})\end{aligned}$$

A146 $$\begin{aligned}&\quad = \int _0^t(e^{-{\mathcal {Y}}(k)} - e^{-{\mathcal {Y}}(t)})e^{{\mathcal {Y}}(k)}(Y(k) - X(k)) \bigg (S_1(k;\epsilon ) - {\tilde{S}}_1(k;\epsilon )\bigg ){\textrm{d}}k +O(\epsilon \delta ^{\frac{1}{3}})\end{aligned}$$

A147 $$\begin{aligned}&\quad = \int _0^t(1 - e^{{\mathcal {Y}}(k)-{\mathcal {Y}}(t)})(Y(k) - X(k)) \bigg (S_1(k;\epsilon ) - {\tilde{S}}_1(k;\epsilon )\bigg ){\textrm{d}}k +O(\epsilon \delta ^{\frac{1}{3}}). \end{aligned}$$

Now, note that, as ${\mathcal {Y}}$ is non-decreasing, and non-negative

A148 $$\begin{aligned} 0 \le 1 - e^{{\mathcal {Y}}(k)-{\mathcal {Y}}(t)} \le 1 - e^{-{\mathcal {Y}}(t)}. \end{aligned}$$

Moreover, one has

A149 $$\begin{aligned} {\mathcal {Y}}(t)&= \int _0^t \sum _{j=1}^n\bigg [\beta _{1j}^3I_j(s;0) + \beta ^4_{1j}I^V_j(s;0)\bigg ] {\textrm{d}}s\end{aligned}$$

A150 $$\begin{aligned}&= \sum _{j=1}^n\bigg [\frac{\beta ^3_{1j}R_j(t;0)}{\mu _j^1} + \frac{\beta ^4_{1j}R_j(t;0)}{\mu _j^2}\bigg ]\end{aligned}$$

A151 $$\begin{aligned}&\le \sum _{j=1}^n\bigg [\frac{\beta ^3_{1j}N_j(1)}{\mu _j^1} + \frac{\beta ^4_{1j}N_j(1)}{\mu _j^2}\bigg ] \end{aligned}$$

and so ${\mathcal {Y}}(t)$ is bounded above by some constant (for $\epsilon \le 1$). This in turn means that there exists some ${\mathcal {Y}}^*$ such that

A152 $$\begin{aligned} 1 - e^{-{\mathcal {Y}}(t)} \le {\mathcal {Y}}^* < 1. \end{aligned}$$

Thus, as $Y(t) - X(t) \le 0$ and $S_1(k;\epsilon ) \ge {\tilde{S}}_1(k;\epsilon ) + O(\delta \epsilon )$, for any $k \le t$

A153 $$\begin{aligned}&\int _0^tY(s)\bigg [S_1(s;\epsilon ) + S_1^V(s;\epsilon ) - {\tilde{S}}_1(s;\epsilon ) - {\tilde{S}}^V_1(s;\epsilon )\bigg ]\nonumber \\&\quad \ge {\mathcal {Y}}^*\int _0^t(Y(k) - X(k))\bigg (S_1(k;\epsilon ) - {\tilde{S}}_1(k;\epsilon )\bigg ){\textrm{d}}k +O(\epsilon \delta ^{\frac{1}{3}}). \end{aligned}$$

Now, adding inequalities (A115) and (A117) together gives

A154 $$\begin{aligned} R_1(\infty ;\epsilon ) + R^V_1(\infty ;\epsilon ) =I_1(0;\epsilon ) + \int _0^{T}X(t)S_1(t;\epsilon ) + Y(t)S^V_1(t;\epsilon ) {\textrm{d}}t+o(\epsilon ).\qquad \end{aligned}$$

Note that

A155 $$\begin{aligned} X(t)S_1(t;\epsilon ) + Y(t)S^V_1(t;\epsilon ) = (X(t)-Y(t))S_1(t;\epsilon ) +Y(t)(S_1(t;\epsilon ) + S^V_1(t;\epsilon )) \end{aligned}$$

and hence

A156 $$\begin{aligned}&R_1(\infty ;\epsilon ) + R^V_1(\infty ;\epsilon ) = I_1(0;\epsilon ) +\nonumber \\&\quad \int _0^{T} (X(t)-Y(t))S_1(t;\epsilon ) + Y(t)(S_1(t;\epsilon ) + S^V_1(t;\epsilon )) {\textrm{d}}t+o(\epsilon ). \end{aligned}$$

This means that

A157 $$\begin{aligned}&R_1(\infty ;\epsilon ) + R^V_1(\infty ;\epsilon ) - {\tilde{R}}_1(\infty ;\epsilon ) - {\tilde{R}}^V_1(\infty ;\epsilon ) \nonumber \\&\quad \ge (1-{\mathcal {Y}}^*)\int _0^T(X(t) - Y(t))\bigg (S_1(t;\epsilon ) - {\tilde{S}}_1(t;\epsilon )\bigg ){\textrm{d}}t +O(\epsilon \delta ^{\frac{1}{3}}). \end{aligned}$$

Now, as there is some $i \ne 1$ such that

A158 $$\begin{aligned} \beta ^1_{1i} > \beta ^3_{1i} \ge 0 \end{aligned}$$

and (as $i\ne 1$), $i \in \Pi (0)$ which means that

A159 $$\begin{aligned} \beta ^1_{1i}I_i(t)> \beta ^3_{1i}I_i(t) \quad \forall t > 0. \end{aligned}$$

This means that $X(t) > Y(t)$ for all $t > 0$ and hence

A160 $$\begin{aligned} \int _0^T(X(t) - Y(t)){\textrm{d}}t > 0. \end{aligned}$$

Thus, following the arguments from before, one can see that

A161 $$\begin{aligned} \int _0^t(X(s) - Y(s))\bigg (S_1(s;\epsilon ) - {\tilde{S}}_1(s;\epsilon )\bigg ){\textrm{d}}s > \epsilon (1-{\mathcal {Y}}^*) \int _{z_0}^{\tau }(X(t) - Y(t))P(t){\textrm{d}}t + o(\epsilon )\nonumber \\ \end{aligned}$$

where the leading-order term is positive as required (as *P*(*t*) is positive). Hence, from (A157)

A162 $$\begin{aligned}&R_1(\infty ;\epsilon ) + R^V_1(\infty ;\epsilon ) - ({\tilde{R}}_1(\infty ;\epsilon )+ {\tilde{R}}^V_1(\infty ;\epsilon ))\nonumber \\&\quad \ge (1-{\mathcal {Y}}^*)\epsilon (1-\alpha )\int _{z_0}^{\tau }(X(t)-Y(t))P(t){\textrm{d}}t + o(\epsilon ). \end{aligned}$$

Thus, for any $\kappa _1 \in [0,1]$, combining (A136) and (A162)

A163 $$\begin{aligned}&R_1(\infty ) + \kappa _1 R^V_1(\infty ) = \kappa _1(R_1(\infty ) + R^V_1(\infty )) + (1-\kappa _1)R_1(\infty )\nonumber \\&\quad \ge \epsilon \int _{z_0}^{\tau } (1-\alpha )P(t)\bigg [(1-{\mathcal {Y}}^*)\kappa _1(X(t) - Y(t)) + (1-\kappa _1) X(t)\bigg ]{\textrm{d}}t \end{aligned}$$

A164 $$\begin{aligned}&\qquad + \kappa _1{\tilde{R}}^V_1(\infty ) + {\tilde{R}}_1(\infty ) + o(\epsilon ). \end{aligned}$$

Thus, recalling (A85) and that $p_1 = \frac{1}{\epsilon }$

A165 $$\begin{aligned} H({\varvec{U}}) \ge H(\tilde{{\varvec{U}}}) +\int _{z_0}^{\tau } (1-\alpha )[\kappa _1(X(t) - Y(t)) + (1-\kappa _1) X(t)]{\textrm{d}}t + o(1) \end{aligned}$$

for some constant *K*. Moreover, for sufficiently small $\epsilon$,

A166 $$\begin{aligned} \int _{z_0}^{\tau } \alpha [\kappa _1(X(t) - Y(t)) + (1-\kappa _1) X(t)]{\textrm{d}}t +o(1) > 0 \end{aligned}$$

and hence

A167 $$\begin{aligned} H({\varvec{U}}(t;\epsilon )) > H(\tilde{{\varvec{U}}}(t;\epsilon )) , \end{aligned}$$

as required.

## Appendix B Proof of Theorem 2

Note that, throughout this section, the tilde will be removed from the rescaled $p_i$ terms to reduce notation (and hence all $p_i$ terms used here will be assumed to be rescaled).

Recall from the main text that, using the results in Penn and Donnelly (2022), if one defines

B168 $$\begin{aligned} \chi (t) := \left\{ \begin{matrix} A(t) &{} \text {if} \quad \int _0^tA(s){\textrm{d}}s < B(t) \\ \min (A(t), B'(t)) &{} \text {if} \quad \int _0^tA(s){\textrm{d}}s \ge B(t) \end{matrix}\right. , \end{aligned}$$

then (assuming that there is an optimal solution, and under mild smoothness conditions on ${\varvec{U}}$, *A* and *B*) there must be an optimal solution satisfying

B169 $$\begin{aligned} \sum _{i=1}^nW_i(t) =\max \bigg (\int _0^t \chi (s){\textrm{d}}s,1\bigg ). \end{aligned}$$

### Theorem 2

With the definitions of Theorem 1, suppose additionally that

B170 $$\begin{aligned} \sum _{j=2}^n (\beta _{1j}^1 - \beta _{1j}^3)I_j(0;\epsilon ) > 0. \end{aligned}$$

That is, the initial difference between the infective force on vaccinated and unvaccinated members of the population is positive. Suppose further that

B171 $$\begin{aligned} \sigma = 1. \end{aligned}$$

Suppose an optimal vaccination policy for each $\epsilon$ is given by $\overline{{\varvec{U}}}(t;\epsilon )$ and suppose that $\overline{{\varvec{U}}}(t;\epsilon )$ has uniformly bounded finite support. Then, there exists an $\eta$ depending only on $\alpha$, $\tau$, *w* and the model parameters such that, for any ${\varvec{U}}$ satisfying the condition (A2) as defined in Theorem 1

B172 $$\begin{aligned} \epsilon \in (0,\eta ) \Rightarrow H({\varvec{U}}(t;\epsilon )) > H(\overline{{\varvec{U}}}(t;\epsilon )). \end{aligned}$$

Moreover, there is a sequence of optimal vaccination policies $\overline{{\varvec{U}}}(t;\epsilon )$ satisfying

B173 $$\begin{aligned} \lim _{\epsilon \rightarrow 0}\left( \frac{{\overline{W}}_1(t;\epsilon )}{\epsilon }\right) = 1 \quad \forall t \quad \text {s.t.}\quad \int _0^t \chi (s){\textrm{d}}s > 0. \end{aligned}$$

To make things clearer in the course of this proof, note that *H* will be written as

B174 $$\begin{aligned} H({\varvec{U}};\epsilon ) \end{aligned}$$

where the $\epsilon$ refers to the size of the population $N_1$ under consideration.

### Proposition 6

It remains to show that, for sufficiently small $\epsilon$ and fixed $\alpha$, $\tau$ and *w*, there is no ${\varvec{U}}$ satisfying the conditions (A2) that is the optimal vaccination policy.

To do this, it is necessary to prove the function $H({\varvec{U}}(t;\epsilon );\epsilon )$ is non-increasing in $\epsilon$. This uses the work of Penn and Donnelly (2022) as the main result of that paper gives a method of finding inequalities between the objective values of different vaccination policies. However, it is a meaningful extension, as here the population sizes are not identical when objective values are compared.

#### Proposition 6

Suppose that $I_1(0;\epsilon ) = 0$ for all $\epsilon$. Consider, for $\epsilon \le 1$ any bounded vaccination policy ${\varvec{U}}(t;\epsilon )$ given by

B175 $$\begin{aligned} U_1(t;\epsilon ) = \left\{ \begin{matrix} U_1(t;1) &{} \text {if } W_1(t;1) < \epsilon \\ 0 &{} \text {otherwise} \end{matrix}\right. \quad \text {and} \quad U_i(t;\epsilon ) = U_i(t;1) \quad \forall i \ne 1. \end{aligned}$$

Then, if $H({\varvec{U}}(t;\epsilon );\epsilon )$ is the value of the objective function for a given value of $\epsilon$,

B176 $$\begin{aligned} \epsilon > \epsilon ' \Rightarrow H({\varvec{U}}(t;\epsilon );\epsilon ) \ge H({\varvec{U}}(t;\epsilon ');\epsilon '). \end{aligned}$$

#### Proof

Fix $\epsilon$ and $\epsilon '$ such that $\epsilon > \epsilon '$. Define the vaccination policy ${\varvec{U}}^*(t,\Delta ;\epsilon )$ to be

B177 $$\begin{aligned} U^*_1(t,\Delta ;\epsilon ) = \left\{ \begin{array}{cc} \frac{(\epsilon - \epsilon ^\prime + W_1(\Delta ;\epsilon ^\prime ))}{\Delta } &{} \text {if }t < \Delta \\ U_1(t;\epsilon ^\prime ) &{} \text {otherwise} \end{array}\right. \quad \text {and} \quad U^*_i(t,\Delta ;\epsilon ) = U_i(t;1) \quad \forall i \ne 1.\nonumber \\ \end{aligned}$$

Then, in particular

B178 $$\begin{aligned} W^*_1(t,\Delta ;\epsilon ) =\frac{ t(\epsilon - \epsilon ' + W_1(\Delta ;\epsilon '))}{\Delta } \quad \forall t < \Delta . \end{aligned}$$

Now, as ${\varvec{U}}(t;\epsilon )$ is bounded by some *M*, it is necessary that $W_1(t;\epsilon )$ is bounded above by *tM*. Conversely, $W_1^*(t;\epsilon )$ is bounded below by $\frac{(\epsilon - \epsilon ')t}{\Delta }$ for $t < \Delta$. Thus, taking $\Delta$ sufficiently small gives

B179 $$\begin{aligned} W^*_1(t,\Delta ;\epsilon )>W_1(t;\epsilon ) \quad \forall t < \Delta . \end{aligned}$$

Moreover, note that, assuming $\Delta < \epsilon '$, if $t > \Delta$ is chosen such that $W_1(t;1)< \epsilon ' < \epsilon$, then

B180 $$\begin{aligned} U_1^*(t,\Delta ;\epsilon ) = U_1(t;\epsilon ') = U_1(t;\epsilon ) = U_1(t;1) \end{aligned}$$

and hence

B181 $$\begin{aligned} W^*_1(t,\Delta ;\epsilon )>W_1(t;\epsilon ) \quad \forall t \quad \text {s.t.} \quad W_1(t;1) < \epsilon '. \end{aligned}$$

Finally, note that if $W_1(t;1) \ge \epsilon '$ then $W_1(t;\epsilon ') = \epsilon '$ and hence

B182 $$\begin{aligned} W^*_1(t,\Delta ;\epsilon )&= W^*_1(\Delta ;\Delta ) + \int _{\Delta }^tU_1(s;\epsilon '){\textrm{d}}s \end{aligned}$$

B183 $$\begin{aligned}&= \epsilon - \epsilon ' + W_1(\Delta ;\epsilon ') + \int _{\Delta }^tU_1(s;\epsilon '){\textrm{d}}s\end{aligned}$$

B184 $$\begin{aligned}&= \epsilon - \epsilon ' + W_1(\Delta ;\epsilon ') + W_1(t;\epsilon ') - W_1(\Delta ;\epsilon ')\end{aligned}$$

B185 $$\begin{aligned}&= \epsilon \end{aligned}$$

and so

B186 $$\begin{aligned} W^*_1(t,\Delta ;\epsilon ) = \epsilon \ge W_1(t;\epsilon ) \quad \forall t \ge 0. \end{aligned}$$

Moreover,

B187 $$\begin{aligned} W^*_i(t,\Delta ;\epsilon )= W_i(t;\epsilon ) \quad \forall t \ge 0 \quad \text {and} \quad \forall i \in \{2,\ldots ,n\}. \end{aligned}$$

Thus, in particular, by Theorem 17, proved in Penn and Donnelly (2022), for each $i \in \{1,\ldots ,n\}$,

B188 $$\begin{aligned}&I_i^*(t,\Delta ;\epsilon ) + R_i^*(t;\Delta ;\epsilon ) + I_i^{V^*}(t,\Delta ;\epsilon ) + R_i^{V^*}(t,\Delta ;\epsilon )\nonumber \\&\quad \le I_i(t;\epsilon ) + R_i(t;\epsilon ) + I^V_i(t;\epsilon ) + R^V_i(t;\epsilon ) \end{aligned}$$

and

B189 $$\begin{aligned} R^*_i(t;\Delta ;\epsilon ) \le R_i(t;\epsilon ) \end{aligned}$$

where the $f^*_i(t,\Delta ;\epsilon )$ are the values of the model variables under the ${\varvec{U}}^*(t,\Delta ;\epsilon )$ vaccination policy and the $f_i(t;\epsilon )$ are their values under the ${\varvec{U}}(t;\epsilon )$ vaccination policy. $\square$

Now, for all $\Delta > 0$ and all *f* and *i*

B190 $$\begin{aligned} f^*_i(0,\Delta ;\epsilon ) = f_i(0;\epsilon ) \end{aligned}$$

so, as all model variables except $S_i$ and $S^V_i$ have derivatives that are bounded independently , there exists some *L* such that, for all $f \in \{I_i(t,\Delta ;\epsilon ),I^V_i(t,\Delta ;\epsilon ),R_i(t,\Delta ;\epsilon ),R^V_i(t,\Delta ;\epsilon )\}$,

B191 $$\begin{aligned} |f^*_i(\Delta ;\Delta ;\epsilon ) - f_i(0;\epsilon )|= |f^*_i(\Delta ;\Delta ) - f^*_i(0;\Delta )|< L\Delta . \end{aligned}$$

Moroever, the initial conditions are the same for $f(0;\epsilon )$ and $f(0;\epsilon ')$ except in the case of $S_1(0;\epsilon )$. Thus,

B192 $$\begin{aligned} |f^*_i(\Delta ;\Delta ;\epsilon ) - f_i(0;\epsilon ')|< L\Delta \quad \forall f \in \{I_i(t,\Delta ;\epsilon ),I^V_i(t,\Delta ;\epsilon ),R_i(t,\Delta ;\epsilon ),R^V_i(t,\Delta ;\epsilon )\}.\nonumber \\ \end{aligned}$$

As only the $W_1$ policy has an unbounded derivative in the $\Delta \rightarrow 0$ limit, it is also true that

B193 $$\begin{aligned} |f^*_i(\Delta ;\Delta ;\epsilon ) - f_i(0;\epsilon ')|< L\Delta \quad \forall f \in \{S_i(t,\Delta ;\epsilon ),S_i^V(t,\Delta ;\epsilon )\} \quad \text {and} \quad i \ne 1. \qquad \end{aligned}$$

Moreover, note that (here suppressing the dependence on $\epsilon$)

B194 $$\begin{aligned} S^*_1(\Delta ;\Delta )&= \frac{S^*_1(0;\Delta )}{N_1(\epsilon )}(N_1(\epsilon ) - W^*_1(\Delta ;\Delta ))e^{\sum _{j=1}^n\left[ \frac{\beta ^1_{1j}}{\mu ^1_j}R^*_j(\Delta ;\Delta ) + \frac{\beta ^3_{1j}}{\mu ^2_j}R^{V\,*}_j(\Delta ;\Delta )\right] } \end{aligned}$$

B195 $$\begin{aligned}&= \sigma (\epsilon - (\epsilon - \epsilon ')-W_1(\Delta ;\epsilon '))e^{\sum _{j=1}^n\left[ \frac{\beta ^1_{1j}}{\mu ^1_j}R_j(0;\epsilon ) + \frac{\beta ^3_{1j}}{\mu ^2_j}R^V_j(0;\epsilon )\right] } + O(\Delta )\end{aligned}$$

B196 $$\begin{aligned}&= \sigma (\epsilon ' - W_1(0;\epsilon '))e^{\sum _{j=1}^n\left[ \frac{\beta ^1_{1j}}{\mu ^1_j}R_j(0;\epsilon ') + \frac{\beta ^3_{1j}}{\mu ^2_j}R^V_j(0;\epsilon ')\right] } + O(\Delta )\end{aligned}$$

B197 $$\begin{aligned}&= S_1(0;\epsilon ') + O(\Delta ) \end{aligned}$$

and hence,

B198 $$\begin{aligned} |S_1(\Delta ;\Delta ;\epsilon ) - S_1(0;\epsilon ')|< L'\Delta \end{aligned}$$

for some $L' > 0$. Now, as (again suppressing the dependence on $\epsilon$)

B199 $$\begin{aligned} S_1(\Delta ;\Delta ) + I_1(\Delta ;\Delta ) + R_1(\Delta ;\Delta ) + S_1^V(\Delta ;\Delta ) + I_1^V(\Delta ;\Delta ) + R_1^V(\Delta ;\Delta ) = \epsilon ,\nonumber \\ \end{aligned}$$

it is necessary that

B200 $$\begin{aligned} |S_1^V(\Delta ;\Delta ;\epsilon ) - (\epsilon - \epsilon ')|\le L''\Delta \end{aligned}$$

for some $L'' > 0$. Thus, in particular, the values of the model variables $f_i^*$ at time $\Delta$ converge to the initial conditions of the $\epsilon '$ case, except that

B201 $$\begin{aligned} \lim _{\Delta \rightarrow 0}(S^{V\,*}_1(0;\Delta )) > S^V_1(0;\epsilon '). \end{aligned}$$

Moreover, note that for any $t \ge 0$,

B202 $$\begin{aligned} W^*_i(\Delta +t,\Delta ;\epsilon ) - W_i^*(\Delta ;\Delta ;\epsilon ) = W_i(\Delta +t;\epsilon ') -W_1(\Delta ;\epsilon ') \end{aligned}$$

and so, as ${\varvec{U}}^*$ is bounded in $[\Delta ,\infty )$

B203 $$\begin{aligned} \bigg |\bigg (W^*_i(\Delta +t,\Delta ;\epsilon )-W_1^*(\Delta ;\Delta ;\epsilon )\bigg ) - W_i(t;\epsilon ') \bigg |< L''' \Delta \quad \forall t > 0 \end{aligned}$$

for some $L'''$. Thus, define variables with a hat to denote those from the disease trajectory with initial conditions given by

B204 $$\begin{aligned} {\hat{f}}_i(0;\epsilon ') := \lim _{\Delta \rightarrow 0}\bigg (f_i^*(\Delta ;\Delta ;\epsilon )\bigg ) \end{aligned}$$

and with vaccination policy given by $W_i(t;\epsilon ')$. Then, by considering the starred variables to come from an epidemic started at time $t=\Delta$, Lemma 14 shows that

B205 $$\begin{aligned} \lim _{\Delta \rightarrow 0}(f^*_i(t;\Delta ;\epsilon )) = {\hat{f}}_i(t;\epsilon '). \end{aligned}$$

Thus, one can take the $\Delta \rightarrow 0$ limit in (B188) and (B189) to show

B206 $$\begin{aligned} {\hat{I}}_i(t;\epsilon ') + {\hat{R}}_i(t;\epsilon ') + {\hat{I}}^V_i(t;\epsilon ') + {\hat{R}}^V_i(t;\epsilon ') \le I_i(t;\epsilon ) + R_i(t;\epsilon ) + I^V_i(t;\epsilon ) + R^V_i(t;\epsilon )\nonumber \\ \end{aligned}$$

and

B207 $$\begin{aligned} R_i(t;\epsilon ') \le R_i(t;\epsilon ) . \end{aligned}$$

Taking $t \rightarrow \infty$ in these inequalities shows that

B208 $$\begin{aligned} {\hat{R}}_i(\infty ;\epsilon ') + {\hat{R}}_i(\infty ;\epsilon ') \le R_i(\infty ;\epsilon ) +R^V_i(\infty ;\epsilon ) \quad \text {and} \quad {\hat{R}}_i(\infty ;\epsilon ')\le R_i(\infty ;\epsilon )\qquad \end{aligned}$$

and hence, for any $\kappa _i \in [0,1]$,

B209 $$\begin{aligned} {\hat{R}}_i(\infty ;\epsilon ') + \kappa _i {\hat{R}}_i(\infty ;\epsilon ')&= (1-\kappa _i) {\hat{R}}_i(\infty ;\epsilon ') + \kappa _i( {\hat{R}}_i(\infty ;\epsilon ') + {\hat{R}}_i(\infty ;\epsilon ')) \end{aligned}$$

B210 $$\begin{aligned}&\le (1-\kappa _i) R_i(\infty ;\epsilon ) + \kappa _i( R_i(\infty ;\epsilon ) +R^V_i (\infty ;\epsilon ))\end{aligned}$$

B211 $$\begin{aligned}&= R_i(\infty ;\epsilon ) + \kappa _i R^V_i (\infty ;\epsilon ). \end{aligned}$$

Summing these inequalities over *i* gives

B212 $$\begin{aligned} {\hat{H}}(\hat{{\varvec{W}}}(t;\epsilon ');\epsilon ') \le H({\varvec{W}}(t;\epsilon );\epsilon ). \end{aligned}$$

Finally, note that by Lemma 16, as the only change between cases ${\hat{H}}$ and *H* is an increase in one of the values of $S^V$,

B213 $$\begin{aligned} H({\varvec{W}}(t;\epsilon ');\epsilon ')\le {\hat{H}}({\varvec{W}}(t;\epsilon ');\epsilon ') \end{aligned}$$

which, combined with (B212), completes the proof of this proposition.

### Theorem 2

This allows the overall proof of Theorem 2. The proof will rely on Theorem 1, which allows the creation of an *O*(1) decrease in the objective function by reducing $\epsilon$. By comparing a sequence of policies satisfying (A2) with a sequence that does not satisfy (A2) and using Proposition 6, one can then create a sequence of optimal policies such that the associated sequence of objective values decreases by at least a fixed quantity at each step (and thus will eventually become negative, giving a contradiction).

Suppose (for a contradiction) that Theorem 2 does not hold for some fixed $\alpha$, $\tau$ and *w*. Thus, for any $\eta > 0$, there is an $\epsilon \in (0,\eta )$ such that, for some ${\varvec{U}}$ satisfying (A2),

B214 $$\begin{aligned} H({\varvec{U}}(t;\epsilon );\epsilon ) \le H(\overline{{\varvec{U}}}(t;\epsilon );\epsilon ). \end{aligned}$$

By optimality of $\overline{{\varvec{U}}}(t;\epsilon )$, (B214) must in fact be an equality, and so it can be assumed that ${\varvec{U}}(t;\epsilon )$ = $\overline{{\varvec{U}}}(t;\epsilon )$, which will be done in the remainder of this proof (that is, if for some $\epsilon$ there is an optimal solution satisfying (A2), then it will be assumed that $\overline{{\varvec{U}}}$ satisfies (A2)). Thus, there is some $\epsilon _0$ such that

B215 $$\begin{aligned} H(\overline{{\varvec{U}}}(t;\epsilon _0);\epsilon _0) \le H(\tilde{\overline{{\varvec{U}}}}(t;\epsilon _0);\epsilon _0) \end{aligned}$$

where $\tilde{\overline{{\varvec{U}}}}$ is defined by (A4). Now, for $\epsilon < \epsilon _0$, define ${\varvec{U}}^0(t;\epsilon )$ by

B216 $$\begin{aligned} U^0_1(t;\epsilon ) = \left\{ \begin{matrix} {\overline{U}}_1(t;\epsilon _0) &{} \text {if } W_1(t;\epsilon _0) < \epsilon \\ 0 &{} \text {otherwise} \end{matrix}\right. \quad \text {and} \quad {\overline{U}}_i(t;\epsilon ) = U^0_i(t;\epsilon _0) \quad \forall i \ne 1 \qquad \end{aligned}$$

and note that this means that

B217 $$\begin{aligned} {\varvec{U}}^0(t;\epsilon _0) = \overline{{\varvec{U}}}(t;\epsilon _0) \quad \forall t \ge 0. \end{aligned}$$

By (A163) in the proof of Theorem 1, there exists some $\delta _1 > 0$ such that, for all $\epsilon < \delta _1$,

B218 $$\begin{aligned} H({\varvec{U}}^0(t;\epsilon );\epsilon ) >&H(\tilde{{\varvec{U}}}^0(t;\epsilon );\epsilon )\\&+ \frac{1}{2}\int _{z_0}^{\tau } (1-\alpha )P(t)\bigg [(1-{\mathcal {Y}}^*)\kappa _1(X(t) - Y(t)) + (1-\kappa _1) X(t)\bigg ]{\textrm{d}}t. \nonumber \end{aligned}$$

where

B219 $$\begin{aligned}{} & {} X(t) = \sum _{j=1}^n \beta _{1j}^1I_j(t;\epsilon ) + \beta _{1j}^3I_j^V(t;\epsilon ), \end{aligned}$$

B220 $$\begin{aligned}{} & {} Y(t) = \sum _{j=1}^n \beta _{1j}^2I_j(t;\epsilon ) + \beta _{1j}^4I_j^V(t;\epsilon ) \end{aligned}$$

and

B221 $$\begin{aligned} P(t) = \exp \bigg [-\sum _{j=1}^n \bigg (\frac{\beta _{1j}^1R_j(t;0)}{\mu _j^1} + \frac{\beta _{1j}^2R_j^V(t;0)}{\mu _j^2}\bigg )\bigg ]. \end{aligned}$$

Note that $\rho _0$, $\tau$ and ${\mathcal {Y}}^*$ are independent of ${\varvec{U}}^0$, but *X*(*t*), *Y*(*t*) and *P*(*t*) are not. However, note that

B222 $$\begin{aligned} \frac{{\textrm{d}}I_i(t;\epsilon )}{{\textrm{d}}t} \ge - \mu _i^1I_i(t;\epsilon ) \end{aligned}$$

and so

B223 $$\begin{aligned} X(t)-Y(t) \ge \sum _{j=2}^n (\beta _{1j}^1 - \beta _{1j}^3) e^{-\mu _j^1 t}I_j(0;\epsilon ) > 0, \end{aligned}$$

by assumption (B170), giving a bound that is independent of ${\varvec{U}}^0$. Moreover,

B224 $$\begin{aligned} X(t) \ge X(t) - Y(t) > 0. \end{aligned}$$

Finally, for $$\epsilon \le 1$$,

B225 $$\begin{aligned}{} & {} P(t) \ge \exp \bigg [-\sum _{j=1}^n \bigg (\frac{\beta _{1j}^1N_j(\epsilon )}{\mu _j^1} + \frac{\beta _{1j}^2N_j(\epsilon )}{\mu _j^2}\bigg )\bigg ]\nonumber \\{} & {} \quad \ge \exp \bigg [-\sum _{j=1}^n \bigg (\frac{\beta _{1j}^1N_j(1)}{\mu _j^1} + \frac{\beta _{1j}^2N_j(1)}{\mu _j^2}\bigg )\bigg ] > 0 \end{aligned}$$

and this bound is again independent of ${\varvec{U}}^0$. Thus,

B226 $$\begin{aligned} H({\varvec{U}}^0(t;\epsilon );\epsilon ) > H(\tilde{{\varvec{U}}}^0(t;\epsilon );\epsilon ) + K \quad \forall \epsilon < \delta _1 \end{aligned}$$

for some constant $K > 0$ where this is now independent of ${\varvec{U}}^0$. Now, by assumption, there must exist some $\epsilon _1 \in (0,\delta _1)$ such that $\overline{{\varvec{U}}}(t;\epsilon _1)$ meets the conditions (A2) so

B227 $$\begin{aligned} H(\overline{{\varvec{U}}}(t;\epsilon _1);\epsilon _1) \le H(\tilde{\overline{{\varvec{U}}}}(t;\epsilon _1);\epsilon _1) \end{aligned}$$

while by optimality

B228 $$\begin{aligned} H(\overline{{\varvec{U}}}(t;\epsilon _1);\epsilon _1) \le H(\tilde{{\varvec{U}}}^0(t;\epsilon _1);\epsilon _1) < H({\varvec{U}}^0(t;\epsilon _1);\epsilon _1) - K. \end{aligned}$$

Now, moreover, note that by Proposition 6,

B229 $$\begin{aligned} H({\varvec{U}}^0(t;\epsilon _1);\epsilon _1) \le H({\varvec{U}}^0(t;\epsilon _0);\epsilon _0) = H(\overline{{\varvec{U}}}(t;\epsilon ^0);\epsilon ^0) \end{aligned}$$

and so

B230 $$\begin{aligned} H(\overline{{\varvec{U}}}(t;\epsilon _1);\epsilon _1) \le H(\overline{{\varvec{U}}}(t;\epsilon _0);\epsilon _0) - K. \end{aligned}$$

Now, this can be continued iteratively so that, for any $n \ge 0$,

B231 $$\begin{aligned} H(\overline{{\varvec{U}}}(t;\epsilon _n);\epsilon _n) \le H(\overline{{\varvec{U}}}(t;\epsilon _0);\epsilon _0) - Kn \end{aligned}$$

However, this means that eventually, one finds

B232 $$\begin{aligned} H(\overline{{\varvec{U}}}(t;\epsilon _n);\epsilon _n) < 0 \end{aligned}$$

which is a contradiction. Thus, for each fixed $\alpha$, *w* and $\tau$, there must exist some $\eta$ such that for any $\epsilon \in (0,\eta )$, the optimal solution does not satisfy (A2).

Now, suppose that $\int _0^t \chi (s){\textrm{d}}s > 0$ and suppose $\overline{{\varvec{U}}}(t;\epsilon )$ is an optimal solution for each value of $\epsilon$ such that, for each *t*

B233 $$\begin{aligned} \sum _{i=1}^n {\overline{W}}_i(t;\epsilon ) = \min \bigg (\int _0^t \chi (s){\textrm{d}}s, 1\bigg ) \end{aligned}$$

(note that this can be assumed by Theorem 2 in Penn and Donnelly (2022)). Now, suppose that, for some *t*

B234 $$\begin{aligned} \lim _{\epsilon \rightarrow 0}\bigg (\frac{{\overline{W}}_1(t;\epsilon )}{\epsilon } \bigg )\ne 1 \quad \text {and} \quad \min \bigg (\int _0^t \chi (s){\textrm{d}}s, 1\bigg ) > 0. \end{aligned}$$

This means that there exists some $\delta > 0$ such that there is a subsequence $\epsilon _m$ satisfying

B235 $$\begin{aligned} \frac{ {\overline{W}}_1(t;\epsilon _m)}{\epsilon _m}< 1-\delta < 1 \quad \text {and}\quad \lim _{m \rightarrow \infty }(\epsilon _m) = 0 \end{aligned}$$

noting that

B236 $$\begin{aligned} \frac{ {\overline{W}}_1(t;\epsilon _m)}{\epsilon _m} \le 1 \quad \forall \epsilon _m > 0. \end{aligned}$$

However, this means that for each *m*, $\overline{{\varvec{U}}}(t;\epsilon _m)$ satisfies the condition (A2) with $\tau = t$, $\alpha = 1-\delta$ and $w = \min \bigg (\int _0^t \chi (s){\textrm{d}}s, 1\bigg )$. This is a contradiction to the previous part of the proof (as $\lim _{m\rightarrow \infty }(\epsilon _m) = 0$) and hence

B237 $$\begin{aligned} \lim _{\epsilon \rightarrow 0}\bigg (\frac{W_1^*(t;\epsilon )}{\epsilon }\bigg ) = 1 \quad \forall t \quad \text {s.t.} \quad \min \bigg (\int _0^t \chi (s){\textrm{d}}s, 1\bigg ) > 0, \end{aligned}$$

as required.

## Appendix C Proof of Theorem 3

Recall the definitions from the main text.

C239 $$\begin{aligned}{} & {} \beta '_{ij} = \left\{ \begin{matrix} \beta ^1_{ij} &{} \text {if } i,j\le n\\ \beta ^2_{i(n-j)} &{} \text {if } i \le n< j \le 2n\\ \beta ^3_{(n-i)j} &{} \text {if } j \le n< i\le 2n \\ \beta ^4_{(n-i)(n-j)} &{} \text {if } n < i,j \le 2n\\ \end{matrix}\right. , \end{aligned}$$

C240 $$\begin{aligned}{} & {} \mu '_i = \left\{ \begin{matrix} \mu _i^1 &{} \text {if } i \le n\\ \mu _{(i-n)}^2 &{} \text {if } n < i \le 2n \end{matrix}\right. , \end{aligned}$$

C241 $$\begin{aligned}{} & {} p'_i = \left\{ \begin{matrix} p_i &{} \text {if } i \le n\\ \kappa _{(i-n)} p_{(i-n)} &{} \text {if } n<i\le 2n\end{matrix}\right. , \end{aligned}$$

C242 $$\begin{aligned}{} & {} Q_{ij} = \frac{1}{1-e^{-\sum _{j=1}^{2n}\frac{\beta '_{ij}}{\mu '_j}R_j(\infty ;0)}}\bigg [\delta _{ij} +\frac{ S_i(0;0)e^{-\sum _{j=1}^{2n}\frac{\beta '_{ij}}{\mu '_j}R_j(\infty ;0)}\beta '_{ij}}{\mu '_j}\bigg ], \qquad \end{aligned}$$

and

C243 $$\begin{aligned} {\varvec{x}} = {\varvec{Q}}^{-T}{\varvec{p}}' \quad \text {and} \quad y_i = \frac{S_i(0;0)}{N_i}(x_{i+n} - x_i) \quad \forall i \in \{1,\ldots ,n\} . \end{aligned}$$

### Theorem 3

Suppose that, for all $\epsilon > 0$

C244 $$\begin{aligned} B(t;\epsilon ) = \epsilon \quad \forall t \ge 0 \end{aligned}$$

and that all other parameter values and initial conditions are independent of $\epsilon$. Suppose that *A*(*t*) is a continuous function with

C245 $$\begin{aligned} A(0) > 0 \end{aligned}$$

and that the matrix *M* is invertible. Assuming that $\epsilon$ is sufficiently small so that it exists, define

C246 $$\begin{aligned} \tau (\epsilon ) := \inf \bigg \{t : \int _0^t A(s){\textrm{d}}s = \epsilon \bigg \}. \end{aligned}$$

Suppose that ${\varvec{U}}$ satisfies the condition

C247 $$\begin{aligned} \sum _{i=1}^nU_i(s) = \min \bigg (\int _0^t\chi (s){\textrm{d}}s,1\bigg ) \end{aligned}$$

where $\chi$ is defined in (B169). Then, for sufficiently small $\epsilon$, the objective function is given by

C248 $$\begin{aligned} H({\varvec{U}}(t;\epsilon )) = H({\varvec{0}}) + {\varvec{y}}^T{\varvec{W}}(\tau (\epsilon );\epsilon ) + o(\epsilon ). \end{aligned}$$

Moreover, if there is a unique element of ${\varvec{y}}$ equal to the minimum of ${\varvec{y}}$ then the optimal vaccination policy (to leading order in $\epsilon$) is uniquely given by

C249 $$\begin{aligned} U_i(t;\epsilon ) = \left\{ \begin{matrix} A(t) &{}\text {if } i = \min \{ y_i : i \in \{1,\ldots ,n\} \} &{} \text {and } \int _0^t A(s){\textrm{d}}s < \epsilon \\ 0 &{} \text {otherwise} \\ \end{matrix}\right. . \end{aligned}$$

### Proposition 7

Note that the *n*-group model can be considered as a 2*n*-group model once vaccination has finished—an idea that is formalised in the below proposition. This moderately extends the previous work by incorporating the initial vaccination policy into the final size equation, but is not a major advancement on well-known results found in books such as Anderson and May (1992).

#### Proposition 7

Define for $$i \in \{1,\ldots ,n\}$$,

C250 $$\begin{aligned} (S_{n+i},I_{n+i},R_{n+i}) := (S^V_i, I^V_i, R^V_i). \end{aligned}$$

Define further

C251 $$\begin{aligned} \sigma _{i}(\epsilon ) = \left\{ \begin{matrix} -\frac{S_i(0;0)W_i(\tau (\epsilon ))}{N_i} &{} \text {if } i \le n\\ \frac{S_{i-n}(0;0)W_{i-n}(\tau (\epsilon ))}{N_{i-n}} &{} \text {if } n < i \le 2n\\ \end{matrix} \right. \end{aligned}$$

and

C252 $$\begin{aligned} \rho _i(\epsilon ) := R_i(\infty ;\epsilon ) - R_i(\infty ;0) \quad \forall i \in \{1,\ldots ,2n\}. \end{aligned}$$

Then, $\rho _i(\epsilon )$ is *o*(1) as $\epsilon \rightarrow 0$ and

C253 $$\begin{aligned} \sigma _i= \frac{\rho _i + S_i(0;0)e^{-\sum _{j=1}^{2n}\frac{\beta '_{ij}}{\mu '_j}R_j(\infty ;0)} \bigg (\sum _{j=1}^{2n}\frac{\beta '_{ij}}{\mu '_j}\rho _j \bigg )+ o(\sigma _i) + \sum _{j=1}^{2n}o(\rho _j) + O(\epsilon ^2)}{1-e^{-\sum _{j=1}^{2n}\frac{\beta '_{ij}}{\mu '_j}R_j(\infty ;0)}}.\nonumber \\ \end{aligned}$$

#### Proof

As *A* is continuous, there is some region $(0,\delta )$ such that

C254 $$\begin{aligned} \frac{A(0)}{2}<A(t) < 2A(0) \end{aligned}$$

and hence

C255 $$\begin{aligned} \int _0^{\delta }A(t){\textrm{d}}t > \frac{\delta A(0)}{2}. \end{aligned}$$

This lower bound is independent of $\epsilon$, and hence, for sufficiently small $\epsilon$,

C256 $$\begin{aligned} \int _0^{\delta }A(t){\textrm{d}}t > \epsilon . \end{aligned}$$

Now, by assumption,

C257 $$\begin{aligned} \sum _{i=1}^n U_i(t;\epsilon ) = \left\{ \begin{matrix} A(t) &{} \text {if } \int _0^tA(s){\textrm{d}}s < \epsilon \\ 0 &{}\text {otherwise}\\ \end{matrix} \right. . \end{aligned}$$

By continuity and the definition of $\tau (\epsilon )$,

C258 $$\begin{aligned} \int _0^{\tau (\epsilon )}A(t){\textrm{d}}t = \epsilon \end{aligned}$$

and note that it is necessary that $\tau (\epsilon ) = O(\epsilon )$ as

C259 $$\begin{aligned} \tau (\epsilon ) \le \frac{2\epsilon }{A(0)} \end{aligned}$$

for sufficiently small $\epsilon$.

Now, all of the variables are bounded independently of $\epsilon$ in the interval $[0,\tau (\epsilon )]$ (including ${\varvec{U}}$, which is bounded by 2*A*(0)). Moreover, assuming $N_i > 0$ for each $i \in \{1,\ldots ,n\}$,

C260 $$\begin{aligned} N_i - W_i> N_i - \epsilon > \frac{\min _i(N_i)}{2} \end{aligned}$$

for sufficiently small $\epsilon$. Thus, in particular, all of the derivatives of the model variables are bounded and so

C261 $$\begin{aligned} S_i(\tau (\epsilon );\epsilon ) = S_i(0;0) + O(\epsilon ) \end{aligned}$$

with analogous results for the other model variables, noting that the initial conditions are identical in each case. Thus, in particular,

C262 $$\begin{aligned} \frac{{\textrm{d}}S_i}{{\textrm{d}}t}(t;\epsilon ) = \frac{{\textrm{d}}S_i}{{\textrm{d}}t}(0;\epsilon ) -\frac{S_i(0;0) (U_i(t;\epsilon )-U_i(0;\epsilon ))}{N_i-W_i(0;\epsilon )} + O(\epsilon ) \quad \forall t \in (0,\epsilon ) ,\nonumber \\ \end{aligned}$$

noting that the $U_i(t;\epsilon )$ are the only quantities that can change by an *O*(1) amount in $O(\epsilon )$ time. Now, one can set $U_i(0;\epsilon ) = 0$ to reduce notation (noting that the model depends only on the integral of $U_i$). Moreover, as $W_i(0;\epsilon ) = 0$, the initial conditions are independent of $\epsilon$ and $\tau (\epsilon ) = O(\epsilon )$, integrating gives

C263 $$\begin{aligned} S_i(\tau (\epsilon );\epsilon ) = S_i(0;0) + \tau (\epsilon )\frac{{\textrm{d}}S_i}{{\textrm{d}}t}(0;0) - \frac{S_i(0;\epsilon )W_i(\tau (\epsilon );\epsilon )}{N_i} + O(\epsilon ^2).\qquad \end{aligned}$$

Similarly,

C264 $$\begin{aligned} S^V_i(\tau (\epsilon );\epsilon )) = S^V_i(0;0) + \tau (\epsilon )\frac{{\textrm{d}}S^V_i}{{\textrm{d}}t}(0;0) +\frac{S_i(0;0)W_i(\tau (\epsilon );\epsilon )}{N_i} + O(\epsilon ^2)\qquad \end{aligned}$$

while for the other model variables, $f_i$, there is no *O*(1) change to the derivative so

C265 $$\begin{aligned} f_i(\tau (\epsilon );\epsilon )) = f(0;0) + \tau (\epsilon )\frac{{\textrm{d}}f_i}{{\textrm{d}}t}(0;0) + O(\epsilon ^2). \end{aligned}$$

Now, for times $t \ge \tau (\epsilon )$, one has $U_i(t;\epsilon ) = 0$ and so a standard multi-group SIR model (with initial conditions given by the model variables evaluated at time $\tau (\epsilon )$) is recovered. Thus in particular, the final number infected can be formulated in terms of a final size equation, following the work of Anderson and May (1992) among others. Define, for $i \in \{1,\ldots ,n\}$,

C266 $$\begin{aligned} (S_{n+i},I_{n+i},R_{n+i}) = (S^V_i,I^V_i,R^V_i). \end{aligned}$$

This new 2*n* group model has the same behaviour as the original model if the parameters are

C267 $$\begin{aligned} \beta '_{ij} = \left\{ \begin{matrix} \beta ^1_{ij} &{} \text {if } i,j\le n\\ \beta ^2_{i(n-j)} &{} \text {if } i \le n< j \\ \beta ^3_{(n-i)j} &{} \text {if } j \le n< i \\ \beta ^4_{(n-i)(n-j)} &{} \text {if } n < i,j \\ \end{matrix}\right. , \quad \mu '_i = \left\{ \begin{matrix} \mu _i^1 &{} \text {if } i \le n\\ \mu _{(i-n)}^2 &{} \text {if } i > n\end{matrix}\right. \end{aligned}$$

and

C268 $$\begin{aligned} p'_i = \left\{ \begin{matrix} p_i &{} \text {if } i \le n\\ \kappa _{(i-n)} p_{(i-n)} &{} \text {if } i > n\end{matrix}\right. . \end{aligned}$$

Thus, integrating the $S_i$ equation between $\tau (\epsilon )$ and $t + \tau (\epsilon )$ gives:

C269 $$\begin{aligned}&\frac{{\textrm{d}}}{{\textrm{d}}t}\left( \log (S_i)\right) = -\sum _{j=1}^{2n}\frac{\beta '_{ij}}{\mu '_j} \frac{{\textrm{d}}R_j}{{\textrm{d}}t} \end{aligned}$$

C270 $$\begin{aligned}&\quad \Rightarrow \ln (S_i(t+\tau (\epsilon );\epsilon )) = \ln (S_i(\tau (\epsilon );\epsilon )) - \sum _{j=1}^{2n}\frac{\beta '_{ij}}{\mu '_j} \bigg [R_j(t+\tau (\epsilon );\epsilon ) - R_j(\tau (\epsilon );\epsilon )\bigg ]\end{aligned}$$

C271 $$\begin{aligned}&\quad \Rightarrow S_i(t+\tau (\epsilon );\epsilon ) = S_i(\tau (\epsilon );\epsilon )e^{-\sum _{j=1}^{2n}\frac{\beta '_{ij}}{\mu '_j} \left[ R_j(t+\tau (\epsilon );\epsilon ) - \tau (\epsilon )\frac{{\textrm{d}}R_j}{{\textrm{d}}t}(0;0)\right] } + O(\epsilon ^2) \end{aligned}$$

as $R_j(0;0) = 0$ for each *j*. Now, note that for any $t \ge 0$,

C272 $$\begin{aligned}{} & {} S_i(\tau (\epsilon );\epsilon ) + I_i(\tau (\epsilon );\epsilon )+R_i(\tau (\epsilon );\epsilon )\nonumber \\ {}{} & {} \quad = S_i(t+\tau (\epsilon );\epsilon ) + I_i(t+\tau (\epsilon );\epsilon )+R_i(t+\tau (\epsilon );\epsilon ) \end{aligned}$$

and hence, taking $t \rightarrow \infty$ and using Lemma 11 shows that

C273 $$\begin{aligned} S_i(\tau (\epsilon );\epsilon ) + I_i(\tau (\epsilon );\epsilon )+R_i(\tau (\epsilon );\epsilon ) = S_i(\infty ;\epsilon ) + R_i(\infty ;\epsilon ) . \end{aligned}$$

Hence, by (C265),

C274 $$\begin{aligned} S_i(\tau (\epsilon );\epsilon ) + I_i(0;0) + \tau (\epsilon )\left[ \frac{{\textrm{d}}I_i}{{\textrm{d}}t}(0;0) + \frac{{\textrm{d}}R_i}{{\textrm{d}}t}(0;0)\right] = S_i(\infty ;\epsilon ) + R_i(\infty ;\epsilon ) + O(\epsilon ^2).\nonumber \\ \end{aligned}$$

Now, substituting this into the limit of (C269) as $t \rightarrow \infty$ shows that

C275 $$\begin{aligned} R_i(\infty ;\epsilon ) =&S_i(\tau (\epsilon );\epsilon ) + I_i(0;0) + \tau (\epsilon )\left[ \frac{{\textrm{d}}I_i}{{\textrm{d}}t}(0;0) + \frac{{\textrm{d}}R_i}{{\textrm{d}}t}(0;\epsilon )\right] \nonumber \\&- S_i(\tau (\epsilon );\epsilon )e^{-\sum _{j=1}^{2n}\frac{\beta '_{ij}}{\mu '_j} \left[ R_j(\infty ;\epsilon ) - \tau (\epsilon )\frac{{\textrm{d}}R_j}{{\textrm{d}}t}(0;0)\right] } + O(\epsilon ^2). \end{aligned}$$

By treating this model as a model that has initial conditions given by the variable values at time $\tau (\epsilon )$, one sees that these initial conditions differ from the initial conditions of the $\epsilon = 0$ model by $O(\epsilon )$ (where no vaccination occurs in either case). This means that Proposition 5 can be used (as the vaccination policies ${\varvec{U}}$ must have uniformly bounded finite support for sufficiently small $\epsilon$) and so there exists some function $\delta (\epsilon )$ such that, for all sufficiently small $\epsilon$,

C276 $$\begin{aligned} |R_j(\infty ;\epsilon ) - R_j(\infty ;0)|< \delta (\epsilon ) \quad \forall j \quad \text {and} \quad \delta (\epsilon ) = o(1). \end{aligned}$$

Thus, in particular, one can define functions $\rho _j(\epsilon )$ such that

C277 $$\begin{aligned} R_j(\infty ;\epsilon ) = R_j(\infty ;0) + \rho _j(\epsilon ) \quad \forall j \in \{1,..,2n\} \end{aligned}$$

and

C278 $$\begin{aligned} \rho _j(\epsilon ) = o(1) \quad \text {as } \epsilon \rightarrow 0. \end{aligned}$$

Furthermore, defining $\sigma _i$ such that

C279 $$\begin{aligned} \sigma _{i}(\epsilon ) = \left\{ \begin{matrix} -\frac{S_i(0;0)W_i(\tau (\epsilon ))}{N_i} &{} \text {if } i \le n\\ \frac{S_{i-n}(0;0)W_{i-n}(\tau (\epsilon ))}{N_{i-n}} &{} \text {if } n < i \le 2n\\ \end{matrix} \right. \end{aligned}$$

gives

C280 $$\begin{aligned} S_i(\tau (\epsilon );\epsilon ) = S_i(0;0) + \tau (\epsilon )\frac{{\textrm{d}}S_i}{{\textrm{d}}t}(0;0) + \sigma _i(\epsilon ) +O(\epsilon ^2) \quad \forall i \in \{1,..,2n\}.\qquad \quad \end{aligned}$$

Now, when $\sigma _i(\epsilon ) = 0$ for all *i*, it must be the case that $\rho _i(\epsilon ) = 0$ for all *i* as the final size is unchanged (as no vaccination has taken place). Thus, in this case, (C275) can be linearised to give

C281 $$\begin{aligned}&O(\epsilon ^2) = \tau (\epsilon )\bigg [\frac{{\textrm{d}}S_i}{{\textrm{d}}t}(0;0) + \frac{{\textrm{d}}I_i}{{\textrm{d}}t}(0;0) \nonumber \\&\qquad \qquad + \frac{{\textrm{d}}R_i}{{\textrm{d}}t}(0;0) e^{-\sum _{j=1}^{2n}\frac{\beta '_{ij}}{\mu '_j}R_j(\infty ;0)} \left( -\frac{{\textrm{d}}S_i}{{\textrm{d}}t}(0;0)+S_i(0;0)\sum _{j=1}^{2n}\frac{\beta '_{ij}}{\mu '_j}\frac{{\textrm{d}}R_j}{{\textrm{d}}t}(0;0)\right) \bigg ]. \end{aligned}$$

Note that this equality does indeed hold, as in the no vaccination case

C282 $$\begin{aligned} \frac{{\textrm{d}}S_i}{{\textrm{d}}t}(0;0) + \frac{{\textrm{d}}I_i}{{\textrm{d}}t}(0;0) + \frac{{\textrm{d}}R_i}{{\textrm{d}}t}(0;0) = 0 \end{aligned}$$

is the conservation of population law, while

C283 $$\begin{aligned} -\frac{{\textrm{d}}S_i}{{\textrm{d}}t}(0;0)+S_i(0;0)\sum _{j=1}^{2n}\frac{\beta '_{ij}}{\mu '_{j}}\frac{{\textrm{d}}R_j}{{\textrm{d}}t}(0;0) = -\frac{{\textrm{d}}S_{i}}{{\textrm{d}}t}(0;0)+S_i(0;0)\sum _{j=1}^{2n}\beta \prime _{ij}\prime I_{j}(0;0) = 0. \end{aligned}$$

This means that, for nonzero $\sigma _i$, all terms not dependent on $\sigma _i$ or $\rho _i$ cancel and so the linearisation becomes

C284 $$\begin{aligned} \rho _i&= \sigma _i - \sigma _i e^{-\sum _{j=1}^{2n}\frac{\beta '_{ij}}{\mu '_j}R_j(\infty ;0)} - S_i(0;0)e^{-\sum _{j=1}^{2n}\frac{\beta '_{ij}}{\mu '_j}R_j(\infty ;0)} \sum _{j=1}^{2n}\frac{\beta '_{ij}}{\mu '_j}\rho _j\nonumber \\&+ o(\sigma _i) + \sum _{j=1}^{2n}o(\rho _j) + O(\epsilon ^2) \end{aligned}$$

and so

C285 $$\begin{aligned} \sigma _i= \frac{\rho _i + S_i(0;0)e^{-\sum _{j=1}^{2n}\frac{\beta '_{ij}}{\mu '_j}R_j(\infty ;0)} \bigg (\sum _{j=1}^{2n}\frac{\beta '_{ij}}{\mu '_j}\rho _j \bigg )+ o(\sigma _i) + \sum _{j=1}^{2n}o(\rho _j) + O(\epsilon ^2)}{1-e^{-\sum _{j=1}^{2n}\frac{\beta '_{ij}}{\mu '_j}R_j(\infty ;0)}}.\nonumber \\ \end{aligned}$$

as required.

### Proposition 8

The result of Proposition 7 can be written as a system of equations for vectors $\varvec{\sigma }$ and $\varvec{\rho }$

C286 $$\begin{aligned} \varvec{\sigma } = {\varvec{Q}}\varvec{\rho } + o(\varvec{\sigma }) + \sum _{j=1}^{2n}o(\rho _j) + O(\epsilon ^2) \end{aligned}$$

for some matrix ${\varvec{Q}}$ with nonzero determinant by assumption. However, it is important to establish the dominant balance in these equations, which is done through the following proposition, another result that the authors believe is novel to the literature.

#### Proposition 8

C287 $$\begin{aligned} \rho _i(\epsilon ) = O(\epsilon ) \quad \forall i \in \{1,\ldots ,2n\}. \end{aligned}$$

#### Proof

Suppose that this does not hold. Thus, there must be some sequence $\epsilon _m$ such that, for some *i*

C288 $$\begin{aligned} \lim _{m \rightarrow \infty }\bigg (\frac{\rho _i(\epsilon _m)}{\epsilon _m}\bigg ) = \infty \quad \text {and} \quad \lim _{m \rightarrow \infty }(\epsilon _m) = 0. \end{aligned}$$

Define $J^*(\epsilon )$ such that

C289 $$\begin{aligned} J^*(\epsilon ) = \text {argmax}\left\{ |\rho _j(\epsilon )|: j \in \{1,\ldots ,2n\}\right\} . \end{aligned}$$

Now, by the finiteness of $\{1,\ldots ,2n\}$, there exists some subsequence $\epsilon _{m_k}$ and some fixed $J \in \{1,\ldots ,2n\}$ such that

C290 $$\begin{aligned} \quad J^*(\epsilon _{m_k}) = J \quad \forall k. \end{aligned}$$

For notational convenience, assume that the original sequence $\epsilon _m$ has this property. Note that

C291 $$\begin{aligned} \lim _{m \rightarrow \infty }\bigg ( \frac{\sigma _j(\epsilon _m)}{\rho _J(\epsilon _m)}\bigg ) = \lim _{m \rightarrow \infty }\bigg ( \frac{\sigma _j(\epsilon _m)}{\epsilon }\times \frac{\epsilon }{\rho _J(\epsilon _m)}\bigg ) = 0. \end{aligned}$$

as $\sigma _j(\epsilon ) = O(\epsilon )$ and $\epsilon = o(\rho _i(\epsilon )) \le o(\rho _J(\epsilon ))$. Moreover,

C292 $$\begin{aligned}{} & {} \lim _{m \rightarrow \infty }\bigg ( \frac{O(\epsilon _m^2)}{\rho _J(\epsilon _m)}\bigg ) = \lim _{m \rightarrow \infty }\bigg (\epsilon _m \times \frac{O(\epsilon _m)}{\rho _J(\epsilon _m)}\bigg ) = 0 , \end{aligned}$$

C293 $$\begin{aligned}{} & {} \lim _{m \rightarrow \infty }\bigg ( \frac{o(\sigma _j(\epsilon _m)))}{\rho _J(\epsilon _m)}\bigg ) = \lim _{m \rightarrow \infty }\bigg (o(1) \times \frac{\sigma _j(\epsilon _m)}{\rho _J(\epsilon _m)}\bigg ) = 0 \end{aligned}$$

and

C294 $$\begin{aligned} \bigg |\lim _{m \rightarrow \infty }\bigg ( \frac{o(\rho _j(\epsilon _m))}{\rho _J(\epsilon _m)}\bigg )\bigg |\le \lim _{m \rightarrow \infty }\bigg (\bigg |\frac{o(\rho _j(\epsilon _m))}{\rho _j(\epsilon _m)}\bigg |\bigg ) = 0. \end{aligned}$$

Note that there is some abuse of notation in these calculations, but, for example, an $O(\epsilon ^2)$ term in the limit represents any function which is $O(\epsilon ^2)$. Thus, dividing (C286) by $\rho _J(\epsilon _m)$ and taking *m* to $\infty$ shows that

C295 $$\begin{aligned} \lim _{m \rightarrow \infty }\bigg (\frac{{\varvec{Q}}\varvec{\rho }}{\rho _J(\epsilon _m)}\bigg ) = {\varvec{0}}. \end{aligned}$$

Define

C296 $$\begin{aligned} \hat{\varvec{\rho }}(\epsilon ) := \frac{\varvec{\rho }(\epsilon )}{\sum _{j=1}^{2n}|\rho _j(\epsilon )|} \end{aligned}$$

and note that

C297 $$\begin{aligned} \bigg |\bigg (\frac{\sum _{j=1}^{2n}|\rho _j(\epsilon _m)|}{\rho _J(\epsilon _m)}\bigg )\bigg |\in [1, 2n] \end{aligned}$$

and thus remains finite and nonzero. Thus,

C298 $$\begin{aligned} {\varvec{0}}&= \lim _{m \rightarrow \infty }\bigg (\frac{{\varvec{Q}}\varvec{\rho }}{\rho _J(\epsilon _m)}\bigg ) \end{aligned}$$

C299 $$\begin{aligned}&= \lim _{m \rightarrow \infty }\bigg (\frac{\sum _{j=1}^{2n}|\rho _j(\epsilon _m)|}{\rho _J(\epsilon _m)}\times {\varvec{Q}}\hat{\varvec{\rho }}(\epsilon _m)\bigg ) , \end{aligned}$$

which means

C300 $$\begin{aligned} {\varvec{0}}&= \lim _{m \rightarrow \infty }\bigg ({\varvec{Q}}\hat{\varvec{\rho }}(\epsilon _m)\bigg ). \end{aligned}$$

However, note that

C301 $$\begin{aligned} \sum _{j=1}^{2n} |{\hat{\rho }}_i(\epsilon )|= 1 \end{aligned}$$

and hence the sequence $\hat{\varvec{\rho }}$ is bounded. Thus, by the Bolzano–Weierstrass theorem, there must be some subsequence $m_k$ such that $\lim _{k\rightarrow \infty }(\hat{\varvec{\rho }}(\epsilon _{m_k}))$ exists and is equal to some $\varvec{\rho }^*$ where

C302 $$\begin{aligned} \sum _{j=1}^{2n}|\rho ^*_j|= 1. \end{aligned}$$

However, then, by continuity and the fact that ${\varvec{Q}}$ is invertible,

C303 $$\begin{aligned} {\varvec{Q}}\varvec{\rho }^* = {\varvec{0}} \Rightarrow \varvec{\rho }^* = {\varvec{0}} \end{aligned}$$

which is a contradiction to (C302) as required. Thus, it must be the case that $\rho (\epsilon ) = O(\epsilon )$ $\square$

### Theorem 3

Combining Proposition 8 with the fact that $\sigma _i = O(\epsilon )$ means that (C286) can be written as

C304 $$\begin{aligned} \varvec{\sigma } = {\varvec{Q}}\varvec{\rho } + o(\epsilon ). \end{aligned}$$

Thus, one can multiply the equation by $M^{-1}$ to get

C305 $$\begin{aligned} \varvec{\rho } = {\varvec{Q}}^{-1}\varvec{\sigma } + o(\epsilon ). \end{aligned}$$

Hence, given vectors ${\varvec{p}}$ and ${\varvec{q}}$ where

C306 $$\begin{aligned} {\varvec{p}}_i := p_i \quad \text {and} \quad {\varvec{q}}_i = p_i\kappa _i \quad \forall i \in \{1,\ldots ,n\}, \end{aligned}$$

the change to the objective function is given by:

C307 $$\begin{aligned} ({\varvec{p}},{\varvec{q}})^T\varvec{\rho }&= ({\varvec{p}},{\varvec{q}})^T\bigg [ {\varvec{Q}}^{-1}\varvec{\sigma } + o(\epsilon )\bigg ] \end{aligned}$$

C308 $$\begin{aligned}&:= {\varvec{x}}^T\varvec{\sigma } + o(\epsilon ). \end{aligned}$$

Now, note that, for $i \in \{1,\ldots ,n\}$,

C309 $$\begin{aligned} \sigma _i = -\frac{S_i(0;0)W_i(\tau (\epsilon );\epsilon )}{N_i} \end{aligned}$$

while, for $$i \in \{n+1,\ldots ,2n\}$$

C310 $$\begin{aligned} \sigma _i = -\sigma _{i-n} . \end{aligned}$$

Hence, one can write (C308) as

C311 $$\begin{aligned} ({\varvec{p}}^T,{\varvec{q}}^T)\varvec{\rho } = {\varvec{y}}^T{\varvec{W}}(\tau (\epsilon );\epsilon ) + o(\epsilon ), \end{aligned}$$

where

C312 $$\begin{aligned} {\varvec{y}}&= \frac{S_i(0;0)}{N_i}\bigg [-(x_1,....x_n)^T + (x_{n+1},\ldots ,x_{2n})^T\bigg ], \end{aligned}$$

as required by Theorem 3. The only restriction is that all the $W_i$ are non-negative and that

C313 $$\begin{aligned} \sum _{i=1}^nW_i(\tau (\epsilon );\epsilon ) = \epsilon \end{aligned}$$

and so the optimisation problem becomes

C314 $$\begin{aligned} \min \{{\varvec{y}}^T{\varvec{w}} : {\varvec{w}} \ge {\varvec{0}} \quad \text {and} \quad \sum _{i=1}^nw_i = \epsilon \}. \end{aligned}$$

Now, by Theorem 17, proved in Penn and Donnelly (2022) and stated in the appendices, it must be the case that the objective function is non-increasing in ${\varvec{w}}$. Thus, in particular, one must have

C315 $$\begin{aligned} {\varvec{y}} \le {\varvec{0}} \end{aligned}$$

as otherwise, if $y_i > 0$ then setting ${\varvec{w}} = \epsilon {\varvec{e}}_i$ (where ${\varvec{e}}_i$ is the ith canonical basis vector) means that

C316 $$\begin{aligned} H({\varvec{U}}(t;\epsilon )) = H({\varvec{U}}(t;0)) + y_i\epsilon + o(\epsilon ) \end{aligned}$$

and so, for sufficiently small $\epsilon$,

C317 $$\begin{aligned} H({\varvec{U}}(t;\epsilon )) > H({\varvec{U}}(t;0)) \end{aligned}$$

which is a contradiction. Hence, ${\varvec{y}} \le {\varvec{0}}$ which means that the optimisation problem is an example of a continuous knapsack problem and one can readily see that a solution is given by

C318 $$\begin{aligned} w^*_i = \left\{ \begin{matrix} \epsilon &{}\text {if } i = \min \{ y_i\} \\ 0 &{} \text {otherwise} \\ \end{matrix}\right. . \end{aligned}$$

As this minimum is unique by assumption, this is the unique leading-order optimal solution to the optimisation problem.

A technical note is that this only proves the form of the optimal solution to leading order. Indeed, if

C319 $$\begin{aligned} w_i = w_i^* + o(\epsilon ), \end{aligned}$$

then the optimal objective value is unchanged to leading order. Hence, this restriction is given in the statement of the theorem (although in practice is unimportant).

## Appendix D Supplementary Lemmas

This section contains the supplementary lemmas that have been used in the proofs of Theorems 1–3. All but two of these lemmas were proved in Penn and Donnelly (2022) and so their proofs will not be reproduced here, but they have been included for completeness and for ease of access. The exceptions are Lemma 15 and 16.

### Lemma 9

#### Lemma 9

Consider a continuous, time-dependent, matrix *A*(*t*) which satisfies

D320 $$\begin{aligned} A(t)_{ij} \ge 0 \quad \forall t \ge 0 \quad \text {and} \quad \forall i \ne j \end{aligned}$$

and a constant matrix *B* that satisfies

D321 $$\begin{aligned} B_{ij} \ge 0 \quad \forall t \ge 0 \quad \text {and} \quad \forall i \ne j. \end{aligned}$$

Then, suppose that each element of *A*(*t*) is non-increasing with *t* and that

D322 $$\begin{aligned} A(t)_{ij} \ge B_{ij} \quad \forall t \ge 0 \quad \text {and} \quad \forall i \ne j. \end{aligned}$$

Moreover, define an initial condition ${\varvec{v}}$ and suppose that ${\varvec{y}}$ and ${\varvec{z}}$ solve the systems

D323 $$\begin{aligned} \frac{{\textrm{d}}{\varvec{y}}}{{\textrm{d}}t} = A(t) {\varvec{y}} \quad \text {and} \quad \frac{{\textrm{d}}{\varvec{z}}}{{\textrm{d}}t} = B {\varvec{z}} \end{aligned}$$

with

D324 $$\begin{aligned} {\varvec{y}}(0) = {\varvec{z}}(0) = {\varvec{v}} \ge {\varvec{0}}. \end{aligned}$$

Then,

D325 $$\begin{aligned} {\varvec{y}}(t) \ge {\varvec{z}}(t) \ge {\varvec{0}} \quad \forall t \ge 0. \end{aligned}$$

#### Proof

This was proved as Lemma B.2 in Penn and Donnelly (2022)

### Lemma 10

#### Lemma 10

Define the set of functions

D326 $$\begin{aligned} {\mathcal {F}}_i(t) := \bigg \{ S_i(t), I_i(t),R_i(t),S^V_i(t),I^V_i(t),R^V_i(t)\bigg \} . \end{aligned}$$

Then, for all $t \ge 0$ and $i \in \{1,\ldots ,n\}$,

D327 $$\begin{aligned} 0 \le f \le N_i \quad \forall f \in {\mathcal {F}}_i(t). \end{aligned}$$

#### Proof

This was proved as Lemma B.3 in Penn and Donnelly (2022).

### Lemma 11

#### Lemma 11

For each *i*,

D328 $$\begin{aligned} \lim _{t \rightarrow \infty }(I_i(t)) = \lim _{t \rightarrow \infty }(I^V_i(t)) = 0. \end{aligned}$$

#### Proof

This was proved as Lemma B.4 in Penn and Donnelly (2022). $\square$

### Lemma 12

#### Lemma 12

Suppose that $I_i(t) > 0$ for some $t\ge 0$ and some $i \in \{1,\ldots ,n\}$. Then

D329 $$\begin{aligned} I_i(s)> 0 \quad \forall s > t. \end{aligned}$$

An analogous result holds for $I^V_i(t)$.

#### Proof

This was proved as Lemma B.5 in Penn and Donnelly (2022).

### Lemma 13

#### Lemma 13

Define

D330 $$\begin{aligned} \Pi := \left\{ i : \exists t\ge 0 \quad \text {s.t.} \quad I_i(t)> 0 \quad \text {or} \quad I_i^V(t) > 0\right\} . \end{aligned}$$

Moreover, define

D331 $$\begin{aligned} \Pi ^0 := \left\{ i : I_i(0) > 0 \right\} \end{aligned}$$

and the *n* by *n* matrix *M* by

D332 $$\begin{aligned} M_{ij} = S_i(0)\beta ^1_{ij}. \end{aligned}$$

Then, define the connected component *C* of $\Pi ^0$ in *M* as follows. The index $i \in \{1,\ldots ,n\}$ belongs to *C* if and only if there is some sequence $a_1,\ldots , a_k$ such that

D333 $$\begin{aligned} a_j \in \{1,\ldots ,n\} \quad \forall j \in \{1,\ldots ,k\}, \end{aligned}$$

D334 $$\begin{aligned} M_{a_1,a_2}M_{a_2,a_3}...,M_{a_{k-1}a_k} > 0 \end{aligned}$$

and

D335 $$\begin{aligned} a_1 = i\quad \text {and} \quad a_k \in \Pi ^0. \end{aligned}$$

Then,

$${\textbf {(a)}}$$: $$ i \in C \Rightarrow I_i(t)> 0 \quad \forall t > 0$$.
$${\textbf {(b)}}$$: $$\Pi = C \cup \Pi ^0$$.

Thus, in particular,

D336 $$\begin{aligned} i \in C\cup \Pi ^0 = \Pi \Leftrightarrow I(t)> 0 \quad \forall t > 0. \end{aligned}$$

#### Proof

This was proved as Lemma B.6 in Penn and Donnelly (2022).

### Lemma 14

#### Lemma 14

Define the set of functions

D337 $$\begin{aligned} {\mathcal {F}} := \bigg \{ S_i(t;\epsilon ), I_i(t;\epsilon ),R_i(t;\epsilon ),S^V_i(t;\epsilon ),I^V_i(t;\epsilon ),R^V_i(t;\epsilon ) : i \in \{1,\ldots ,n\}, \quad \epsilon ,t \ge 0\bigg \},\nonumber \\ \end{aligned}$$

where for each fixed $\epsilon$, these functions solve the model equations with parameters

D338 $$\begin{aligned} {\mathcal {P}}= \bigg \{\beta _{ij}^{\alpha }(\epsilon ), \mu _i^{\gamma }(\epsilon ) : i,j \in \{1,\ldots ,n\}, \quad \alpha \in \{1,2,3,4\}, \quad \gamma \in \{1,2\} \quad \text {and} \quad \epsilon \ge 0\bigg \},\nonumber \\ \end{aligned}$$

initial conditions

D339 $$\begin{aligned} {\mathcal {I}}= \bigg \{f(0;\epsilon ) : i \in \{1,\ldots ,n\}, \quad f \in {\mathcal {F}} \quad \text {and} \quad \epsilon \ge 0\bigg \} \end{aligned}$$

and vaccination policy ${\varvec{U}}(t;\epsilon )$. Suppose further that the population sizes are independent of $\epsilon$, except in group 1 where $N_1(\epsilon )$ satisfies

D340 $$\begin{aligned} |N_1(\epsilon ) - N_1(0)|\le \epsilon \quad \text {and} \quad \frac{S_1(0;\epsilon )}{N_1} = \sigma \end{aligned}$$

for some constant $\sigma$.

Suppose that

D341 $$\begin{aligned}{} & {} |p(\epsilon ) - p(0)|\le \epsilon \quad \forall p \in {\mathcal {P}}, \end{aligned}$$

D342 $$\begin{aligned}{} & {} |f_i(0;\epsilon ) - f_i(0;0)|\le \epsilon \quad \forall f \in {\mathcal {F}} \end{aligned}$$

and that

D343 $$\begin{aligned} |W_i(t,\epsilon ) - W_i(t,0)|< \epsilon \quad \forall t \ge 0. \end{aligned}$$

Moreover, suppose that for each $i \in \{1,\ldots ,n\}$ and $\epsilon \ge 0$,

D344 $$\begin{aligned} U_i(s;\epsilon ) \ge 0 \quad \text {and} \quad \int _0^t U_i(s;\epsilon ) {\textrm{d}}s \le N_i \quad \forall t \ge 0. \end{aligned}$$

Then, for each $\delta > 0$ and each $T>0$ there exists some $\eta > 0$ (that may depend on *T* and $\delta$) such that

D345 $$\begin{aligned} \epsilon \in (0,\eta ) \Rightarrow |f(t;\epsilon ) - f(t;0)|< \delta \quad \forall f \in {\mathcal {F}} \quad \text {and} \quad \forall t \in [0,T]. \end{aligned}$$

#### Proof

An almost identical result is proved in Lemma B.8 from Penn and Donnelly (2022), with the only exception being that $N_1$ can vary in this example. However, note that by replacing $\frac{S_1(0;\epsilon )}{N_1(\epsilon )}$ with $\sigma$, this lemma can be proved identically.

### Lemma 15

The following lemma is a new result, proved using similar techniques to results in Penn and Donnelly (2022) such as Lemma 13.

#### Lemma 15

Suppose that $i \in \Pi$, with $\Pi$ defined as in Lemma 13. Then, for $t > 0$,

D346 $$\begin{aligned} I^V_i(t) = 0 \Rightarrow S^V_i(t)\beta ^3_{ji} = S^V_i(t)\beta ^4_{ji} = 0 \quad \forall j \in \Pi . \end{aligned}$$

#### Proof

Suppose that there exists some *t* and some $i,j \in \Pi$ such that

D347 $$\begin{aligned} S^V_i(t)\beta ^3_{ji} > 0 \quad \text {and} \quad I^V_i(t) = 0 . \end{aligned}$$

Then, by continuity, there exists some $a < t$ such that

D348 $$\begin{aligned} S^V_i(s)\beta ^3_{ji} > 0 \quad \forall s \in (a,t). \end{aligned}$$

Moreover, by Lemma 12, it is necessary that

D349 $$\begin{aligned} I^V_i(s) = 0 \quad \forall s \in (a,t), \end{aligned}$$

while, by Lemma 13

D350 $$\begin{aligned} I_j(t) > 0 \quad \forall s \in (a,t) \end{aligned}$$

and hence (using the fact that $I^V_i(s) = 0 \quad \forall s \in (a,t)$)

D351 $$\begin{aligned} \frac{{\textrm{d}}I^V_i}{{\textrm{d}}t}\ge S^V_i(s)\beta ^3_{ji} I_j(t) > 0 \quad \forall s \in (a,t) \end{aligned}$$

and so

D352 $$\begin{aligned} I^V_i(t) > I^V_i(a) = 0, \end{aligned}$$

which is a contradiction as required. The final equality then follows as $\beta _{ji}^3 \ge \beta _{ji}^4 \ge 0$. $\square$

### Lemma 16

The following result extends the main theorem from Penn and Donnelly (2022) in a similar way to Proposition 6 to provide an additional inequality on the objective values from the optimal vaccination problem.

#### Lemma 16

Suppose that the disease trajectories ${\varvec{S}}$ and $\tilde{{\varvec{S}}}$ are given by the same model equations, parameters, vaccination policy ${\varvec{U}}$ and initial conditions except for the fact that

D353 $$\begin{aligned} S^V_1(0) > {\tilde{S}}^V_1(0). \end{aligned}$$

Then, if the objective functions are denoted by *H* and ${\tilde{H}}$ for the two policies,

D354 $$\begin{aligned} H({\varvec{U}}) \ge {\tilde{H}}({\varvec{U}}). \end{aligned}$$

#### Proof

Define a new disease model, denoted by hats where a new group $(n+1)$ is added in such that its unvaccinated compartments behave like the vaccinated compartments of group 1 and its vaccinated compartments are perfectly immune from the disease. That is,

D355 $$\begin{aligned}{} & {} {\hat{\beta }}^1_{(n+1) j} = \beta ^3_{1j}, \quad {\hat{\beta }}^2_{(n+1) j} = \beta ^4_{1j}, \quad \text {and} \quad {\hat{\beta }}^3_{(n+1) j} = {\hat{\beta }}^4_{(n+1)j} = 0 \quad \forall j \in \{1,...n\},\nonumber \\ \end{aligned}$$

D356 $$\begin{aligned}{} & {} {\hat{\beta }}^1_{j (n+1)} = \beta ^3_{j1}\quad {\hat{\beta }}^2_{j (n+1)} = \beta ^4_{j1} \quad \text {and}\quad {\hat{\beta }}^3_{j(n+1)} = {\hat{\beta }}^4_{j(n+1)} = 0 \quad \forall j \in \{1,\ldots ,n\},\nonumber \\ \end{aligned}$$

D357 $$\begin{aligned}{} & {} \beta ^{\alpha }_{(n+1) (n+1)} = 0 \quad \forall \alpha \in \{1,2,3,4\} \end{aligned}$$

and

D358 $$\begin{aligned} {\hat{\mu }}_{n+1}^1 = \mu _1^2 \quad \text {and} \quad {\hat{\mu }}_{n+1}^2 = 1. \end{aligned}$$

Suppose further that all other parameter values are identical and that the only differences in the initial conditions are that

D359 $$\begin{aligned} {\hat{S}}^V_1(0) = {\tilde{S}}^V_1(0) \quad \text {and} \quad S_{n+1}(0) =S^V_1(0) - {\tilde{S}}^V_1(0) > 0. \end{aligned}$$

Then, note that

D360 $$\begin{aligned} \frac{{\textrm{d}}({\hat{S}}^V_1 + {\hat{S}}_{n+1})}{{\textrm{d}}t}&= -\sum _{j=1}^{n+1}\bigg [{\hat{S}}^V_1({\hat{\beta }}_{1j}^3{\hat{I}}_j + {\hat{\beta }}_{1j}^4{\hat{I}}^V_j) + {\hat{S}}_{n+1}({\hat{\beta }}_{(n+1)j}^1{\hat{I}}_j + {\hat{\beta }}_{(n+1)j}^2{\hat{I}}^V_j)\bigg ]... \nonumber \\&...- \frac{{\hat{S}}_{n+1}{\hat{U}}_{n+1}}{{\hat{N}}_{n+1}-{\hat{W}}_{n+1}}\nonumber \\&= -({\hat{S}}^V_1 + {\hat{S}}_{n+1}) \sum _{j=1}^{n+1}\bigg [{\hat{\beta }}_{1j}^3{\hat{I}}_j + {\hat{\beta }}_{1j}^4{\hat{I}}^V_j\bigg ] - \frac{{\hat{S}}_{n+1}{\hat{U}}_{n+1}}{{\hat{N}}_{n+1}-{\hat{W}}_{n+1}}. \end{aligned}$$

Moreover, for $$i \ne 1$$

D361 $$\begin{aligned} \frac{{\textrm{d}}}{{\textrm{d}}t} ({\hat{S}}_i)&= -{\hat{S}}_i\sum _{j=1}^{n+1}\bigg [\beta _{ij}^1{\hat{I}}_i + \beta _{ij}^2 {\hat{I}}_i^V\bigg ] - \frac{{\hat{S}}_i{\hat{U}}_i}{{\hat{N}}_i-{\hat{W}}_i} \end{aligned}$$

D362 $$\begin{aligned}&= -{\hat{S}}_i\left( \sum _{j=2}^{n}\bigg [\beta _{ij}^1{\hat{I}}_i + \beta _{ij}^2 {\hat{I}}_i^V\bigg ] + \beta _{ij}^1{\hat{I}}_1 +\beta _{ij}^2({\hat{I}}^V_1 + {\hat{I}}_{n+1})\right) - \frac{{\hat{S}}_i{\hat{U}}_i}{{\hat{N}}_i-{\hat{W}}_i}. \end{aligned}$$

Thus, with similar calculations for ${\hat{I}}$, ${\hat{I}}^V$, ${\hat{R}}$ and ${\hat{R}}^V$, by the initial conditions and by the uniqueness of solution, in the case that ${\hat{U}}_{n+1} = 0$,

D363 $$\begin{aligned} {\hat{S}}^V_1 + {\hat{S}}_{n+1} = S^V_1\quad {\hat{I}}^V_1 + {\hat{I}}_{n+1} = I^V_1 \quad \text {and} \quad {\hat{R}}^V_1 + {\hat{R}}_{n+1} = R^V_1. \end{aligned}$$

Thus, setting

D364 $$\begin{aligned} p_{n+1} = p_1\kappa _1, \end{aligned}$$

this means that

D365 $$\begin{aligned} {\hat{H}}(\hat{{\varvec{U}}}) = H({\varvec{U}}) \end{aligned}$$

for any $\hat{{\varvec{U}}}$ such that ${\hat{U}}_{n+1} = 0$ and ${\hat{U}}_i = U_i$ for any $i \ne n$.

Now, define a vaccination policy $\hat{{\varvec{U}}}^*(t;\Delta )$ such that

D366 $$\begin{aligned} {\hat{U}}^*_i(t;\Delta ) = {\hat{U}}_i(t) \quad \forall t \ge 0 \quad \text {and} \quad i \ne n+1 \end{aligned}$$

and

D367 $$\begin{aligned} {\hat{U}}^*_{n+1}(t;\Delta )= \left\{ \begin{array}{cc} \frac{1}{\Delta }\bigg (S^V_1(0) - {\tilde{S}}^V_1(0)\bigg ) &{} \text {if } t \le \Delta \\ 0 &{} \text {otherwise} \end{array} \right. . \end{aligned}$$

Then, this means that

D368 $$\begin{aligned} {\hat{S}}_{n+1}(\Delta ;\Delta ) = 0 \quad \text {and} \quad {\hat{S}}_{n+1}^V(\Delta ;\Delta ) = S^V_1(0) - {\tilde{S}}^V_1(0) + O(\Delta ) \end{aligned}$$

while all other variable values at time $\Delta$ differ by at most $O(\Delta )$ from their initial values. Thus, define by an overbar the model given by the initial conditions which are the same as those in the hat model, but with

D369 $$\begin{aligned} {\overline{S}}_{n+1}(0) = 0 \quad \text {and} \quad {\overline{S}}^V_{n+1} = S^V_1(0) - {\tilde{S}}^V_1(0) . \end{aligned}$$

Suppose also that the vaccination policy in this case is equal to ${\varvec{U}}$, which is the pointwise limit of the vaccination policy $\hat{{\varvec{U}}}^*(t;\Delta )$ (for $t > 0$). Then, using Proposition 5, by considering the values of the variables ${\hat{f}}$ at time $\Delta$ to be the initial conditions, one finds that for any finite time *t*,

D370 $$\begin{aligned} \lim _{\Delta \rightarrow 0}({\hat{H}}({\varvec{U}}^*(t;\Delta ))) = {\overline{H}}({\varvec{U}}). \end{aligned}$$

Note this holds as it is assumed that ${\varvec{U}}$ is bounded and so

D371 $$\begin{aligned} |W_i(t+\Delta ;\Delta ) -W_i(\Delta ;\Delta ) - W_i(t)|= O(\Delta ). \end{aligned}$$

Moreover, note that the only difference between the bar model and the tilde model is in group $(n+1)$. However, by the fact that $\beta _{ij}^3 = \beta _{ij}^4 = 0$ if $(n+1) \in \{i,j\}$, the value of ${\overline{S}}^V_{n+1}$ is constant and the other variable values are independent of it. Thus, by the uniqueness of solution, this means that

D372 $$\begin{aligned} {\overline{H}}({\varvec{U}}) = {\tilde{H}}({\varvec{U}}). \end{aligned}$$

Finally, note that by Theorem 17, it must be necessary that for any $\Delta > 0$

D373 $$\begin{aligned} {\hat{H}}({\varvec{U}}(t;\Delta )) \le {\hat{H}}({\varvec{U}}(t;\infty )) = H({\varvec{U}}), \end{aligned}$$

where $\Delta = \infty$ corresponds to no vaccination taking place in group $(n+1)$ (and hence the original objective function *H* is recovered). Thus,

D374 $$\begin{aligned} {\tilde{H}}({\varvec{U}}) \le H({\varvec{U}}), \end{aligned}$$

as required.

### Theorem 17

#### Theorem 17

Suppose that ${\varvec{U}}$ and $\tilde{{\varvec{U}}}$ are feasible, bounded, Lebesgue-integrable vaccination policies. Suppose further that for each $i \in \{1,\ldots ,n\}$ and $t \ge 0$

D375 $$\begin{aligned} \int _0^t U_i(s){\textrm{d}}s \le \int _0^t {\tilde{U}}_i(s){\textrm{d}}s. \end{aligned}$$

Then, for each $t \ge 0$ and $i \in \{1,\ldots ,n\}$

D376 $$\begin{aligned} I_i(t) + R_i(t) + I^V_i(t) + R^V_i(t) \ge {\tilde{I}}_i(t) + {\tilde{R}}_i(t) + {\tilde{I}}^v_i(t) + {\tilde{R}}_i^V(t) \end{aligned}$$

and

D377 $$\begin{aligned} R_i(t) \ge {\tilde{R}}_i(t). \end{aligned}$$

Moreover,

D378 $$\begin{aligned} H({\varvec{U}}) \ge H(\tilde{{\varvec{U}}}). \end{aligned}$$

#### Proof

A proof of this theorem is given in Penn and Donnelly (2022), where it is Theorem 1. Note that the first two results are not in the statement of Theorem 1 in Penn and Donnelly (2022), but can be found at the end of the proof.

### Theorem 18

#### Theorem 18

Suppose that *B* is differentiable and that there is an optimal solution ${\varvec{U}}$. Then, define the function

D379 $$\begin{aligned} \chi (t) := \left\{ \begin{matrix} A(t) &{} \text {if} \quad \int _0^t\chi (s){\textrm{d}}s < B(t) \\ \min (A(t),B'(t)) &{} \text {if} \quad \int _0^t\chi (s){\textrm{d}}s \ge B(t) \end{matrix}\right. \end{aligned}$$

and suppose that $\chi (t)$ exists and is bounded. Then, there exists an optimal solution $\tilde{{\varvec{U}}}$ such that

D380 $$\begin{aligned} \sum _{i=1}^n{\tilde{W}}_i(t) =\max \bigg (\int _0^t \chi (s){\textrm{d}}s,1\bigg ). \end{aligned}$$

Moreover, if $\chi (t)$ is continuous almost everywhere, there exists an optimal solution $\tilde{{\varvec{U}}}$ such that

D381 $$\begin{aligned} \sum _{i=1}^n {\tilde{U}}_i(t) = \left\{ \begin{matrix} \chi (t) &{} \text {if } \int _0^t \chi (s){\textrm{d}}s < 1 \\ 0 &{} \text {otherwise}\end{matrix}\right. \end{aligned}$$

#### Proof

A proof of this theorem is given in Penn and Donnelly (2022) where it is Theorem 2.
